# The glycomic effect of N-acetylglucosaminyltransferase III overexpression in metastatic melanoma cells. GnT-III modifies highly branched N-glycans

**DOI:** 10.1007/s10719-018-9814-y

**Published:** 2018-03-03

**Authors:** Paweł Link-Lenczowski, Monika Bubka, Crina I. A. Balog, Carolien A. M. Koeleman, Terry D. Butters, Manfred Wuhrer, Anna Lityńska

**Affiliations:** 1 Department of Medical Physiology, Faculty of Health Sciences, Jagiellonian University Medical College, Michałowskiego 12, 31-126 Kraków, Poland; 2 Department of Glycoconjugate Biochemistry, Institute of Zoology and Biomedical Research, Jagiellonian University, Kraków, Poland; 3 Center for Proteomics and Metabolomics, Leiden University Medical Center, Leiden, The Netherlands; 4 Oxford Glycobiology Institute, Oxford, UK

**Keywords:** Melanoma, Glycosylation, *N*-acetylglucosaminyltransferase III, Glycome, Cancer

## Abstract

*N*-acetylglucosaminyltransferase III (GnT-III) is known to catalyze N-glycan “bisection” and thereby modulate the formation of highly branched complex structures within the Golgi apparatus. While active, it inhibits the action of other GlcNAc transferases such as GnT-IV and GnT-V. Moreover, GnT-III is considered as an inhibitor of the metastatic potential of cancer cells both *in vitro* and *in vivo*. However, the effects of GnT-III may be more diverse and depend on the cellular context. We describe the detailed glycomic analysis of the effect of GnT-III overexpression in WM266–4-GnT-III metastatic melanoma cells. We used MALDI-TOF and ESI-ion-trap-MS/MS together with HILIC-HPLC of 2-AA labeled N-glycans to study the N-glycome of membrane-attached and secreted proteins. We found that the overexpression of GnT-III in melanoma leads to the modification of a broad range of N-glycan types by the introduction of the “bisecting” GlcNAc residue with highly branched complex structures among them. The presence of these unusual complex N-glycans resulted in stronger interactions of cellular glycoproteins with the PHA-L. Based on the data presented here we conclude that elevated activity of GnT-III in cancer cells does not necessarily lead to a total abrogation of the formation of highly branched glycans. In addition, the modification of pre-existing N-glycans by the introduction of “bisecting” GlcNAc can modulate their capacity to interact with carbohydrate-binding proteins such as plant lectins. Our results suggest further studies on the biological function of “bisected” oligosaccharides in cancer cell biology and their interactions with carbohydrate-binding proteins.

**Electronic supplementary material:**

The online version of this article (10.1007/s10719-018-9814-y) contains supplementary material, which is available to authorized users.

## Introduction

Glycosylation is the most frequent and abundant post-translational modification of membrane and secreted proteins in *Eukaryota*. Because of their structural complexity, glycans bear an enormous informational capacity, strongly enhance the diversity of proteins, and thus can regulate cell biology at many levels [1]. The N-glycosylation of proteins takes place within the secretory pathway, which involves endoplasmic reticulum (ER) and Golgi apparatus (GA). The formation of mature glycans attached to the consensus sequence within the protein backbone is strongly dependent on the action of several glycosylation enzymes (glycosidases and glycosyltransferases), the accessibility of activated monosaccharide donors and the efficiency of their transportation into the lumen of ER and GA [2]. Although the general orchestration of the glycosylation machinery has been well described [3], the details of the regulation of the process are still under investigation. One of the most interesting aspects is the mutual relationship between glycosyltransferases within the Golgi apparatus.

*N-*Acetylglucosaminyltransferase III (GnT-III) is an enzyme acting within the *medial*-Golgi and is responsible for the transfer of a single *N*-acetylglucosamine (GlcNAc) in a β1,4 linkage to the β-mannose on N-glycans, thus forming the “bisecting” GlcNAc structure. The presence of the “bisecting” GlcNAc, which causes a characteristic conformational change of the glycan [4], has been recognized as inhibitory towards other GlcNAc transferases such as GnT-IV and GnT-V, preventing the formation of highly-branched species, such as the β1-6-GlcNAc branching catalyzed by GnT-V [5–7]. Moreover, recent data show that overexpression of GnT-III can dramatically suppress α2-3-sialylation at a post-transcriptional level [8].

The action of GnT-III also has important implications in cancer biology, since the transformation and progression of many types of cancer are accompanied by changes in protein glycosylation, some of which are considered cancer biomarkers [9–11]. In this regard, GnT-III traditionally is pictured as a suppressor of malignancy and there is much evidence that overexpression of GnT-III inhibits the metastatic potential of cancer cell both *in vitro* and *in vivo* [6, 12, 13]. This anti-cancer effect of GnT-III activity is often attributed to the fact that the presence of “bisecting” GlcNAc on membrane adhesion proteins such as cadherins and integrins modulates their function and thus influence adhesion relations between cancer cells themselves as well as between cells and the extracellular matrix proteins [14, 15]. Previous studies clearly show that GnT-III activity promotes homotypic interactions of E-cadherins on mammary carcinoma and melanoma cells [14, 16]. Downregulation of *Mgat3* in the mouse model of PyMT-induced mammary carcinoma accelerates migratory properties of cancer cells promoting the metastases to the lung [17]. Finally, it is known that introduction of “bisecting” GlcNAc to integrins reduces cell migration [18]. The metastasis-inhibitory effect of GnT-III, also in the context of adhesion protein modulation is linked to its ability to inhibit the formation of highly branched N-glycans, especially β1-6-GlcNAc branching with polylactosamine epitopes, which can promote cell proliferation, migration and invasion [6]. However, the picture of GnT-III as a universal metastasis modulator remains controversial, as its elevated activity is observed in some malignancies such as hepatoma, ovarian cancer, multiple myeloma and chronic myeloid leukemia [19–22]. These observations suggest, that the GnT-III effect in cancer is more complex, and may depend on the cellular context. Hence, its impact on the repertoire of N-glycans on the cell surface and on secreted proteins needs further study.

Melanoma is a highly invasive tumor, which can develop within the skin, uvea and gastric mucosa [23]. Many studies suggest the important role of β1-6-GlcNAc branched N-glycans in the promotion of metastatic potential of melanoma cells, mainly through the modulation of integrin-dependent adhesion and migration [24–27]. Recently, we have developed an *in vitro* melanoma model in which we induced the overexpression of GnT-III in metastatic melanoma cell line WM266–4 [28]. In the present work, we have investigated the N-glycosylation profile of membrane and secreted proteins of these cells in detail, providing evidence that GnT-III upregulation does not inhibit the formation of highly branched N-glycans but efficiently modifies these glycans by the introduction of a “bisecting” GlcNAc.

## Materials and methods

### Reagents

Nonidet P40 was purchased from Roche (Warszawa, Poland). Acrylamide, APS, bisacrylamide, 0.5 M Tris pH 6.8 buffer, 1.5 M Tris pH 8.8 buffer, Laemmli Sample Buffer, 2-mercaptoethanol, TEMED, Tris-glycine buffer, Tris-glycine-SDS buffer were procured from Bio-Rad (Warszawa, Poland). PageRuler Prestained Protein Ladder was obtained from Fermentas (Thermo Fisher Scientific, Warszawa, Poland). DPBS and FBS were purchased from Life Technologies (Warszawa, Poland). 2,5-dihydroxybenzoic acid (2,5-DHB) was from Bruker Daltonics (Bremen, Germany). Acetonitrile HPLC grade for far UV, 2-aminobenzamide (2-AB), anthranilic acid (2-AA), sodium cyanoborohydride, DMSO and trifluoroacetic acid (TFA) were purchased from Sigma-Aldrich (Poznań, Poland). All other salts, alcohols and acids were analytical grade chemicals from Sigma-Aldrich. Water used was of Milli-Q grade.

### Cells and culture conditions

As an experimental model, we used previously described WM-266-4-GnT-III human metastatic melanoma cells, stably overexpressing the *MGAT3* gene together with mock control cells WM-266-4-pIRESneo [28]. The cells were grown in RPMI-1640 culture medium supplemented with 25 mM HEPES and L-alanine-L-glutamine (RPMI-1640 Glutamax-I; Gibco, Life Technologies), in the presence of standard antibiotic cocktail (100 μg/ml streptomycin, 100 U/ml penicillin; Sigma-Aldrich) and 100 μg/ml G418 sulfate (Geneticin; Gibco, Life Technologies) as a selection agent. Culture medium was supplemented with 10% fetal bovine serum (Gibco, Life Technologies) and cells were grown with 5% CO_2_ at 37 °C. Cells were systematically tested by PCR for the presence of *Mycoplasma sp*.

### Isolation of membrane and secreted proteins

Cells were grown until ~70% confluency, washed 5 times with DPBS and then cultured in serum-free medium for 24 h. The culture media containing secreted proteins were collected, filtered through a 0.4 μm pore size syringe filter and frozen at −70 °C. The cells were washed 3 times with cold DPBS, scraped carefully with a rubber policeman in about 2 ml of cold DPBS and centrifuged at 500×*g* for 10 min at 4 °C. The membrane proteins were isolated using QProteome Cell Compartment Kit (Qiagen, Hilden, Germany) according to manufacturer’s protocol and the obtained fractions were frozen at −70 °C.

Before electrophoresis and deglycosylation, the secreted and membrane proteins were concentrated by precipitation. Briefly, the collected conditioned media containing secreted proteins were lyophilized, resuspended in a minimal volume of 50 mM Tris-HCl, pH 8.0 containing 0.5% Triton-X-100 and dialyzed 3 times overnight against water. For protein precipitation, one part of secreted proteins suspension or membrane protein fraction was mixed with 4 parts of methanol, followed by 1 part of chloroform and 3 parts of water, with mixing at each step. After centrifugation (15,000×*g*, 2 min), the upper aqueous layer was removed, leaving the proteins intact at the interphase. Then, 4 volumes of methanol were added, and the precipitated proteins were spun down (15,000×*g*, 2 min) and dried at room temperature (RT) under the fume hood, after removal of the supernatant.

### Polyacrylamide gel electrophoresis in presence of SDS (SDS-PAGE)

Precipitated protein pellets were resuspended in Laemmli sample buffer, and after heat denaturation (100 °C, 5 min) the proteins were separated on 8% polyacrylamide gels, according to Laemmli [29] in the presence of 0.1% SDS and beta-mercaptoethanol. Molecular masses of proteins were determined using a prestained protein ladder (Fermentas). After electrophoresis, separated proteins were transferred onto an Immobilon-P PVDF membrane (Merck Millipore, Warszawa, Poland) overnight at 4 °C with a constant current of 100 mA (Mini Trans-Blot Electrophoretic Transfer Cell; Bio-Rad) according to Towbin et al. [30]*.*

### Lectin detection of blotted glycoproteins

The protein bands containing studied oligosaccharide epitopes were detected according to Haselbeck et al. [31] with further modifications made by Ochwat et al. [32]. PVDF membranes with separated glycoproteins were blocked in 0.5% blocking reagent (DIG Glycan Differentiation Kit; Roche Diagnostics, Mannheim, Germany) in TBS for 2–3 h at RT. Blots were washed twice in TBS-0.1% Tween 20 and once in lectin incubation buffer. Membranes were incubated (1 h, RT) with following biotinylated lectins (Vector Labs, Burlingame, CA): *Phaseolus vulgaris* erythroagglutinin (PHA-E), *Phaseolus vulgaris* leucoagglutinin (PHA-L), *Sambucus nigra* agglutinin (SNA), *Maackia amurensis* agglutinin (MAA), *Galanthus nivalis* agglutinin (GNA) and *Aleuria aurantia* agglutinin (AAA) (see Table 1). All lectins were diluted (1:4000) in 50 mM Tris-HCl, pH 7.5 containing 150 mM NaCl, 1 mM MgCl_2_, 1 mM MnCl_2_, 1 mM CaCl_2_. After incubation with lectins, membranes were washed (3 × 15 min) in TBS-0.1% Tween 20 and incubated (1 h, RT) with ExtrAvidin-AP (Sigma-Aldrich) diluted in TBS-0.1% Tween 20 (1:4000). After another washing series (3 × 5 min in TBS-0.1% Tween 20; 3 × 5 min in TBS), lectin-reactive bands were visualized on membranes after transformation of NBT and BCIP 4-toluidine salt substrates (Roche Diagnostics) into colored products.

**Table 1:** List of plant lectins used in lectin blot assay

Lectin *source*	Binding specificity
SNA-I *Sambucus nigra*	**NeuNAcα2-6**Gal- **NeuNAcα2-6**GalNAc-
MAA-I *Maackia amurensis*	**NeuNAcα2-3**Galβ1-4GlcNAc
PHA-L *Phaseolus vulgaris*	Galβ1-4**GlcNAcβ1-6**(Galβ1-4GlcNAcβ1-2)Manα-
PHA-E *Phaseolus vulgaris*	Galβ1-4GlcNAcβ1-2Manα1-4 (**GlcNAcβ1-4**)Manα-
GNA *Galanthus nivalis*	**Manα1-2**Manα- **Manα1-6**Manα- **Manα1-3**Manα-
AAA *Aleuria aurantia*	**Fucα1-2**Galβ- **Fucα1-6**GlcNAcβ- **Fucα1-3**GlcNAcβ-

### Isolation and labeling of glycoprotein derived N-linked oligosaccharides

Precipitated proteins were resuspended in 20 μl of reducing buffer (0.5% SDS, 1% 2-mercaptoethanol), denatured at 100 °C for 10 min and after cooling to RT, SDS was neutralized by adding 4 μl of 10% NP-40. After that, 4 μl of 0.5 M sodium phosphate buffer, pH 7.5 was added followed by 2 μl of PNGase F (500,000 U/ml, New England Biolabs, Ipswich, MA) and water up to 40 μl. The glycoprotein samples were deglycosylated at 37 °C overnight. The released N-glycans were desalted by solid phase extraction (SPE) on non-porous graphitized carbon SPE columns (Grace, Alltech, Columbia, MD) according to Packer et al. [33] and eluted glycans were dried-down on SpeedVac. Next, samples were made up to 30 μl with water and the released oligosaccharides were labeled with anthranilic acid (2-AA) according to Anumula and Dhume [34] modified by Neville et al. [35]. Fluorescently labeled N-glycans were then purified on *Spe-ed* Amide-2 SPE columns (Pelican Scientific, Tattenhall, U.K.). The columns were washed with 1 mL of ACN followed by 1 mL of water and equilibrated with 2 mL of ACN. The samples were diluted with 1 mL of 97% (*v/v*) ACN in water and loaded on columns. After washing two times with 95% (*v/v*) ACN in water the labeled N-glycans were eluted with 1.5 mL of water and kept frozen at −20 °C.

### Separation of charged and neutral N-glycans prior the HPLC analysis

Negatively charged oligosaccharides were separated from neutral species by SPE using anion exchange resin (QAE-Sephadex, Sigma-Aldrich, Poole, U.K.). 1 mL plastic mini-columns were loaded with 200 μl of the resin and washed with 2 mL of water. Aliquots of 2-AA labeled N-glycans were loaded on columns and after washing with water the neutral glycans were eluted with 0.5 M acetic acid and the charged fraction with 0.5 M ammonium acetate. The neutrals were purified by lyophilization and the charged species were desalted using PGC SPE (HyperSep® HyperCarb®; Thermo Fisher Scientific, Paisley, UK) according to Alonzi et al. and dried under vacuum [36].

### HPLC analysis of fluorescently labeled N-glycans

Purified 2-AA oligosaccharides were separated by NP-HPLC using a 4.6 × 250 mm TSK gel-Amide 80 column (5 μm bead size) (Anachem, Luton, Beds, U.K.) according to Neville et al. [37]. The chromatography system consisted of Waters Alliance 2695 separations module and an in-line Waters 474 fluorescence detector set at Exλ 360 nm and Emλ 425 nm. All chromatography was performed at 30 °C. Solvent A was acetonitrile. Solvent B was Milli-Q water. Solvent C was composed of 800 mM ammonium hydroxide, titrated to pH 3.85 with acetic acid, in Milli-Q water. Samples were loaded in 70% ACN and separated using gradient conditions as follows: time = 0 min (*t* = 0), 71.6% A, 25.9% B, 2.5% C (0.8 mL/min); *t* = 6, 71.6% A, 25.9% B, 2.5% C (0.8 mL/min); *t* = 45, 46.2% A, 51.3% B, 2.5% C (0.8 mL/min); *t* = 46, 35% A, 62.5% B, 2.5% C (0.8 mL/min); *t* = 48, 35% A, 62.5% B, 2.5% C (0.8 mL/min); *t* = 49, 71.6% A, 25.9% B, 2.5% C (0.8 mL/min); *t* = 51, 71.6% A, 25.9% B, 2.5% C (1.2 mL/min); *t* = 64, 71.6% A, 25.9% B, 2.5% C (1.2 mL/min); *t* = 65, 71.6% A, 25.9% B, 2.5% C (0.8 mL/min). All chromatography was controlled, and data were collected and processed using Waters Empower software, and the glucose unit values were determined following comparison with a 2AA-labeled glucose oligomer ladder external standard (Glyko Prozyme, Hayward, CA).

### MALDI-TOF(/TOF)-MS

Dried 2-AA labeled glycans were reconstituted in a minimal volume of water and desalted using a C_18_ ZipTip™ (Millipore) following the manufacturer’s instructions. Glycans were eluted with 1.5 μl of 2,5-dihydroxybenzoic acid (10 mg/ml in 50/50, ACN/water containing 0.1% TFA) directly onto a MALDI target plate and dried at RT.

MALDI-TOF-MS was performed on UltrafleXtreme™ mass spectrometer controlled by FlexControl 3.1 software (Bruker Daltonics, Bremen, Germany). The instrument was externally calibrated using the Bruker peptides calibration kit. The spectra were acquired both in the negative ion reflectron mode and linear mode over the *m*/*z* range from 700 to 5000 for a total of 5000 shots.

### LC-ESI-ion trap-MS/MS

Nano-liquid chromatography-tandem mass spectrometry (nanoLC-MS/MS) was performed with the use of an Ultimate 3000 LC system (Dionex, Amsterdam, The Netherlands). Aliquots of the 2-AA labeled N-glycans (1 μl) were applied to a C_18_ PepMap™ 0.3 × 5 mm trapping column (Dionex) and washed with 100% solvent A (0.1% formic acid in water and 0.4% ACN) for 10 min at a flow rate of 25 μl/min. Next, AA-labeled glycans were separated on a reverse phase analytical column (C_18_ PepMap 100 Å, 3 μm, 75 μm × 150 mm; Dionex) at a flow rate of 300 nl/min with the UV detection. The mobile phase gradient was as follows: 0–25% eluent B (95% ACN, 5% water) in 15 min and 25–70% eluent B in the next 10 min, followed by an isocratic elution with 70% eluent B for 5 min. The LC system was coupled via an online nanospray source to an Esquire HCT Ultra ESI-ion trap-MS (Bruker Daltonics). For electrospray (1100–1250 V), stainless steel capillaries with an inner diameter of 30 μm (Proxeon, Odense, Denmark) were used. The dry gas temperature was set to 165 °C, and the nitrogen stream was set to 7 l/min. The glycan spectra were acquired in positive-ion mode and the mass spectrometer was carefully tuned to minimize glycan decay in the ion transfer region, thanks to the following settings: skimmer, 40 V; capillary exit, 106 V; octopole 1 DC, 6 V. The AA-labeled glycans were analyzed using the data-dependent MS/MS mode over a 300–1500 *m*/*z* range. Five of the most abundant ions in an MS spectrum were selected for MS/MS analysis by collision-induced dissociation (CID). The LC-MS system was tuned to minimize the effect of in-source decay of sialylated structures as described previously [38].

### Statistical methods

Unless otherwise indicated, the Student’s t-test was performed to determine statistical significance between the average of 3 replicates.

## Results

Initially, to check the effect of transfection on glycosylation status in studied cells we analysed the MALDI-TOF-MS spectra of parent and transfected cells (Supplementary Fig. 2). We did not observe any significant effect of the transfection itself. In further studies we used mock-transfected cell line as a negative control.

### Characterization of N-glycan epitopes on membrane and secreted proteins using plant lectins

**Fig. 1:**
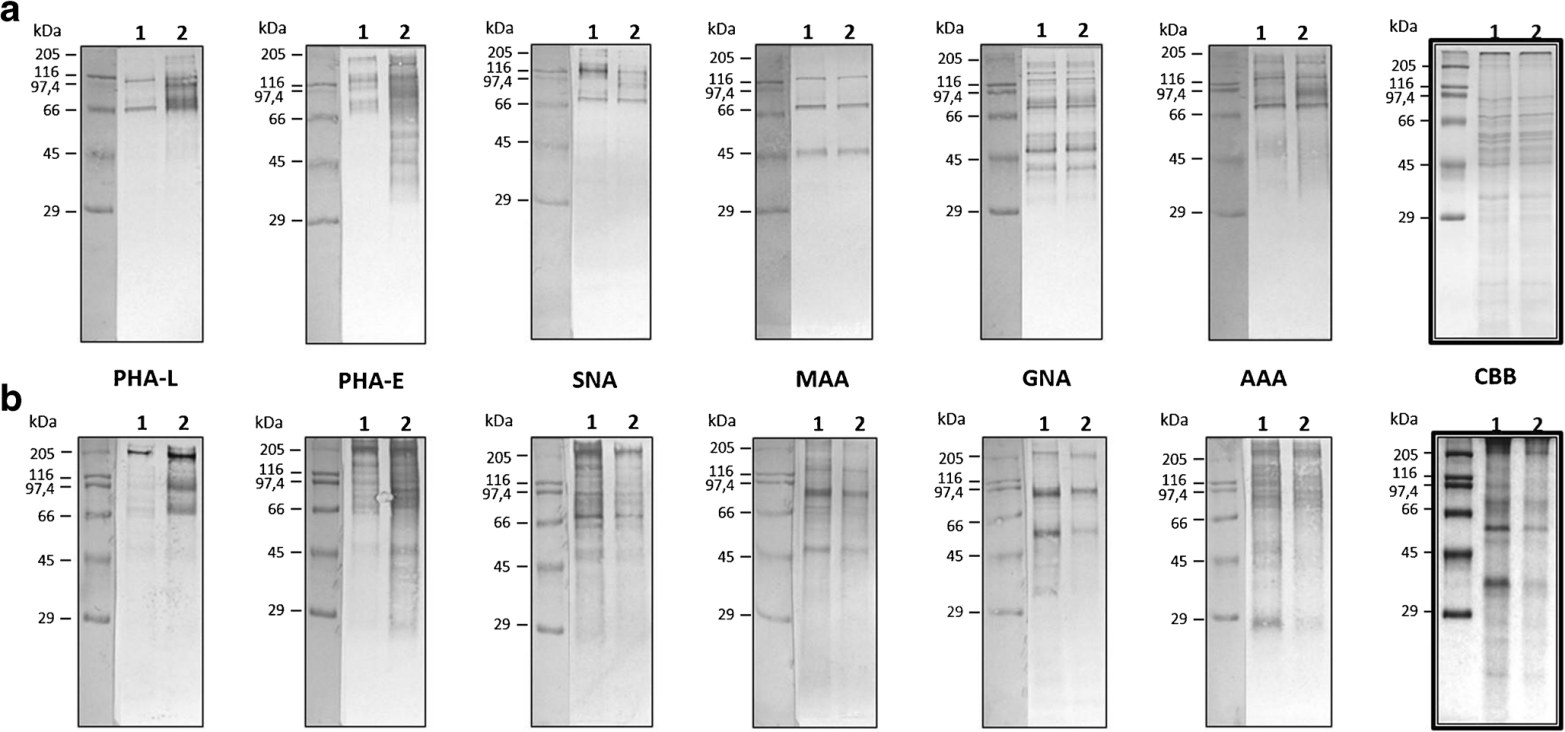
**Lectin detection of glycoproteins blotted on PVDF membrane.** Membrane proteins (**a**) and secreted proteins (**b**) isolated from control cells (**1**: WM266–4-pIRESneo) and GnT-III overepressing cells (**2**: WM266–4-GnT-III) were resolved by SDS-PAGE and blotted glycoproteins were probed with specific lectins (see Table 1). CBB – Coomasie Brilant Blue-stained blot. As the buffers used for extraction of subcellular protein fractions were not compatible with any of the protein assay, the samples were prepared by volume and the optical density of positive protein bands was than normalized to CBB staining (Fig. 2)

**Fig. 2:**
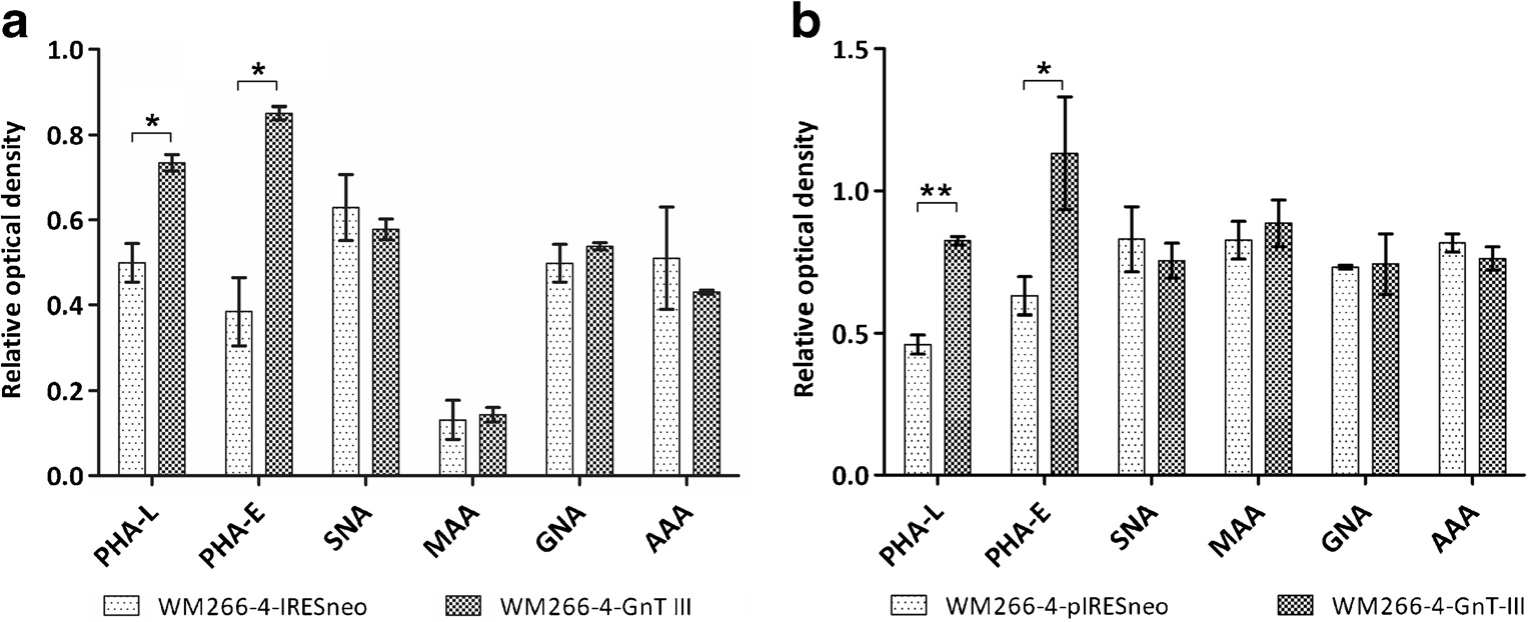
**Relative optical density of lectin-positive glycoprotein bands on PVDF membrane.** Membrane proteins (**a**) and secreted proteins (**b**) isolated of control cells (WM266–4-pIRESneo) and GnT-III overepressing cells (WM266–4-GnT-III) were resolved by SDS-PAGE and blotted glycoproteins were probed with specific lectins. Optical density of each lane was measured and presented in relation to optical density of loading control (CBB stained blots). Three independent experiments were performed. Data are presented as means +/− standard deviation (SD). Asterisk indicates statistically significant difference (*p < 0.05, ** *p* < 0.01; Student t-test)

As an initial characterization of the impact of upregulation of GnT-III on the N-glycan repertoire in melanoma cells, we evaluated the binding of selected plant lectins to membrane and secreted glycoproteins blotted onto PVDF membrane. We chose an array of lectins recognizing most characteristic glycan epitopes (see Table 1). As the buffers used for extraction of subcellular protein fractions were not compatible with any of the protein assay, to avoid the effect of inequality of protein load the lectin binding strength was calculated as a relative optical density of all reactive protein bands in each lane with the Coomassie Brilliant Blue (CBB) stained protein lanes as a loading control. We observed similar lectin binding patterns in the case of membrane and secreted proteins, suggesting a similar glycosylation status of glycoproteins in both protein groups. Overexpression of GnT-III did not appear to influence the binding strength of SNA and MAA to membrane and secreted proteins, suggesting no differences in the amount of α2–6 and α2–3 sialylated glycans, respectively (Figs. 1 and 2). A similar observation was done in the case of GNA staining, recognizing high-mannose N-glycans (Figs. 1 and 2) and AAA, which specifically binds to fucose (Figs. 1 and 2). PHA-E staining (detecting “bisecting” GlcNAc) was significantly increased in the case of both protein groups isolated from WM266–4-GnT-III cells in comparison to control cells (Figs. 1 and 2). Surprisingly, the staining pattern with PHA-L (Figs. 1 and 2), which specifically recognizes β1–6 branching of complex N-glycans was similar, suggesting an elevation of PHA-L-reactive glycoproteins or glycans upon overexpression of GnT-III.

### HILIC-HPLC analysis of N-glycans derived from membrane and secreted proteins

**Fig. 3:**
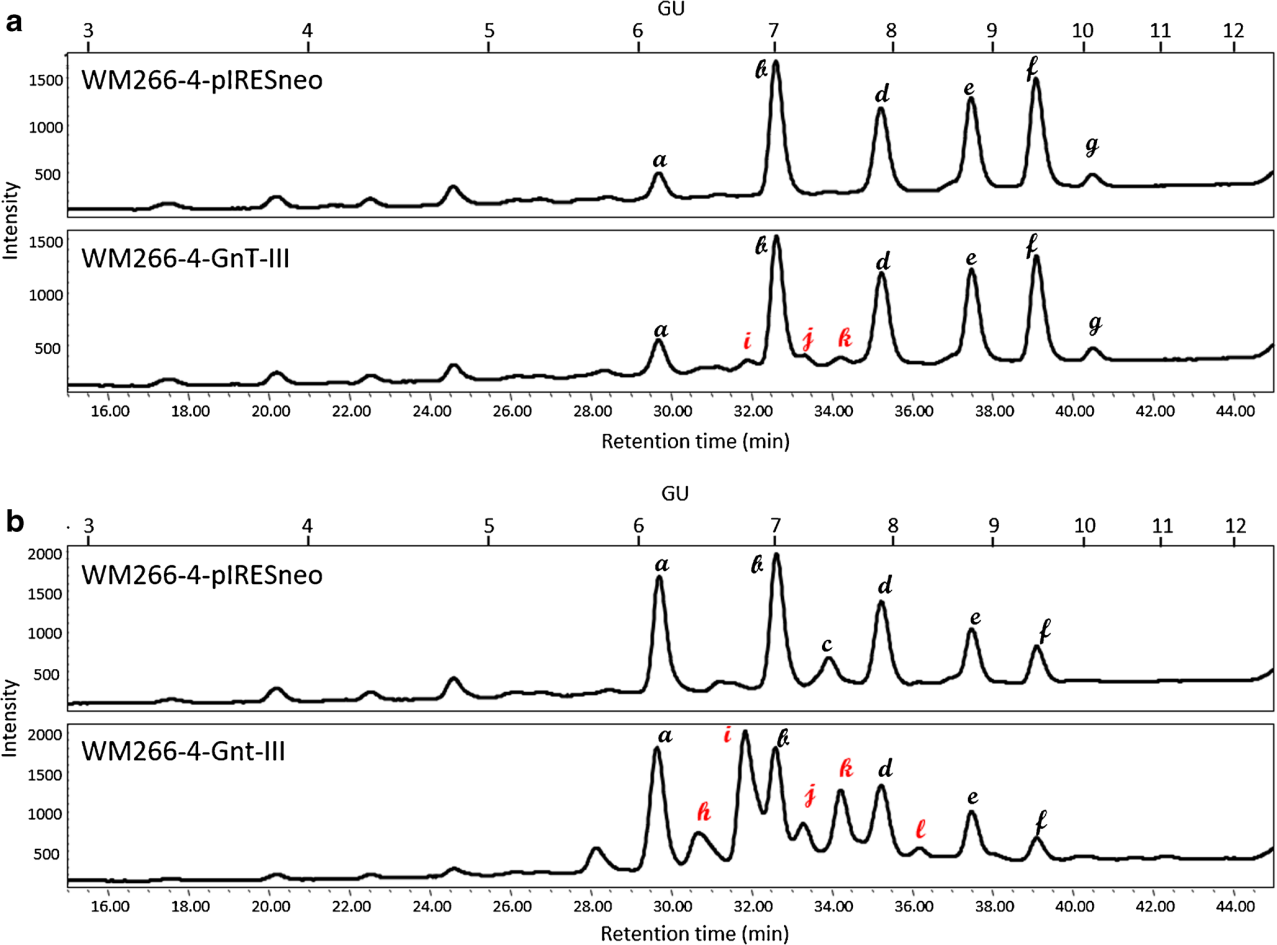
**HILIC-HPLC separation of neutral N-glycans.** Membrane proteins (**a**) and secreted proteins (**b**) of control cells (WM266–4-pIRESneo) and GnT-III overepressing cells (WM266–4-GnT-III) were deglycosylated and 2-AA labelled neutral glycans were separated on TSKgel Amide-80 column. The major peaks are indicated with letters. The black letters represent the peaks present in case of both cell lines, red letters indicate peaks characteristic only for cells that overexpress GnT-III. GU - glucose units

To study the glycosylation changes upon GnT-III overexpression in more details, we performed HILIC-HPLC of 2-AA labeled N-oligosaccharides derived from membrane and secreted proteins. In case of membrane protein-derived neutral N-glycans the most abundant, both in case of transfected cells and in the control, were 5 peaks a, b, d, e, f and g (Fig. 3a) with GU values of 6.13, 7.00, 7.87, 8.72, 9.40 and 10.05, respectively. Similarly, in case of secreted protein glycans, the most intensive fluorescence represented peaks a, b, d, e, f with no peak g present, but with additional peak c with GU of 7.43 (Fig. 3b). Overexpression of GnT-III caused the appearance of additional peaks (Fig. 3), which in case of membrane proteins have GU values of 6.78, 7.22 and 7.52 (peaks i, j and k, respectively; Fig. 3a) and in case of secreted protein derived glycans have GU values of 6.41, 6.76, 7.22, 7.53, 8.22 (peaks h, i, j, k, and l, respectively, Fig. 3b). These additional peaks represented approximately 9% of all neutral species from membrane proteins, whereas in case of secreted proteins approximately 47% of all neutral oligosaccharides.

**Fig. 4:**
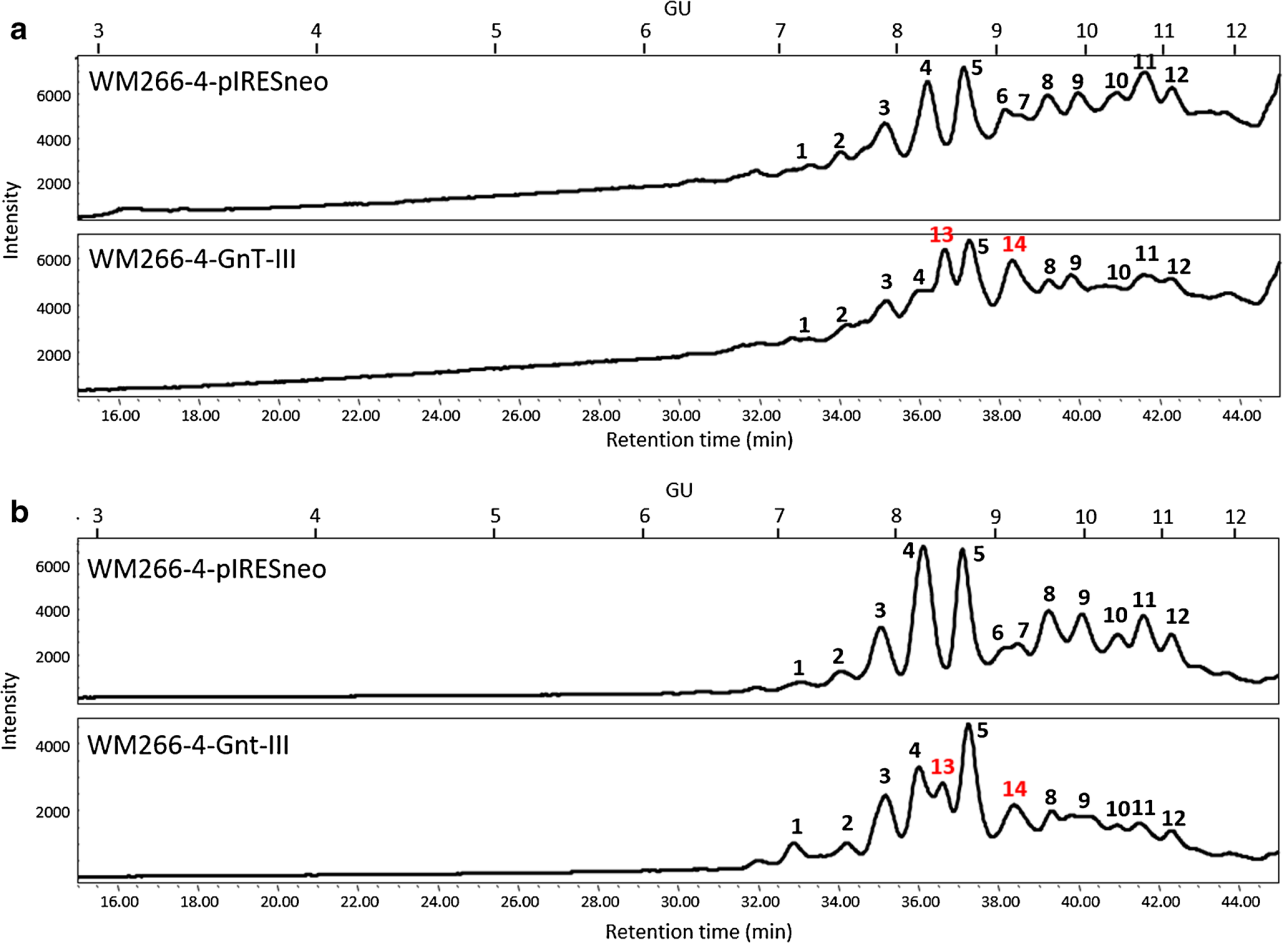
**HILIC-HPLC separation of negatively charged N-glycans.** Membrane proteins (**a**) and secreted proteins (**b**) of control cells (WM266–4-pIRESneo) and GnT-III overepressing cells (WM266–4-GnT-III) were deglycosylated and 2-AA labelled charged glycans were separated on TSKgel Amide-80 column. The major peaks are indicated with numbers. The black numbers represent the peaks present in case of both cel lines, red numbers indicate peaks characteristic only for cells that overexpress GnT-III. GU - glucose units

A similar analysis was made when it comes to negatively charged N-glycans from both protein groups (Fig. 4). GnT-III upregulation resulted in the appearance of additional peaks 13 and 14 with GU values of 8.39 and 9.07, respectively. The relative abundance of these additional peaks represents approx. 32% of all charged glycans in case of membrane proteins, and approx. 12% of charged species from secreted glycoproteins.

**Fig. 5:**
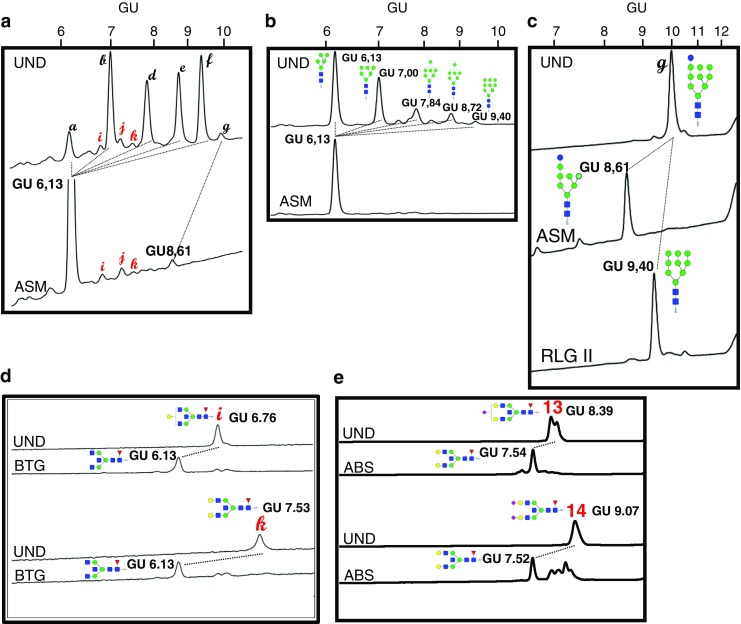
**HILIC-HPLC characterisation of selected neutral and charged N-glycan peaks from WM266–4-GnT-III cells.** Proteins were deglycosylated and 2-AA labelled N-glycans were separated on TSKgel Amide-80 column before and after digestion with exoglycosidases (see Table 2). Panel **a** represents the effect of neutral N-glycan pool treated with α1–2 mannosidase (ASM). Panel **b** shows the effect of digestion of ribonuclease B high mannose glycan standard with ASM. Panel **C** represents the effect of ASM and α1–3 glucosidase II (RLGII) treatment on isolated peak **g**. Panel **d** represents the effect of β1–3,4 galactosidase (BTG) treatment on isolated peaks i and k. Panel **e** represents the effect of α2-3-6 sialidase (ABS) treatment on isolated peaks 13 and 14. UND – undigested, GU - glucose units

To elucidate the structures of glycans represented by selected peaks, HILIC-HPLC analysis of glycan pools or collected individual peaks treated with specific exoglycosidases was performed (for enzymes used in the study see Table 2). After digestion of neutral species with *Aspergillus saitoi* α1–2 mannosidase (ASM) the peaks b, d, e and f collapsed to a single peak of 6.13 GU corresponding to MAN_5_, relying on the ribonuclease B glycan library digestion as an external standard (Fig. 5a and b). Thus, the identity of the peaks b, d, e and f as high mannose structures (MAN_6_, MAN_7_, MAN_8_ and MAN_9_, respectively) was confirmed. The single peak specific only for the membrane protein glycan fraction (peak g), was also susceptible to ASM treatment and collapsed to a single peak of 8.61 GU (Fig. 5a). This suggested the presence of the terminal glucose on the MAN_9_ glycan (GlcMAN9) in this case. To prove this, the peak was manually collected and treated additionally with rat liver α1–3 glucosidase II (RLG II), and after digestion the GU shift from 10.05 to 9.40 (corresponding to MAN_9_) was observed (Fig. 5c).

**Table 2:** List of exoglycosidases used to study the structures of N-glycans by HILIC-HPLC

Exoglycosidase *source*	Abbreviation
α1-2 mannosidase *Aspergillus saitoi*	ASM
β3-4 galactosidase bovine testes	BTG
α,1-3 glucosidase II rat liver	RTG
α2-3-6 sialidase *Arthrobacter ureafaciens*	ABS

The peaks, that were characteristic only for transfected cells (WM266–4-GnT-III) were also partially characterized with the use of bovine testes β1-3-4 galactosidase (BTG), *Arthrobacter ureafaciens* α2-3-6 sialidase (ABS) and ASM. As the main 3 peaks of neutral species (peaks i, j and k, Fig. 3) did not shift after mannosidase treatment (Fig. 5a), they were probably complex N-glycans. On the basis of the effect of BTG digestion and GU values of isolated peaks i and k, they were identified as diantennary fucosylated structures with “bisecting” GlcNAc and one (Hex_4_NexNAc_5_Fuc_1_-2-AA) or two terminal galactoses (Hex_5_HexNAc_5_Fuc_1_-AA), respectively (Fig. 5d). In terms of charged N-glycans of transfected cells the two characteristic peaks (peak 13 and 14) both collapsed after ABS treatment and, on the basis of their GU values they were annotated as diantennary fucosylated structures with “bisecting” GlcNAc and one (Hex_5_NexNAc_5_Fuc_1_Sia_1_-2-AA) or two (Hex_5_NexNAc_5_Fuc_1_Sia-2-AA) terminal sialic acid residues (Fig. 5e).

### Identification of N-glycans from membrane and secreted proteins by mass spectrometry

**Fig. 6:**
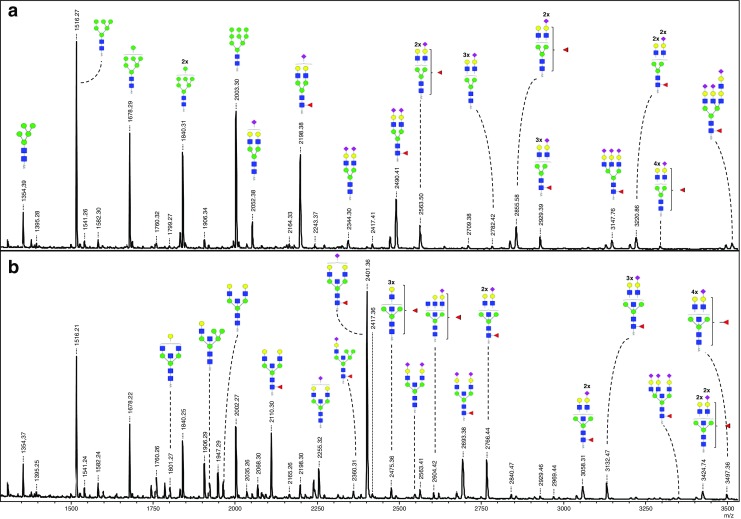
**Negative ion mode MALDI-TOF-MS spectra of AA-labeled*N*-glycans released from membrane proteins of WM266–4-pIRESneo (a) and from WM266–4-GnT-III (b) melanoma cells.** Proposed composition of the *N*-glycan structures deduced from the MALDI-TOF-MS and LC-ESI-ion-trap-MS/MS are listed in supplementary Table 1. Only major structures are depicted. Only the *m*/*z* values which are considered as an transfection effect are annotated in **b**. In some cases structural isomers are possible. Fucose linkage is specified only in cases where LC-ESI-ion-trap-MS/MS gives evidence. Glycan schemes were prepared using GlycoWorkbench. The spectra and *m/z* values obtained in the MALDI-TOF linear mode are presented here. *Red triangle*, fucose; *yellow circle*, galactose; *green circle*, mannose; *blue square*, *N-*acetylglucosamine; *purple diamond*, sialic acid

**Fig. 7:**
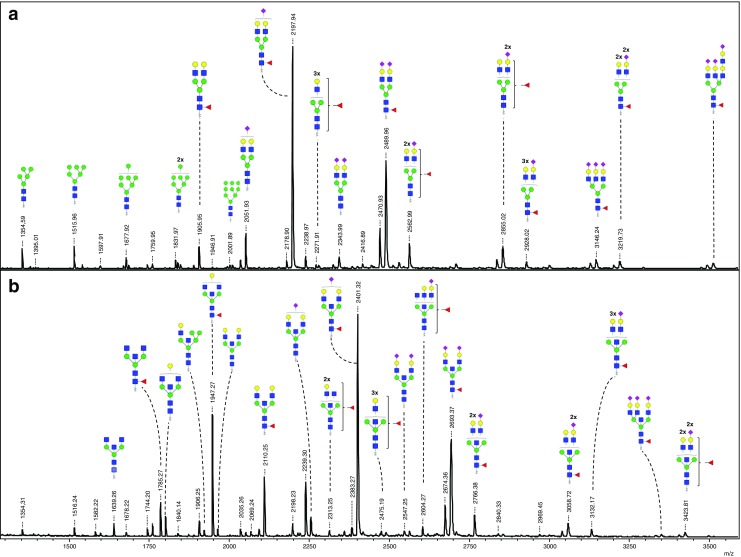
**Negative ion mode MALDI-TOF-MS spectra of AA-labeled*N*-glycans released from proteins secreted by WM266–4-pIRESneo (a) and WM266–4-GnT-III (b) melanoma cells.** Proposed composition of the *N*-glycan structures deduced from the MALDI-TOF-MS and LC-ESI-ion-trap-MS/MS are listed in supplementary Table 1. Only major structures are depicted. Only the *m*/*z* values which are considered as an transfection effect are annotated in **b**. In some cases structural isomers are possible. Fucose linkage is specified only in cases where LC-ESI-ion-trap-MS/MS gives evidence. Glycan schemes were prepared using GlycoWorkbench. The spectra and *m/z* values obtained in the MALDI-TOF linear mode are presented here. *Red triangle*, fucose; *yellow circle*, galactose; *green circle*, mannose; *blue square*, *N*-acetylglucosamine; *purple diamond*, sialic acid

*2-AA labeled N*-glycans from both cell lines and both protein groups were further purified and analyzed by MALDI-TOF-MS (Figs. 6 and 7). In addition, to elucidate structural features of the N-glycans all 4 samples were studied by LC-ESI-ion trap-MS/MS. The fragmentation spectra of selected N-glycans are gathered in Supplementary Fig. 1. The glycans identification was based on fragmentation of selected glycans and common knowledge of glycobiology. The Consortium for Functional Glycomics (CFG) notation was used for the schematic representation of N-glycans with in most cases no specification of the linkage position in case of isomeric structures, unless indicated otherwise.

The structural annotation of glycans is summarized in Supplementary Table 1. In general, 90 different N-glycan structures were identified, with 29 of them present only in case of WM266–4-GnT-III cells. Thus, the appearance of these structures may be attributed to the overexpression of GnT-III in melanoma cells. The major peaks within the N-glycans of membrane proteins isolated from control cells were identified as high mannose structures MAN_6_, MAN_7_, MAN_8_ and MAN_9_ with *m/z* 1516.45, *m/z* 1678.55, *m/z* 1840.60 and *m/z* 2002.64, respectively. This is consistent with the data from HILIC-HPLC analysis, where these structures were also identified as major peaks within the chromatogram (Fig. 5). Another high mannose glycan was also identified at *m/z* 1354.39 as MAN_5_, together with the monoglucosylated MAN_9_ glycan at *m/z* 2164.72, in line with the results of the HPLC analysis (Fig. 5). The sum of relative abundances of all high-mannose N-glycan peaks within the MALDI-MS spectrum of membrane protein derived glycans was 58%. Additionally, unusual small paucimannosidic structures with or without core fucose were identified (eg. Hex_4_HexNAc_2_-AA and Hex_3_HexNAc_2_dHex_1_-2-AA at *m/z* 1192.35 and *m/z* 1176.36, respectively). Other major peaks that were observed in case of the membrane protein fraction of control cells were at *m/z* 2051.69, *m/z* 2197.76, *m/z* 2488.92, *m/z* 2562.88, *m/z* 2854.07, *m/z* 2927.98, *m/z* 3145.19 and *m/z* 3219.15. These peaks were mainly core-fucosylated complex N-glycans with terminal sialic acid residues (1–3) and different number of *N*-acetyllactosaminyl (LacNAc) units or antennae (2–4) (Supplementary Table 1), and the sum of their relative abundances within the spectrum was 23%. Among these glycans, the peak at *m/z* 3145.19 was annotated as triantennary structure containing 3 terminal sialic acid residues (Hex_6_HexNAc_5_dHex_1_NeuNAc_3_-AA). Another peak with 3 or 4 LacNAc units can represent either tri- or tetra-antennary glycans or diantennary structures with poly-LacNAc extensions of the antennae. Taking into account the MS/MS data we observe in these cases clear intensive signals at *m/z* 366.0 [M + H]^+^ (Hex_1_HexNAc_1_) and *m/z* 657.2 [M + H]^+^ (Hex_1_HexNAc_1_NeuNAc_1_) indicating the presence of individual antennae (see spectra 41–48 in Supplementary Fig. 1). Moreover, in the case of all the large structures, the fragmentation spectra did not contain oxonium ions which would suggest the presence of poly-LacNAc extensions of the branches (e.g. compositions Hex_2_HexNAc_2_ or Hex_2_HexNAc_2_NeuNAc_1_). Basing on these observation, we can safely conclude, that in these cases we observed tri- and tetraantennary complex structures rather than diantennary N-glycans with LacNAc repeats. In terms of secreted proteins fraction of control WM266–4-pIRESneo cells, the major peaks within the N-glycan profile did not represent high-mannose species. Seven major peaks were present, with the predominant signals at *m/z* 2198.01 (approx. 31% of all glycans) and *m/z* 2489.19 (approx. 15% of all glycans), which were identified as core fucosylated diantennary complex structures with one (Hex_5_HexNac_4_dHex_1_NeuNAc_1_-AA) and two (Hex_5_HexNAc_4_dHex_1_NeuNAc_2_-2-AA) terminal sialic acids, respectively. The other structures were identified mainly as complex glycans with core fucose, terminal sialic acid residues and a different number of LacNAc units, similarly as in the case of membrane protein fraction. Along with these main peaks, also some weaker signals were detected, both in case of membrane and secreted protein-derived glycans, mainly as fucosylated complex structures with a different number of terminal sialic acids (1–3) and LacNac units (4–6) (Figs. 6 and 7 and Supplementary Table 1). These peaks were present at *m/z* 3510.57, *m/z* 3584.16 and *m/z* 3658.20. Finally, we also observed an unusual core-fucosylated MAN_5_ N-glycan at *m/z* 1500.54 within the membrane protein-derived fraction.

The overexpression of GnT-III in these melanoma cells resulted in the appearance of 29 N-glycans, which were not observed in case of control cells. Most of them were “bisected” structures and were present both within membrane protein-derived N-glycans as well as secreted protein-derived fraction. The presence of the “bisecting” GlcNAc residue was in majority of cases confirmed in fragmentation spectra and the fragment ions at *m/z* 911.4 [M + H]^+^ (H1N3-AA), *m/z* 1056.4 [M + H]^+^ (H1N3Fuc1-AA) and *m/z* 1276.6 [M + H]^+^ (H2N4-AA) were indicative in this case (Supplementary Fig. 1, Supplementary Table 2). The major signal, which is strongly upregulated in the transfectants was observed at *m/z* 2400.7 and identified as diantennary fucosylated complex structure with single terminal sialic acid residue and “bisecting” GlcNAc (Hex_5_HexNAc_5_dHex_1_NeuNAc_1_-2-AA) (Supplementary Table 1, Figs. 6 and 7). The relative abundance of this oligosaccharide is approximately 15% of membrane-derived N-glycans and about 23% for the secreted protein-derived fraction. Other major peaks were identified as “bisected” diantennary glycans with or without core fucose and with different length of the antennae. Interestingly, the addition of the “bisecting” GlcNAc as a result of GnT-III overexpression also occurred in case of hybrid glycans, and 4 different species were identified at *m/z* 1922.5, *m/z* 2068.6, *m/z* 2213.6 and *m/z* 2359.7 (Hex_6_HexNAc_4_ with or without sialic acid, and with or without core fucose). Finally, the addition of “bisecting” *N*-acetylglucosamine in WM266–4-GnT-III cells involved also N-glycans containing more than two LacNAc repeats. The major peaks within this group of oligosaccharides were detected at *m/z* 2474.7 (Hex_6_HexNAc_6_dHex_1_-AA), *m/z* 2765.8 (Hex_6_HexNAc_6_dHex_1_NeuNAc_1_-AA), *m/z* 3056.9 (Hex_6_HexNAc_6_dHex_1_NeuNAc_2_-AA), *m/z* 3130.9 (Hex_7_HexNAc_7_dHex_1_NeuNAc_1_-AA) and *m/z* 3422.0 (Hex_7_HexNAc_7_dHex_1_NeuNAc_2_-AA). These can be identified as “bisected” tri- or tetraantennary complex structures from the similar reason as in case of control cells and all of them were characteristic for both membrane and secreted protein-derived glycans. Interestingly, in both cases we also identified trisialylated structures: triantennary “bisected” glycan at *m/z* 3348.2 (Hex_6_HexNAc_6_dHex_1_NeuNAc_3_) and tri- or tetraantennary “bisected” structure at *m/z* 3713.2 (Hex_7_HexNAc_7_dHex_1_NeuNAc_3_-2-AA) among secreted protein-derived glycans. Some other of the identified N-glycans were present only in case of one of the protein fractions. For example, an interesting “bisected” glycan at *m/z* 2645.8 (Hex_4_HexNAc_7_dHex_3_-AA), were found only in case of the secreted protein fraction (Supplementary Table 1). By contrast, two highly branched complex structures with poly-LacNAc extensions and “bisecting” GlcNAc were found at *m/z* 3787.1 (Hex_8_HexNAc_8_dHex_1_NeuNAc_2_-AA) and *m/z* 3861.2 (Hex_9_HexNAc_9_dHex_1_NeuNAc_1_-AA) only in case of the membrane protein-derived N-glycans (Supplementary Table 1).

Finally, we also observed peaks satellite to some sialylated complex N-glycans and basing on the mass shift of 18 Da we identified them as structures containing lactonized *N*-acetylneuraminic acid residues (NeuNAcLac) (Figs. 6 and 7 and Supplementary Table 1). As the internal esterification concerns α2-3-linked sialic acid, the glycans at *m/z* 2197.6 (Hex_5_HexNAc_4_dHex1NeuNAc_1_-2-AA), *m/z* 2400.7 (Hex_5_HexNAc_5_dHex_1_NeuNAc_1_-2-AA), *m/z* 2488.8 (Hex_5_HexNAc_4_dHex_1_NeuNAc-2-AA), *m/z* 2691.8 (Hex_5_HexNAc_5_dHex_1_NeuNAc_2_-2-AA), *m/z* 2853.9 (Hex_6_HexNAc_5_dHex_1_NeuNAc-2-AA) and *m/z* 3056.9 (Hex_6_HexNAc_6_dHex_1_NeuNAc_2_-2-AA) were identified as containing at least one terminal α2-3-linked NeuNAc.

## Discussion

*N-*Acetylglucosaminyltransferase III (GnT-III) catalyzes the synthesis of “bisected” N-glycan structures by the transfer of a single GlcNAc residue to the β-mannose of the core. This reaction is one of the critical steps of the glycosylation pathway, and the resulting “bisecting” GlcNAc has unique features among all other N-glycans modification. First of all, in contrast to other GlcNAc branches, it is not further elongated by other transferases [6]. Secondly, in terms of the topography of glycosylation machinery within Golgi apparatus, the action of GnT-III is very often considered as a “stop” signal because the presence of its product prevents the action of other GlcNAc transferases such as GnT-II, IV and V but also core α1-6 fucosyltransferase and α mannosidase II [39]. This gives the enzyme the potency to be a kind of a “switcher”, which through the changes of its expression level and the activity, could regulate the formation of complex N-glycan structures of the cellular glycoproteins. Thus, the biological role of GnT-III is under intensive investigation, partially in terms of its impact on cancer cell behavior.

Previously, we described the melanoma cellular model, in which WM266–4 metastatic melanoma cells were stably transfected with *MAGT3* gene [28]. Although the upregulation of GnT-III is functional because it has resulted in the modification of Melanoma Cell Adhesion Molecule (MCAM) glycans by introducing “bisecting” GlcNAc residue, it has not significantly influenced the transendothelial invasiveness of the cells in vitro. The WM266–4 melanoma cell line was chosen as a transfection host partially because it was described as expressing a high level of highly branched oligosaccharides (tri- and tetraantennary N-glycans) [40]. The aim of the present study was to characterize in detail the influence of GnT-III overexpression on the N-glycan repertoire of membrane and secreted proteins in these metastatic melanoma cells. We used WM266–4-pIRESneo cells as a transfection control and WM266–4-GnT-III cell line as a model of GnT-III overexpression [28].

The stronger reaction of glycoproteins with PHA-E upon GnT-III overexpression is a logical consequence of the enzyme upregulation and is usually treated as a positive control of successful transfection of cells [7, 8, 41, 42]. Surprisingly, we did observe also stronger staining with PHA-L, which would suggest that GnT-III up-regulation led also to the elevation of β1–6 branching of complex N-glycan structures. This remains in contrast to the fact, that action of GnT-III is considered as inhibitory towards GnT-V enzyme, which was observed by weakened PHA-L staining in many cases of *MGAT3* transfectants [7, 13, 43]. To our knowledge, this report together with our previous studies [28] are the only descriptions of elevated PHA-L staining of glycoproteins upon GnT-III overexpression. The one explanation for this phenomenon is the possible influence of GnT-III on the GnT-V activity or its expression level. However, previously we showed no impact of *MGAT3* transfection either on the expression of GnT-V nor its activity in vitro [28]. The other possible explanation is that the introduction of “bisecting” GlcNAc residues causes more global glycomic effects, which in turns changes the binding interactions of some previously existing N-glycans structures with PHA-L. To investigate this effect, we performed detailed glycomic analysis of N-glycans isolated from cellular glycoproteins of studied cells.

The elevated presence of “bisected” biantennary glycans, which we observed in case of GnT-III overexpressing WM266–4-GnT-III cells, has been often described as the result of GnT-III overexpression, but there are not many detailed descriptions of glycomic changes in these cases. Ihara et al. showed the appearance of diantennary structure with “bisecting” GlcNAc residue on serum glycoproteins in GnT-III transgenic mouse [41]. Koyota et al. presented the upregulation of “bisected” diantennery oligosaccharides in swine endothelial cells overexpressing GnT-III with the simultaneous down regulation of tri- and tetraantennary sugars [44]. In terms of cancer biology, the biological function of such modifications of cellular glycome has been described mainly as modulator of cellular migration and invasiveness. In the light of these data, GnT-III indeed can act as a metastasis inhibitor, but in the majority of published cases, this effect is linked to the simultaneous, almost total down-regulation of N-glycan branching as a result of competition between GnT-III and GnT-V for an acceptor substrate.

Interestingly, our data show that besides the diantennary glycans, into which the “bisecting” GlcNAc is introduced upon GnT-III overexpression, other types of glycans are modified in this manner as well. In case of branched “bisected” structures, one triantennary and one at least triantennary sialylated complex glycans were identified at *m/z* 3348.2 (Hex_6_HexNAc_6_dHex_1_NeuNAc_3_) and *m/z* 3713.2 (Hex_7_HexNAc_7_dHex_1_NeuNAc_3_-2-AA), respectively. Adding to that, a broad group of “bisected” N-glycans containing LacNAc units were annotated, starting from simple core fucosylated and non-sialylated structure at *m/z* 2474.8 (Hex_6_HexNAc_6_dHex_1_-2-AA) up to mono sialylated oligosaccharide containing six LacNAc units at *m/z* 3861.2 (Hex_9_NeuNAc_9_dHex_1_NeuNAc_1_-2-AA). Basing on mass analysis and fragmentation data it was not possible here to make a clear distinction between highly branched structures and diantennary glycans with poly-LacNAc extensions. However, as the LC-ESI-ion trap-MS data indicates a high level of sialylation and the fragmentation spectra do not give an evidence for the presence of LacNAc repeats in studied samples, it is more likely that the multiantennary N-glycans prevail. Adding to that, Kinoshita et al. have described a glycome of WM266–4 melanoma cells which we used as a transfection host, as containing a high amount of tri- and tetra-antennary complex N-glycans [45]. Basing on mass analysis, our results concerning the glycosylation of control cells (WM266–4-pIRESneo) are in line with these data. In case of the transfectants (WM266–4-GnT-III cells), we presume that these highly branched N-glycans were also modified by GnT-III. It is evidently visible when comparing the MALDI-TOF spectra of control and transfected cells, while there is a significant shift of main peaks (besides high mannose glycans) of the constant value of *m/z* 203, which corresponds to a single GlcNAc residue (Figs. 6 and 7).

The presence of “bisecting” GlcNAc in the tri- and tetra-antennary glycans is very unusual, because of the ability of this modification to block the activity of other GlcNAc transferases, which was mentioned above [46]. A few years ago Klisch et al. described the high content of tri- and tetra-antennary, core fucosylated N-glycans containing “bisected” GlcNAc on pregnancy-associated glycoproteins (PAGs) in ruminants [47]. The occurrence of such structures could be the result of the specific spatial distribution of glycosyltransferases within the Golgi compartments. The introduction of single GlcNAc residue to the β-mannose of the core of highly branched structures would be enabled by the localization of GnT-IV and -V earlier within the secretory pathway than GnT-III. Interestingly, it was proved, that caveolin-1 by forming complexes with GnT-III can act as a regulator of its distribution within the Golgi [48]. The expression of caveolin-1 in melanoma cells has been studied by *Bełkot* et al., and their work showed that in case of WM266–4 melanoma cells its expression is relatively low and the distribution within the cells do not show localization of the protein in ER and Golgi *apparatus* [49]. It is possible, that in WM266–4-GnT-III melanoma cells studied here, the level of caveolin-1 is thus too low to localize GnT-III in the early compartments of the secretory pathway. From the other hand, we observe the presence of hybrid, “bisected” glycans, which formation probably occurs within the early Golgi and could be the effect of α mannosidase II inhibition by GnT-III product [50].

In case of melanoma model described here, GnT-III action seems to involve almost all hybrid and complex N-glycans regardless of their molecular mass, leading to a broad glycomic effect. But, the mechanisms and biological consequences of such modification remains challenging to study in detail, partially because the mutual interplay of glycosyltransferases within secretory pathway is still a puzzle. First of all, an ambiguous picture of the role of “bisected” N-glycans in cancer allows to raise the assumption, that the cellular context (for example overall levels of different branching glycosyltransferases) may contribute to determining structure and function of “bisected” glycans [51]. There is also growing number of evidence that glycosyltransferases can form homomers and heteromers, and the balance of the formation of these species can influence the activity of the enzymes itself [52]. The elevated reactivity of cellular glycoproteins with PHA-L that we observe here is for sure the consequence of elevated GnT-III action in the cells. It is known, that the introduction of “bisecting” GlcNAc residue changes the spatial shape of N-glycans and thus can modulate its biological properties [4]. A single sugar substitution of the N-glycan core is especially studied in this context, giving evidence that “bisecting” GlcNAc and core fucose can be treated as molecular switches of conformational behaviour of glycan structures [53]. Recently, it has been shown, that “bisecting” GlcNAc can restrict the conformers of branched structures by the formation of the ‘back-fold’ conformation [54]. As a consequence, the glycan binding to specific lectin can be changed. It is possible that the stronger binding of glycoproteins from WM266–4-GnT-III cells to the PHA-L is the result of a conformational change made by the introduction of “bisecting” GlcNAc into highly branched structures, which leads to the promotion of PHA-L binding. It cannot be excluded, that similar modifying effect as a result of GnT-III overexpression would be observed in melanoma cells *in vivo*, if other carbohydrate-protein interactions were taken into account. In case of branched glycans the studies concerning their interactions with mammalian lectins are however limited [55]. But, the potential of “bisecting” GlcNAc to be a modificator of these interactions seems to be worth to consider, as we know, for example, that mouse dendritic cell inhibitory receptor 2 (mDCIR2) recognizes not only the α1–3 branch but also a “bisecting” GlcNAc residue of diantennary complex glycans [56].

To conclude, our studies provide the detailed glycomic data of the effect of GnT-III overexpression in melanoma cells. We showed that the enzyme can modify highly-branched N-glycans by the introduction of the “bisecting” GlcNAc residue. It probably strengthens the binding capacity of some of these structures to PHA-L, which is the phenomenon that has not been described so far. Our results suggest the need for further studies of PHA-L binding specificity towards different types of complex N-glycans. Moreover, in our opinion, the glycomic effect seen here could lead to the changes of other protein-carbohydrate interactions, also *in vivo*. Thus, it would be important to study the biological impact of introducing of the “bisecting” GlcNAc residue into complex N-glycans on their biological function, for example, specificity toward glycan-binding proteins, especially in the context of cancer cell biology.

## Electronic supplementary material

Supplementary Table 1 (PDF 96 kb)

Supplementary Table 2 (PDF 260 kb)

Supplementary Figure 1 (PDF 1991 kb)

Supplementary Figure 2 (PDF 178 kb)

## Abbreviations

AA: 2-aminobenzoic acid
Fuc, F: fucose
Hex, H: hexose
HILIC: hydrophilic interaction liquid chromatography
HexNAc, N: *N*-acetylhexosamine
mDCIR2: mouse dendritic cell inhibitory receptor 2
NeuAc, SA: *N*-acetylneuraminic acid
NeuNAcLac: lactonized *N*-acetylneuraminic acid
PAG: pregnancy-associated glycoprotein
