# Supplementary material for: The glycomic effect of N-acetylglucosaminyltransferase III overexpression in metastatic melanoma cells. GnT-III modifies highly branched N-glycans
Source: Glycoconj J. 2018 Mar 3;35(2):217–31. doi: 10.1007/s10719-018-9814-y (PMC5916991; doi:10.1007/s10719-018-9814-y)
Supplement: Supplementary file 1 — (PDF 96 kb) [file 10719_2018_9814_MOESM1_ESM.pdf]

Supplementary Table 1. Characterization of N-glycans present in WM266-4-pIRESneo and WM266-4-GnT-III cells. A part of the 2-AA-labeled N-glycans released from membrane and secreted proteins extracted from both cell lines were analyzed by negative-mode MALDI-ToF-MS. In order to confirm the composition of the N-glycans the samples were further analyzed by ESI-ion trap-MS/MS. ESI-MS/MS fragmentations were used for structural assignment together with common knowledge of glycobiology. The fragmentation spectra represented in Supplementary Figure 1 and Supplementary Table 2 are listed in the last column. The relative percentage intensities of each peak were given in the table. The intensities are calculated basing on the analyses of the samples in the MALDI-TOF linear mode, wheras the *m/z* values are from reflectron mode. The symbols used for monosaccharides are: Hex, hexose; HexNAc, N-acetylhexosamine; dHex, fucose; NeuNAc, N-acetylneuraminic acid; NeuNAcLac - lactonized N-acetylneuraminic acid; S, sulfate group.

	MALDI-ToF-MS WM266-4-pIRESneo membrane			MALDI-ToF-MS WM266-4-GnT-III membrane			MALDI-ToF-MS WM266-4-pIRESneo secreted			MALDI-ToF-MS WM266-4-GnT-III secreted			Theoretical *m/z* [M-H]⁻	Composition	ESI-IT MS/MS
glycan ID	***m/z***	Intens.	% intensity	***m/z***	Intens.	% intensity	***m/z***	Intens.	% intensity	***m/z***	Intens.	% intensity			
1	**1014.31**	2045	1.48	**1014.31**	2320	1.48	**1014.45**	533	0.41				**1014.38**	Hex2HexNAc2dHex1-AA	
2	**1030.31**	1177	0.85	**1030.3**	1252	0.80	**1030.49**	383	0.29				**1030.37**	Hex3HexNAc2-AA	**Spectrum 1**
3	**1176.36**	3802	2.75	**1176.34**	3678	2.34	**1176.53**	723	0.55	**1176.34**	628	0.41	**1176.43**	Hex3HexNAc2dHex1-AA	**Spectrum 2**
4	**1192.35**	961	0.69	**1192.34**	1053	0.67	**1192.51**	397	0.30				**1192.43**	Hex4HexNAc2-AA	
5	**1354.39**	4738	3.42	**1354.37**	4356	2.77	**1354.59**	3770	2.89	**1354.31**	1279	0.83	**1354.48**	Hex5HexNAc2-AA	**Spectrum 3**
6	**1379.39**	1289	0.93	**1379.38**	991	0.63	**1379.67**	430	0.33				**1379.51**	Hex3HexNAc3dHex1-AA	**Spectrum 4**
7	**1395.43**	812	0.59	**1395.4**	994	0.63	**1395.61**	394	0.30				**1395.50**	Hex4HexNAc3-AA	**Spectrum 5**
8	**1500.54**	704	0.51	**1500.49**	772	0.49							**1500.54**	Hex5HexNAc2dHex1-AA	**Spectrum 6**
9	**1516.45**	26187	18.91	**1516.41**	17159	10.92	**1516.66**	4252	3.26	**1516.35**	1545	1.00	**1516.53**	Hex6HexNAc2-AA	**Spectrum 7**
10	**1541.5**	1132	0.82	**1541.43**	1500	0.95	**1541.69**	794	0.61	**1541.43**	403	0.26	**1541.56**	Hex4HexNAc3dHex1-AA	**Spectrum 8**
11	**1582.56**	1307	0.94	**1582.47**	2071	1.32				**1582.42**	974	0.63	**1582.59**	Hex3HexNAc4dHex1-AA	**Spectrum 9**
12	**1598.93**	544	0.39	**1598.93**	1077	0.69	**1598.74**	535	0.41	**1598.4**	754	0.49	**1598.58**	Hex4HexNAc4-AA	**Spectrum 10**
13			0.00			0.00				**1639.44**	2221	1.44	**1639.61**	Hex3HexNAc5-AA	**Spectrum 11**
14			0.00			0.00	**1670.74**	608	0.47				**1670.61**	Hex3HexNAc3dHex1NeuNAc1-AA	
15	**1678.55**	14711	10.63	**1678.46**	9048	5.76	**1678.72**	2089	1.60	**1678.42**	803	0.52	**1678.58**	Hex7HexNAc2-AA	**Spectrum 12**
16	**1686.56**	1008	0.73	**1686.52**	760	0.48	**1686.76**	711	0.54				**1686.60**	Hex4HexNAc3NeuNAc1-AA	**Spectrum 13**
17			0.00	**1744.54**	1769	1.13	**1744.78**	863	0.66	**1744.47**	1565	1.01	**1744.64**	Hex4HexNAc4dHex1-AA	**Spectrum 14**
19	**1760.63**	785	0.57	**1760.49**	2673	1.70	**1760.77**	982	0.75	**1760.45**	2286	1.48	**1760.64**	Hex5HexNAc4-AA	**Spectrum 15**
20	**1785.66**	478	0.35	**1785.54**	2064	1.31			0.00	**1785.49**	5673	3.67	**1785.67**	Hex3HexNAc5dHex1-AA	**Spectrum 16**
22			0.00	**1801.52**	1557	0.99			0.00	**1801.48**	3366	2.18	**1801.66**	Hex4HexNAc5-AA	**Spectrum 17**
23	**1832.63**	2162	1.56	**1832.54**	1316	0.84	**1832.82**	1685	1.29				**1832.66**	Hex4HexNAc3dHex1NeuNAc1-AA	**Spectrum 18**
24	**1840.6**	12280	8.87	**1840.51**	7072	4.50	**1840.79**	1236	0.95	**1840.46**	664	0.43	**1840.63**	Hex8HexNAc2-AA	
25	**1848.64**	862	0.62			0.00	**1848.79**	775	0.59				**1848.65**	Hex5HexNAc3NeuNAc1-AA	**Spectrum 19**
26			0.00			0.00	**1889.84**	717	0.55	**1889.46**	445	0.29	**1889.68**	Hex4HexNAc4NeuNAc1-AA	
27	**1906.63**	1244	0.90	**1906.56**	4411	2.81	**1906.87**	4145	3.18	**1906.49**	2723	1.76	**1906.69**	Hex5HexNAc4dHex1-AA	**Spectrum 20**
29			0.00	**1922.56**	2047	1.30			0.00	**1922.49**	1065	0.69	**1922.69**	Hex6HexNAc4-AA	**Spectrum 21**
30			0.00	**1947.59**	3212	2.04	**1947.92**	403	0.31	**1947.53**	19896	12.86	**1947.72**	Hex4HexNAc5dHex1-AA	**Spectrum 22**
31			0.00	**1963.59**	2214	1.41			0.00	**1963.5**	1343	0.87	**1963.71**	Hex5HexNAc5-AA	**Spectrum 23**
32	**1994.66**	1119	0.81	**1994.55**	478	0.30	**1994.89**	462	0.35				**1994.71**	Hex5HexNAc3dHex1NeuNAc1-AA	**Spectrum 24**
33	**2002.64**	17453	12.61	**2002.56**	8797	5.60	**2002.91**	766	0.59				**2002.69**	Hex9HexNAc2-AA	
34	**2010.64**	995	0.72	**2010.6**	606	0.39	**2010.88**	695	0.53				**2010.71**	Hex6HexNAc3NeuNac1-AA	**Spectrum 25**
35	**2035.7**	722	0.52	**2035.62**	941	0.60	**2035.91**	1659	1.27	**2035.54**	1331	0.86	**2035.73**	Hex4HexNAc4dHex1NeuNAc1-AA	**Spectrum 26**
36	**2051.69**	3534	2.55	**2051.59**	712	0.45	**2051.92**	6651	5.10	**2051.5**	786	0.51	**2051.73**	Hex5HexNAc4NeuNAc1-AA	**Spectrum 27**
37			0.00	**2068.62**	1874	1.19			0.00	**2068.58**	955	0.62	**2068.75**	Hex6HexNAc4dHex1-AA	**Spectrum 28**
39			0.00	**2092.62**	694	0.44			0.00	**2092.53**	1296	0.84	**2092.75**	Hex4HexNAc5NeuNAc1-AA	
40			0.00	**2109.63**	8020	5.10			0.00	**2109.56**	9790	6.33	**2109.77**	Hex5HexNAc5dHex1-AA	**Spectrum 29**
41	**2156.7**	610	0.44			0.00			0.00				**2156.74**	Hex6HexNAc3dHex1NeuNAc1-AA	**Spectrum 30**

42	**2164.72**	653	0.47	**2164.59**	695	0.44			0.00				**2164.74**	Hex10HexNAc2-AA	
43			0.00			0.00	**2179.93**	1303	1.00				**2179.78**	Hex5HexNac4dHex1NeuNAcLac1-AA	
44	**2197.76**	11984	8.66	**2197.66**	1833	1.17	**2198.01**	41182	31.57	**2197.58**	2229	1.44	**2197.79**	Hex5HexNac4dHex1NeuNAc1-AA	**Spectrum 31**
45			0.00	**2213.63**	636	0.40			0.00	**2213.57**	722	0.47	**2213.78**	Hex6HexNAc4NeuNAc1-AA	**Spectrum 32**
46			0.00	**2238.67**	2414	1.54	**2239.03**	2328	1.78	**2238.6**	8931	5.77	**2238.81**	Hex4HexNAc5dHex1NeuNAc1-AA	**Spectrum 33**
48			0.00	**2254.67**	3748	2.38			0.00	**2254.6**	3305	2.14	**2254.81**	Hex5HexNAc5NeuNAc1-AA	**Spectrum 34**
49			0.00			0.00	**2272.05**	775	0.59			0.00	**2271.83**	Hex6HexNAc5dHex1-AA	
50			0.00			0.00	**2280.1**	722	0.55			0.00	**2279.84**	Hex3HexNAc6dHex1NeuNAc1-AA	
51			0.00			0.00			0.00	**2312.69**	916	0.59	**2312.85**	Hex5HexNAc6dHex1-AA	
52	**2342.8**	1198	0.87			0.00	**2343.1**	2281	1.75			0.00	**2342.83**	Hex5HexNAc4NeuNAc2-AA	**Spectrum 35**
53			0.00	**2359.7**	1060	0.67			0.00	**2359.62**	1037	0.67	**2359.84**	Hex6HexNAc4dHex1NeuNAc1-AA	**Spectrum 36**
54			0.00			0.00				**2382.59**	1643	1.06	**2382.86**	Hex5HexNac5dHex1NeuNAcLac1-AA	
55	**2400.86**	347	0.25	**2400.73**	24845	15.81	**2401.11**	462	0.35	**2400.65**	36097	23.33	**2400.87**	Hex5HexNac5dHex1NeuNAc1-AA	**Spectrum 37**
56	**2416.83**	781	0.56	**2416.71**	742	0.47	**2417.15**	900	0.69			0.00	**2416.86**	Hex6HexNAc5NeuNAc1-AA	
57			0.00			0.00	**2471.13**	7557	5.79			0.00	**2470.87**	Hex5HexNAc4dHex1NeuNAc1NeuNAcLac1-AA	
58							**2443.12**	372	0.29				**2442.92**	Hex4HexNAc6dHex3-AA	
59			0.00	**2474.8**	1512	0.96			0.00	**2474.67**	1057	0.68	**2474.91**	Hex6HexNAc6dHex1-AA	
60	**2488.92**	6389	4.61			0.00	**2489.19**	20037	15.36	**2488.77**	832	0.54	**2488.89**	Hex5HexNAc4dHex1NeuNac2-AA	**Spectrum 38**
61			0.00	**2545.77**	744	0.47			0.00	**2545.69**	1061	0.69	**2545.91**	Hex5HexNAc5NeuNAc2-AA	**Spectrum 39**
62	**2562.88**	3074	2.22	**2562.78**	1282	0.82	**2563.17**	4673	3.58	**2562.72**	1018	0.66	**2562.92**	Hex6HexNAc5dHex1NeuNAc1-AA	
63			0.00	**2603.84**	918	0.58			0.00	**2603.71**	1718	1.11	**2603.95**	Hex5HexNAc6dHex1NeuNAc1-AA	
64			0.00	**2619.76**	821	0.52			0.00			0.00	**2619.94**	Hex6HexNAc6NeuNAc1	
66			0.00			0.00			0.00	**2673.75**	5246	3.39	**2673.95**	Hex5HexNAc5dHex1NeuNAc1NeuNAcLac1-AA	
67			0.00	**2691.85**	4888	3.11			0.00	**2691.76**	15880	10.26	**2691.96**	Hex5HexNAc5dHex1NeuNAc2-AA	**Spectrum 40**
68	**2708.04**	551	0.40			0.00			0.00			0.00	**2707.96**	Hex6HexNAc5NeuNAc2-AA	
69			0.00	**2765.84**	4819	3.07			0.00	**2765.79**	3571	2.31	**2766.00**	Hex6HexNAc6dHex1NeuNAc1-AA	**Spectrum 41**
70	**2781.96**	463	0.33			0.00			0.00			0.00	**2782.00**	Hex7HexNAc6NeuNAc1-AA	
71	**2835.88**	1039	0.75				**2836.30**	1693	1.30				**2836.01**	Hex6HexNAc5dHex1NeuNAc1NeuNAcLac1-AA	
72			0.00	**2839.83**	694	0.44			0.00	**2839.80**	586	0.38	**2840.04**	Hex7HexNAc7dHex1-AA	
73	**2854.07**	2892	2.09	**2853.89**	531	0.34	**2854.37**	4244	3.25	**2853.81**	544	0.35	**2854.02**	Hex6HexNAc5dHex1NeuNAc2-AA	**Spectrum 42**
74	**2927.98**	1724	1.25	**2927.92**	527	0.34	**2928.35**	1271	0.97			0.00	**2928.05**	Hex7HexNAc6dHex1NeuNAc1-AA	**Spectrum 43**
75			0.00	**2968.93**	455	0.29			0.00	**2968.8**	483	0.31	**2969.08**	Hex6HexNAc7dHex1NeuNAc1-AA	
76										**3038.74**	1029	0.67	**3039.09**	Hex6HexNAc6dHex1NeuNAc1NeuNAcLac1-AA	
77			0.00	**3056.95**	1632	1.04			0.00	**3056.85**	2268	1.47	**3057.10**	Hex6HexNAc6dHex1NeuNAc2-AA	**Spectrum 44**
78			0.00	**3130.93**	2119	1.35			0.00	**3130.86**	1298	0.84	**3131.13**	Hex7HexNAc7dHex1NeuNAc1-AA	**Spectrum 45**
79	**3145.19**	1170	0.85			0.00	**3145.55**	1715	1.31			0.00	**3145.11**	Hex6HexNAc5dHex1NeuNAc3-AA	**Spectrum 46**
80	**3219.15**	1499	1.08			0.00	**3219.55**	1459	1.12			0.00	**3219.15**	Hex7HexNAc6dHex1NeuNAc2-AA	**Spectrum 47**
81	**3293.05**	469	0.34			0.00						0.00	**3293.19**	Hex8HexNAc7dHex1NeuNAc1-AA	
82				**3348.14**	334	0.21				**3348.22**	372	0.24	**3348.19**	Hex6HexNAc6dHex1NeuNAc3	
83			0.00	**3422.04**	1096	0.70			0.00	**3421.97**	820	0.53	**3422.23**	Hex7HexNAc7dHex1NeuNAc2-AA	
84			0.00	**3496.06**	677	0.43			0.00	**3496.06**	163	0.11	**3496.26**	Hex8HexNAc8dHex1NeuNAc1-AA	
85	**3510.38**	792	0.57			0.00	**3510.57**	829	0.64			0.00	**3510.25**	Hex7HexNAc6dHex1NeuNAc3-AA	**Spectrum 48**
86	**3584.16**	429	0.31			0.00						0.00	**3584.28**	Hex8HexNAc7dHex1NeuNAc2-AA	
87	**3658.2**	339	0.24			0.00						0.00	**3658.32**	Hex9HexNAc8dHex1NeuNAc1-AA	
88						0.00				**3713.21**	118	0.08	**3713.32**	Hex7HexNAc7dHex1NeuNAc3-AA	
89				**3787.07**	435	0.28							**3787.36**	Hex8HexNAc8dHex1NeuNAc2-AA	
90				**3861.21**	249	0.16							**3861.40**	Hex9HexNAc9dHex1NeuNAc1-AA	
		**138454**	**100.00**		**157194**	**100.00**		**130461**	**100.00**		**154735**	**100.00**			
