# Supplementary material for: The glycomic effect of N-acetylglucosaminyltransferase III overexpression in metastatic melanoma cells. GnT-III modifies highly branched N-glycans
Source: Glycoconj J. 2018 Mar 3;35(2):217–31. doi: 10.1007/s10719-018-9814-y (PMC5916991; doi:10.1007/s10719-018-9814-y)
Supplement: Supplementary file 2 — (PDF 260 kb) [file 10719_2018_9814_MOESM2_ESM.pdf]

Supplementary Table 2. Characterization of N-glycans present in WM266-4-pIRESneo and WM266-4-GnT-III cells.
A part of the 2-AA-labeled N-glycans released from membrane and secreted proteins extracted from both cell lines were analyzed by positive-mode ESI-ion trap-MS/MS. Glycans fragmentations were used for structural assignment together with common knowledge of glycobiology. The first column gives the MS/MS spectrum number and refers to Supplementary Figure 1. The second column gives the N-glycan *m/z* values measured by positive-mode ESI-ion trap-MS. The values in the third column correspond to the theoretical *m/z* of the N-glycans and the fourth column contains the glycan composition. The fragments of the ESI-MS/MS analysis ([M+H]+ and [M+2H]2+) are given in the fifth column. These fragments are visualized in Supplementary Figure 1. Next to signal intensity, other relevant criteria used for peak picking were the observation of a proper isotope distribution, and mass accuracy. The symbols used for monosaccharides are: Hex, H, hexose; HexNAc, N, N-acetylhexosamine; dHex, Fuc, F, fucose; NeuNAc, SA, N-acetylneuraminic acid.

**Spectrum**	**ESI-IT-MS** *m/z*	**Theoretical** *m/z*	**Composition**	**Fragments in ESI-ion trap-MS/MS (positive mode)**
**Spectrum1**	1032.43 +	1032. 39 +	Hex3HexNAc2-AA	**[M+H]+:** 342.95 (N1-AA); 365.94 (H1N1); 528.06 (H2N1); 546.09 (N2-AA); 690.20 (H3N1); 708.25 (H1N2-AA); 870.27 (H2N2-AA)
**Spectrum 2**	589.76 ++	589.72 ++	Hex3HexNAc2Fuc1-AA	**[M+H]+:** 203.87 (N1); 273.46^{2+} (N2-AA); 342.93 (N1-AA); 346.50^{2+} (N2Fuc1-AA); 354.47^{2+} (H1N2-AA); 365.94 (H1N1); 489.12 (N1Fuc1-AA); 435.65^{2+} (H2N2-AA); 546.12 (N2-AA); 692.29 (N2Fuc1-AA); 708.28 (H1N2-AA); 854.38 (H1N2Fuc1-AA); 870.33 (H2N2-AA)
**Spectrum 3**	678,81 ++	678,75 ++	Hex5HexNAc2-AA	**[M+H]+**: 203.91 (N1); 273.37^{2+} (N2-AA); 324.95 (H2); 342.91 (N1-AA); 365.91 (H1N1); 435.91^{2+} (H2N2-AA); 528.07 (H2N1); 546.12 (N2-AA); 598.13^{2+} (H4N2-AA) 708.20 (H1N2-AA); 870.31 (H4N1-AA)
**Spectrum 4**	691.28 ++	691.27 ++	Hex3HexNAc3Fuc1-AA	**[M+H]+**: 203.89 (N1); 342.91 (N1-AA); 365.94 (H1N1); 435.91^{2+} (H2N2-AA); 489.05 (N1Fuc1-AA); 528.04 (H2N1); 546.19 (N2-AA); 1016.37 (H2N2Fuc1-AA); 1178.41 (H3N2F1-AA)
**Spectrum 5**	699.31 ++	699.26 ++	Hex4HexNAc3-AA	**[M+H]+**: 203.90 (N1); 343.00 (N1-AA); 365.89 (H1N1); 528.04 (H2N1); 708.14 (H1N2-AA); 870.25 (H4N1); 1032.43 (H3N2-AA); 1194.05 (H4N2-AA); 1235.65 (H3N3-AA)
**Spectrum 6**	751.80 ++	751.78 ++	Hex5HexNAc2Fuc1-AA	**[M+H]+:** 273.932+ (N2-AA); 342.93 (N1-AA); 354.472+ (H1N2-AA); 365.91 (H1N1); 427.972+ (H1N2Fuc1-AA); 435.462+ (H2N2-AA); 489.08 (N1Fuc1-AA); 546.10 (N2-AA); 690.20 (H3N1); 692.17 (N2Fuc1-AA); 708.28 (H1N2-AA); 834.07 (H4N1); 854.23 (H1N2Fuc1-AA); 1014.48 (H3N2-AA)
**Spectrum 7**	759,82 ++	759,78 ++	Hex6HexNAc2-AA	**[M+H]+:** 273.442+ (N2-AA); 324.92 (H2); 342.95 (N1-AA); 365.92 (H1N1); 435.542+ (H2N2-AA); 516.60 (H5N1); 528.06 (H2N1); 546.12 (N2-AA); 597.672+ (H4N2-AA); 690.13 (H3N1); 708.25 (H1N2-AA); 852.26 (H4N1); 870.30 (H2N2-AA); 1032.33 (H3N2-AA); 1194.47 (H4N2-AA)
**Spectrum 8**	772.34 ++	772.29 ++	Hex4HexNAc3Fuc1-AA	**[M+H]+:** 365.94 (H1N1); 528.20 (H2N1); 691.15^{2+} (H3N3Fuc1-AA); 854.40 (H1N2Fuc1-AA); 1016.34 (H2N2Fuc1-AA); 1178.40 (H3N2Fuc1-AA)
**Spectrum 9**	792.84 ++	792.80 ++	Hex3HexNAc4Fuc1-AA	**[M+H]+:** 365.89 (H1N1); 528.07^{2+} (H2N3-AA); 708.06 (H1N2-AA); 893.29 (H3N2); 1016.38 (H2N2Fuc1-AA); 1178.46 (H3N2Fuc1-AA); 1382.52 (H3N3Fuc1-AA)

**Spectrum 10**	800.91 ++	800.80++	Hex4HexNAc4-AA	**[M+H]+:** 365.93 (H1N1); 456.05^{2+} (H1N3-AA); 893.28 (H3N2); 911.20 (H1N3-AA); 1032.40 (H3N2-AA); 1073.43 (H2N3-AA); 1235.47 (H3N3-AA); 1397.41 (H4N3-AA)
**Spectrum 11**	821,36 ++	821,32 ++	Hex3HexNAc5-AA	**[M+H]+**: 365.95 (H1N1); 569.07 (H1N2); 731.22 (H2N2); 893.27 (H3N2); 911.32 (H1N3-AA); 1073.38 (H2N3-AA); 1235.46 (H3N3-AA); 1299.47 (H3N4); 1438.52 (H3N4-AA)
**Spectrum 12**	840.86 ++	840.80 ++	Hex7HexNAc2-AA	**[M+H]+:** 343.09 (N1-AA); 505.12 (H3); 546.09 (N2-AA); 595.24 (H4); 690.08 (H3N1); 708.24 (H1N2-AA); 870.28 (H2N2-AA); 1032.06 (H3N2-AA)
**Spectrum 13**	844.89 ++	844.81 ++	Hex4HexNAc3NeuNAc1-AA	**[M+H]+:** 365.95 (H1N1); 528.08 (H2N1); 657.21 (H1N1SA1); 690.24 (H3N1); 819.23 (H2N1SA1); 870.27 (H2N2-AA); 1032.33 (H3N2-AA); 1397.42 (H4N3-AA)
**Spectrum 14**	873.87 ++	873.83 ++	Hex4HexNAc4Fuc1-AA	**[M+H]+:** 365.95 (H1N1); 489.01 (N1F1); 708.24 (H1N2-AA); 792.68^{2+} (H3N4F1-AA); 893.30 (H3N2); 911.40 (H1N3); 1057.40 (H1N3F1-AA); 1219.43 (H2N3F1-AA); 1381.51 (H3N3F1-AA); 1543.53 (H4N3F1-AA)
**Spectrum 15**	881.89 ++	881.83 ++	Hex5HexNAc4-AA	**[M+H]+:** 365.93 (H1N1); 528.08 (H2N1); 911.40 (H1N3-AA); 1055.41 (H2N3-AA); 1235.44 (H3N3-AA); 1397.52 (H4N3-AA); 1559.53 (H5N3-AA)
**Spectrum 16**	894.39 ++	894.34 ++	Hex3HexNAc5Fuc1-AA	**[M+H]+:** 365.90 (H1N1); 489.11 (N1F1-AA); 911.20 (H1N3-AA); 1057.27 (H1N3F1-AA); 1096.38 (H3N3); 1219.47 (H2N3F1-AA); 1381.60 (H3N3F1-AA); 1422.54 (H2N4F1-AA); 1584.59 (H3N4F1-AA)
**Spectrum 17**	902.38 ++	902.34 ++	Hex4HexNAc5-AA	**[M+H]+:** 365.92 (H1N1); 528.06 (H2N1); 731.15 (H2N2); 870.15 (H2N2-AA); 911.20 (H1N3-AA); 1073.34 (H2N3-AA); 1235.47 (H3N3-AA); 1438.58 (H3N4-AA); 1600.53 (H4N4-AA)
**Spectrum 18**	917.86 ++	917.84 ++	Hex4HexNAc3NeuNAc1Fuc1-AA	**[M+H]+:** 273.84^{2+} (N2-AA); 365.95 (H1N1); 489.07^{2+} (N1F1-AA); 528.12^{2+} (H2N1); 657.18 (H1N1SA1); 690.16^{2+} (H3N3F1-AA); 819.20 (H2N1SA1); 836.26 (H1N2F1-AA); 1016.35 (H2N2F1-AA); 1178.41 (H3N2F1-AA); 1381.64 (H4N2SA1); 1543.53 (H4N3F1-AA)
**Spectrum 19**	925.87 ++	925. 84 ++	Hex5HexNAc3NeuNAc1-AA	**[M+H]+:** 273.95^{2+} (N2-AA); 365.94 (H1N1); 528.09^{2+} (H2N1); 657.15 (H1N1SA1); 690.22 (H3N1); 819.15 (H2N1SA1); 870.27 (H3N1); 1032.34 (H3N2-AA); 1194.47 (H4N2-AA); 1397.43 (H4N3-AA); 1526.43 (H5N2SA1)
**Spectrum 20**	954.87 ++	954.86 ++	Hex5HexNAc4Fuc1-AA	**[M+H]+:** 365.97 (H1N1); 582.64^{2+} (H4N2); 1073.33 (H3N2); 1178.40 (H3N2F1-AA); 1402.45 (H3N4-AA); 1543.50 (H4N3F1-AA)
**Spectrum 21**	962.92 ++	962.86 ++	Hex6HexNAc4-AA	**[M+H]+:** 365.95 (H1N1); 528.10 (H2N1); 731.26 (H2N2); 852.26 (H4N1); 911.40 (H1N3-AA); 1217.38 (H3N3-AA); 1397.45 (H4N3-AA); 1559.53 (H5N3-AA); 1721.64 (H6N3-AA)

**Spectrum 22**	975.41 ++	975.37 ++	Hex4HexNAc5Fuc1-AA	**[M+H]+:** 365.96 (H1N1); 911.20 (H1N3-AA); 1057.32 (H1N3F1-AA); 1096.47 (H3N3); 1219.51 (H2N3F1-AA); 1381.60 (H3N3F1-AA); 1584.57 (H3N4F1-AA); 1746.66 (H4N4F1-AA)
**Spectrum 23**	983.42 ++	983.37 ++	Hex5HexNAc5-AA	**[M+H]+:** 365.91 (H1N1); 435.92^{2+} (H2N2-AA); 731.19 (H2N2); 911.24 (H1N3-AA); 1073.38 (H2N3-AA); 1235.45 (H3N3-AA); 1397.43 (H4N3-AA); 1461.45 (H4N4); 1600.58 (H4N4-AA)
**Spectrum 24**	998.88 ++	998.87 ++	Hex5HexNAc3NeuNAc1Fuc1-AA	**[M+H]+:** 365.96 (H1N1); 528.03 (H2N1); 657.15 (H1N1SA1); 852.16 (H4N1); 917.86^{2+} (H4N3SA1F1-AA); 1016.43 (H2N2F1-AA); 1178.44 (H3N2F1-AA); 1340.52 (H4N2F1-AA); 1526.44 (H3N3SA1-AA); 1705.59 (H5N3F1-AA)
**Spectrum 25**	1006.89 ++	1006.86 ++	Hex6HexNAc3NeuNAc1-AA	**[M+H]+:** 365.93 (H1N1); 528.03 (H2N1); 657.21 (H1N1SA1); 690.20 (H3N1); 763.60^{2+} (H3N3SA1-AA); 819.26 (H2N1SA1); 852.27 (H4N1); 1014.29 (H3N2-AA); 1032.31 (H5N1); 1194.43 (H4N2-AA); 1356.47 (H5N2-AA)
**Spectrum 26**	1019.42 ++	1019.38 ++	Hex4HexNAc4NeuNAc1Fuc1-AA	**[M+H]+:** 365.94 (H1N1); 657.18 (H1N1SA1); 782.24^{2+} (H4N4-AA); 1219.34 (H2N3F1-AA); 1381.49 (H3N3F1-AA); 1508.65 (H3N3SA1-AA)
**Spectrum 27**	1027.41 ++	1027.38 ++	Hex5HexNAc4NeuNAc1-AA	**[M+H]+ :** 365.95 (H1N1); 528.08 (H2N1); 657.17 (H1N1SA1); 893.24 (H1N3-AA); 1055.33 (H2N3-AA); 1235.46 (H3N3-AA); 1397.50 (H4N3-AA); 1526.52 (H5N2SA1); 1688.57 (H4N3SA1-AA)
**Spectrum 28**	1035.92 ++	1035.88 ++	Hex6HexNAc4Fuc1-AA	**[M+H]+:** 365.95 (H1N1); 528.08 (H2N1); 731.07 (H2N2); 911.20 (H1N3-AA); 1217.45 (H3N3-AA); 1381.48 (H3N3F1-AA); 1543.53 (H4N3F1-AA); 1705.64 (H5N3F1-AA); 1867.70 (H6N3F1-AA)
**Spectrum 29**	1056.42 ++	1056.40 ++	Hex5HexNAc5Fuc1-AA	**[M+H]+:** 365.94 (H1N1); 731.21 (H2N2); 893.25 (H1N3-AA); 1039.27 (H1N3F1-AA); 1258.40 (H2N3-AA); 1381.51 (H3N3F1-AA); 1584.57 (H3N4F1-AA); 1746.64 (H4N4F1-AA), 1908.69 (H5N4F1-AA)
**Spectrum 30**	1079.88 ++	1079.89 ++	Hex6HexNAc3NeuNAc1Fuc1-AA	**[M+H]+:** 365.98 (H1N1);528.15 (H2N1); 657.12 (H1N1SA1); 690.17 (H1N2-AA); 753.25^{2+} (H5N3F1-AA); 819.30 (H2N1SA1); 853.23^{2+} (H5N3F1-AA);1178.39 (H3N2F1-AA); 1340.48 (H4N2F1-AA); 1502.51 (H5N2F1-AA)
**Spectrum 31**	1100.42 ++	1100.41 ++	Hex5HexNAc4NeuNAc1Fuc1-AA	**[M+H]+:** 365.96 (H1N1); 528.12 (H2N1); 657.19 (H1N1SA1); 819.17 (H2N1SA1); 1016.36 (H2N1F1-AA); 1178.40 (H3N2F1-AA); 1381.51 (H3N3F1-AA); 1543.52 (H4N3F1-AA); 1729.65 (H5N3SA1); 1834.67 (H4N3SA1F1-AA)
**Spectrum 32**	1108.44 ++	1108.40 ++	Hex6HexNAc4NeuNAc1-AA	**[M+H]+:** 657.04 (H1N1SA1); 1397.50 (H4N3-AA); 1559.61 (H5N3-AA); 1721.67 (H6N3-AA); 1850.65 (H5N3SA1-AA)
**Spectrum 33**	1120.91 ++	1120.92 ++	Hex4HexNAc5NeuNAc1Fuc1-AA	**[M+H]+:** 365.96 (H1N1); 528.02 (H2N1); 657.18 (H1N1SA1); 911.40 (H1N3-AA); 1019.38 (H4N2); 1057.39 (H1N3F1-AA); 1219.51 (H2N3F1-AA); 1381.51 (H3N3F1-AA); 1584.56 (H3N4F1-AA); 2037.70 (H4N4SA1F1-AA)

**Spectrum 34**	1128.91 ++	1128.92 ++	Hex5HexNAc5NeuNAc1-AA	**[M+H]+:** 365.98 (H1N1); 528.14 (H2N1); 657.16 (H1N1SA1); 865.12^{2+} (H3N4SA1-AA); 911.18 (H1N3-AA); 966.712+ (H5N4SA1); 1073.39 (H4N2); 1235.46 (H3N3-AA); 1461.48 (H4N4); 1600.61 (H4N4-AA); 1688.61 (H4N3SA1-AA); 1891.60 (H4N4SA1-AA)
**Spectrum 35**	1172.93 ++	1172.92 ++	Hex5HexNAc4NeuNAc2-AA	**[M+H]+:** 365.96 (H1N1); 528.15 (H2N1); 657.18 (H1N1SA1); 819.13 (H2N1SA1); 870.27 (H2N2-AA); 1032.33 (H3N2-AA); 1346.39 (H3N2SA1); 1549.52 (H4N3SA1); 1688.57 (H3N3SA1-AA); 2002.49 (H5N3SA2)
**Spectrum 36**	1181.45 ++	1181.43 ++	Hex6HexNAc4NeuNAc1Fuc1-AA	**[M+H]+:** 365.89 (H1N1); 657.15 (H1N1SA1); 819.14 (H2N1SA1); 893.20 (H1N3-AA); 1165.04 (H2N3
**Spectrum 37**	1201.95 ++	1201.96 ++	Hex5HexNAc5NeuNAc1Fuc1-AA	**[M+H]+:** 365.94 (H1N1); 657.15 ((H1N1SA1); 893.23 (H3N2); 911.38 (H1N3-AA); 1219.40 (H2N3F1); 1381.49 H3N3F1-AA); 2037.74 (H4N4SA1F1-AA); 2199.70 (H5N4SA1F1-AA)
**Spectrum 38**	1245.97 ++	1245.95 ++	Hex5HexNAc4NeuNAc2Fuc1-AA	**[M+H]+:** 356.93 (H1N1); 528.08 (H2N1); 657.17 (H1N1SA1); 870.26 (H2N2-AA); 1055.35 (H4N2); 1178.41 (H3N2F1-AA); 1346.47 (H3N2SA1); 1543.52 (H4N3F1-AA); 1672.60 (H3N3SA1F1-AA); 1834.66 (H4N3SA1F1-AA)
**Spectrum 39**	1274.46 ++	1274.46 ++	Hex5HexNAc5NeuNAc2-AA	**[M+H]+**: 366.04 (H1N1); 657.09 (H1N1SA1); 875.25 (H1N3-AA); 981.71 (H3N1SA1); 1096.46 (H3N3); 1379.70 (H4N3-AA); 1752.52 (H4N4SA1); 1891.52 (H4N4SA1-AA)
**Spectrum 40**	898.67 +++	898.66 +++	Hex5HexNAc5NeuNAc2Fuc1-AA	**[M+H]+:** 273.872+ (H2N1); 365.95 (H1N1); 454.12 (H1SA1); 657.23 (H1N1SA1); 911.25 (H1N3-AA); 1039.62 (H1N3F1-AA); 1219.47 (H2N3F1-AA); 1381.43 (H3N3F1-AA); 2037.74 (H4N4SA1F1-AA)
**Spectrum 41**	923.39 +++	923.34 +++	Hex6HexNAc6NeuNAc1Fuc1-AA	**[M+H]+:** 273.90^{2+} (H2N1); 365.94 (H1N1); 489.05 (N1F1-AA); 657.21 (H1N1SA1); 911.00 (H1N3-AA); 1022.16 (H2N2SA1); 1056.45^{2+} (H5N5F1-AA); 1381.40 (H3N3F1-AA); 1746.69 (H4N4F1-AA); 2111.68 (H5N5F1-AA)
**Spectrum 42**	952.67 +++	952.68 +++	Hex6HexNAc5NeuNAc2Fuc1-AA	**[M+H]+:** 273.88 (H2N1); 365.95 (H1N1); 657.18 (H1N1SA1); 1184.32 (H3N2SA1); 1381.49 (H3N3F1-AA); 1543.55 (H4N3F1-AA); 1834.67 (H4N3SA1F1-AA)
**Spectrum 43**	977.37 +++	977. 36 +++	Hex7HexNAc6NeuNAc1Fuc1-AA	**[M+H]+:** 366.01 (H1N1); 508.44^{2+} (H2N2F1-AA); 528.09 (H2N1); 657.26 (H1N1SA1); 846.24^{2+} (H4N4F1-AA); 1124.31 (H3N2F1-AA); 1381.56 (H3N3F1-AA); 1387.44 (H3N3SA1); 1623.85 (H5N4); 1908.60 (H5N4F1-AA); 2199.69 (H5N4SA1F1-AA)
**Spectrum 44**	1020.41 +++	1020.37 +++	Hex6HexNAc6NeuNAc2Fuc1-AA	**[M+H]+:** 365.93 (H1N1); 657.17 (H1N1SA1); 816.11 (H2N2); 911.40 (H1N3-AA); 1003.45 (H4N6); 1057.21 (H1N3F1-AA); 1219.41 (H2N3F1-AA); 1381.48 (H3N3F1-AA); 1746.65 (H4N4F1-AA); 2037.72 (H4N4SA1F1-AA)
**Spectrum 45**	1045.07 +++	1045.05 +++	Hex7HexNAc7NeuNAc1Fuc1-AA	**[M+H]+:** 365.95 (H1N1); 657.26 (H1N1SA1); 911.40 (H1N3-AA); 1039.60 (H1N3F1-AA); 1384.50 (H5N3); 1607.46 (H2N5F1-AA); 1746.51 (H4N4F1-AA); 2111.74 (H5N5F1-AA)

**Spectrum 46**	1049.70 +++	1049.71 +++	Hex6HexNAc5NeuNAc3Fuc1-AA	**[M+H]+:** 365.98 (H1N1); 528.07 (H2N1); 657.15 (H1N1SA1); 937.67^{2+} (H4N4SA1-AA); 1245.97^{2+} (H5N4SA2F1-AA); 1543.50 (H4N3F1-AA); 1672.34 (H3N3SA1F1-AA); 1834.66 (H4N3SA1F1-AA); 2199.73 (H5N4SA1F1-AA)
**Spectrum 47**	1074.41 +++	1074.39 +++	Hex7HexNAc6NeuNAc2Fuc1-AA	**[M+H]+:** 365.95 (H1N1); 657.17 (H1N1SA1); 819.20 (H2N1SA1); 835.72 (H1N2F1-AA); 939.91^{2+} (H5N4SA1); 1181.47 (H3N3-AA); 1282.98^{2+} (H6N5SA1F1-AA); 1381.51 (H3N3F1-AA);1543.63 (H4N3F1-AA); 1711.65 (H5N3SA1); 1908.68 (H5N4F1-AA)
**Spectrum 48**	1171.41 +++	1171.42 +++	Hex7HexNAc6NeuNAc3Fuc1-AA	**[M+H]+:** 365.94 (H1N1); 465.04^{2+} (H3N2); 657.17 (H1N1SA1); 1156.34 (H6N6); 1178.20 (H3N2F1-AA); 1364.64 (H4N2SA1); 1428.44^{2+} (H6N5SA2F1-AA); 1834.61 (H4N3SA1F1-AA); 2199.71 (H5N4SA1F1-AA)
