# Supplementary material for: The glycomic effect of N-acetylglucosaminyltransferase III overexpression in metastatic melanoma cells. GnT-III modifies highly branched N-glycans
Source: Glycoconj J. 2018 Mar 3;35(2):217–31. doi: 10.1007/s10719-018-9814-y (PMC5916991; doi:10.1007/s10719-018-9814-y)

**Supplementary Figure 1. MS/MS spectra of N-glycan structures detected in WM266-4-pIRESneo and WM266-4-GnT-III cells.** The 2-AA-labeled glycans released from the membrane and secreted proteins were analyzed by ESI-iontrap-MS/MS (positive-ion mode). The obtained fragmentation spectra, supported by general knowledge of human glycobiology, were used for structural assignments of glycans. In some cases, the presence of structural and linkage isomers cannot be excluded. The MS/MS fragmentation spectra were interpreted manually basing on the fragmentation data from GlycoWorkbench software. Glycan compositions as well as proposed schematic structures are given where possible. The spectrum numbers refer to Supplementary Table1. Glycan schemes were prepared using GlycoWorkbench (*Red triangle*, dHex, fucose, F; *yellow circle*, Hex, galactose, H; *green circle,* Hex, mannose, H; *blue square*, HexNAc, *N*-acetylglucosamine, N; *Purple diamond*, NeuNAc, sialic acid, SA).

**Spectrum 1** 1032.34 (H3N2-AA+)

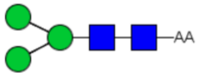


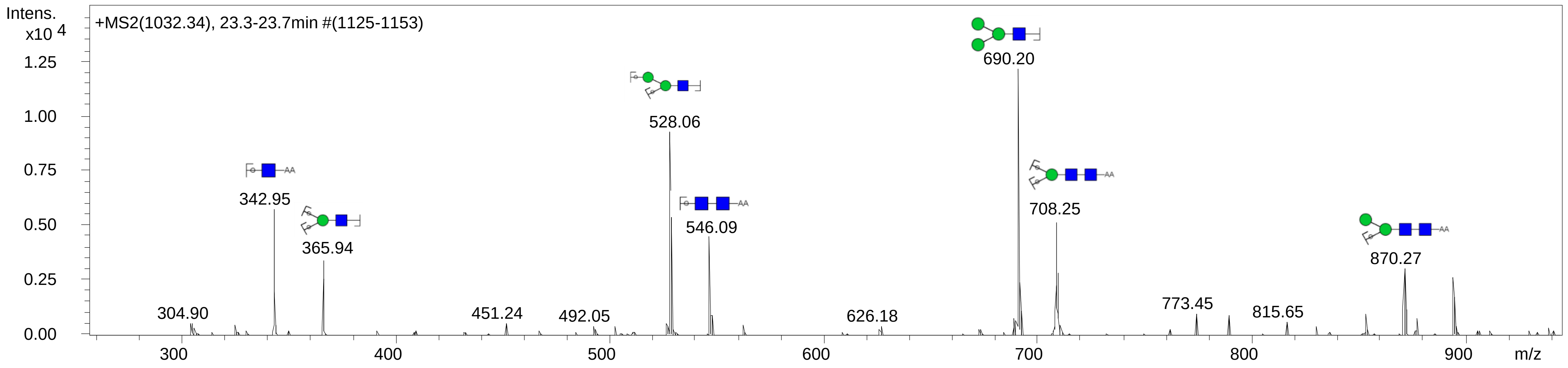


**Spectrum 2** 589.90 (H3N2F1-AA++)

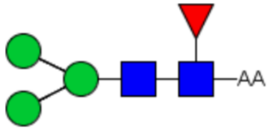


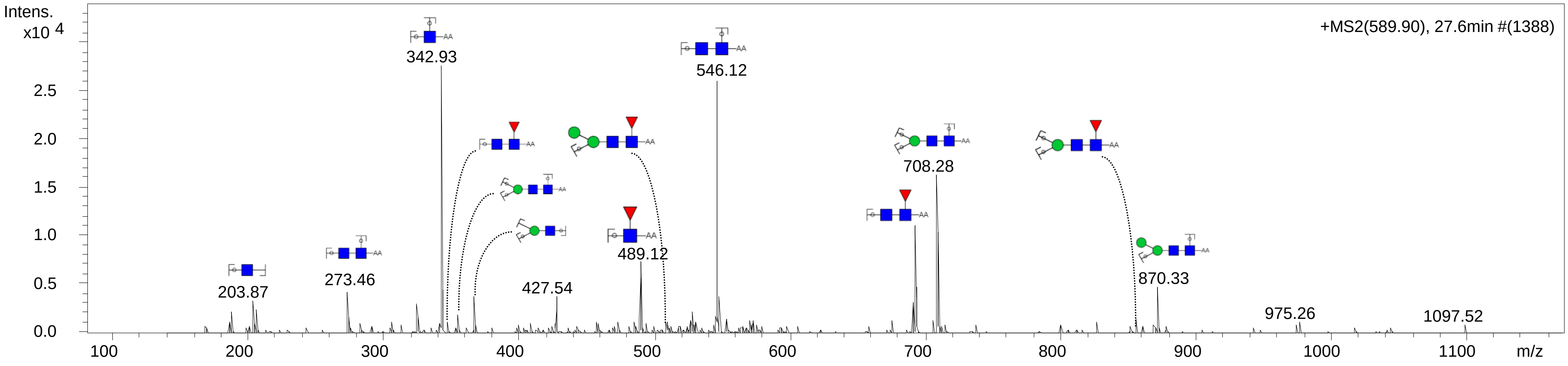

**Spectrum 3** 678.35 H5N2-AA++

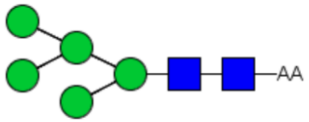


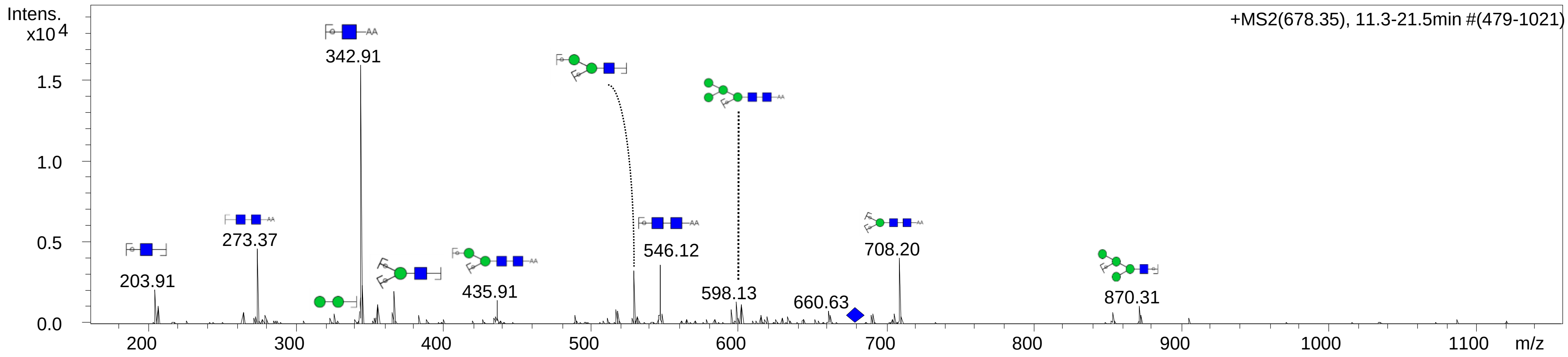

**Spectrum 4** 691.40 (H3N3F1-AA++)

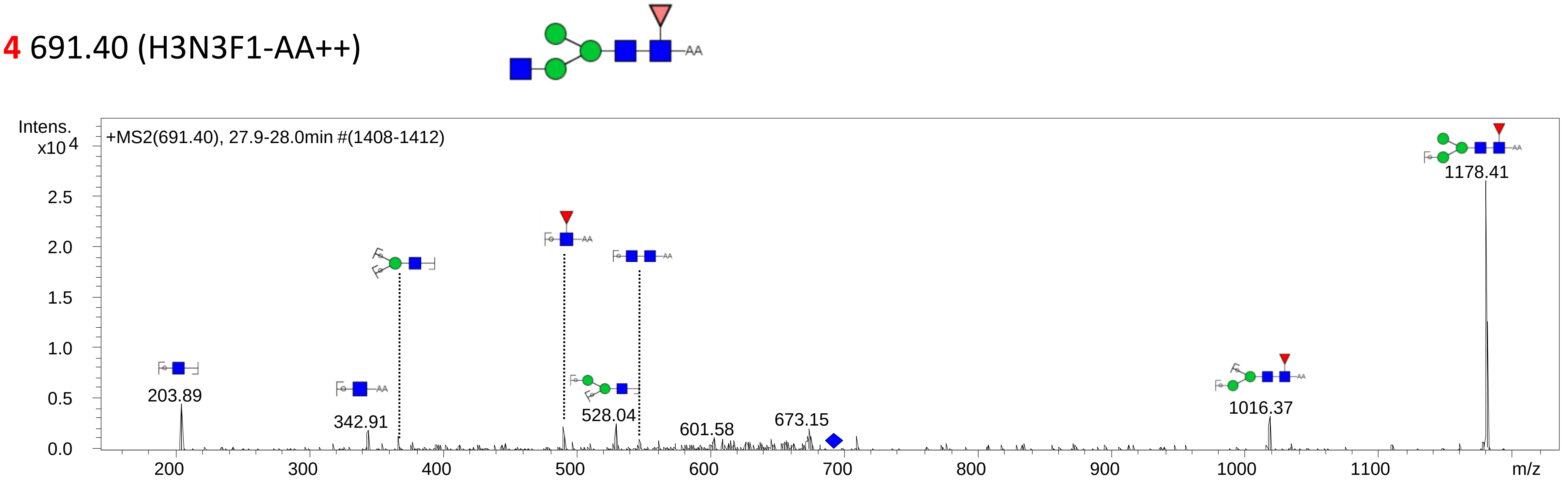


**Spectrum 5** 699.49 (H4N3-AA++)

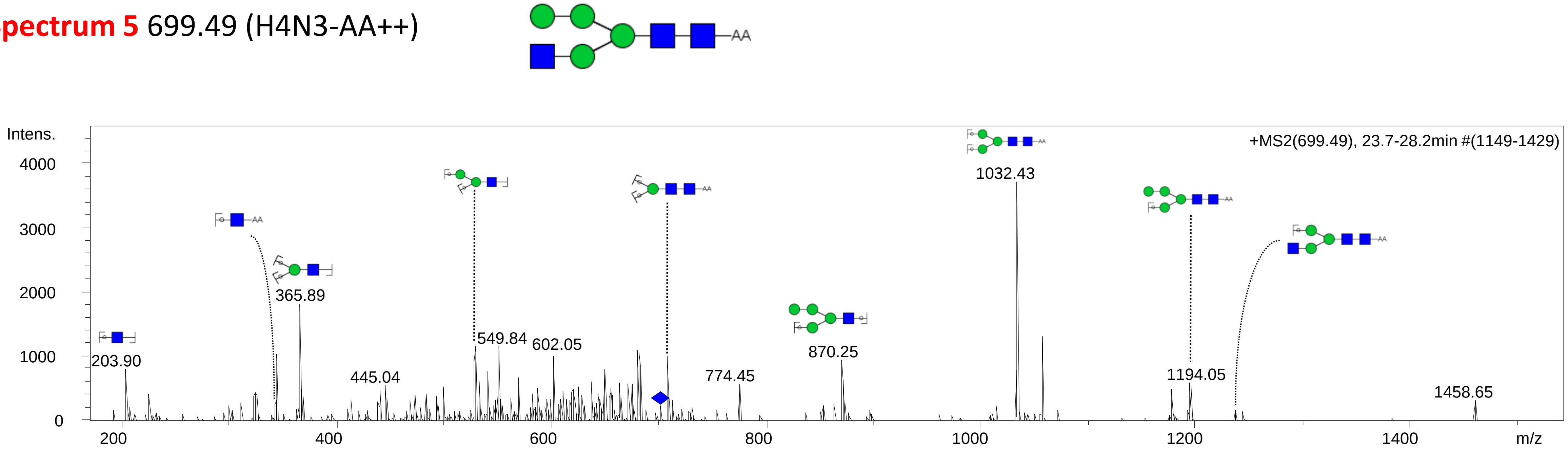

**Spectrum 6** 752.12 (H5N2F1-AA++)

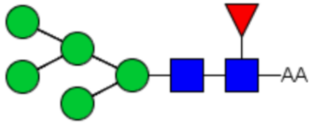


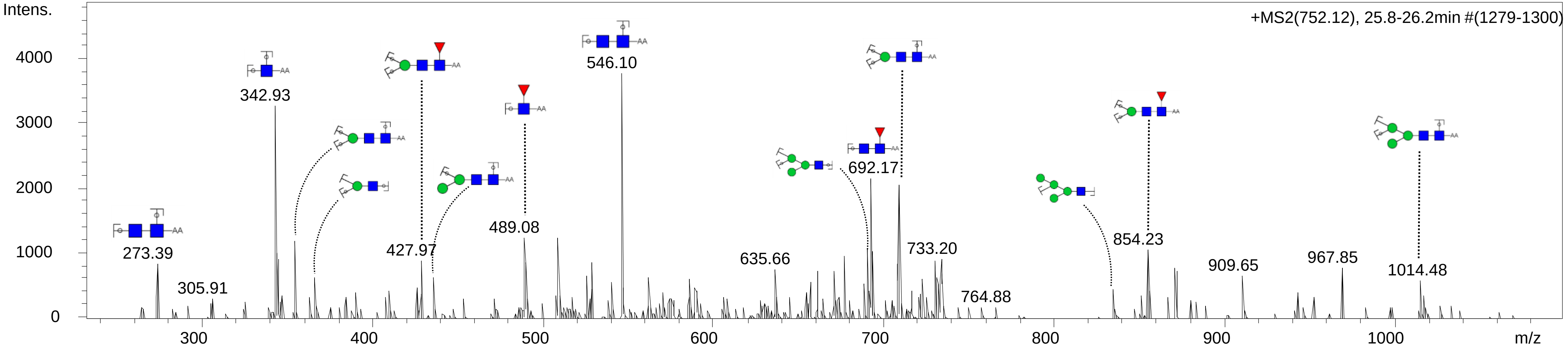


**Spectrum 7** 759.58 (H6N2-AA++)

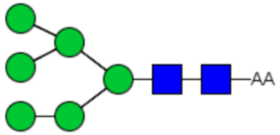


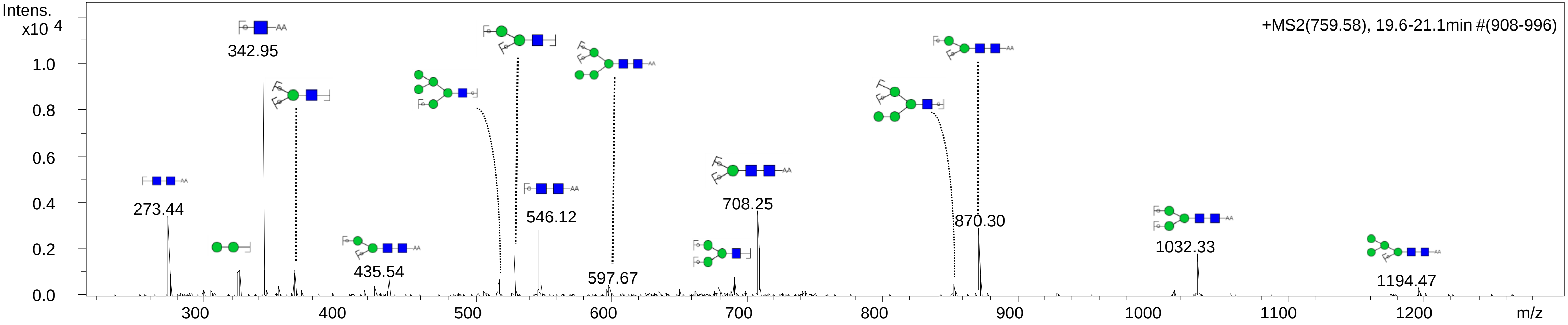

**Spectrum 8** 772.67 (H4N3F1-AA++)

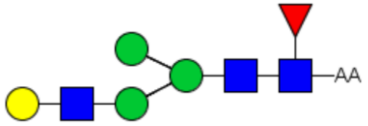


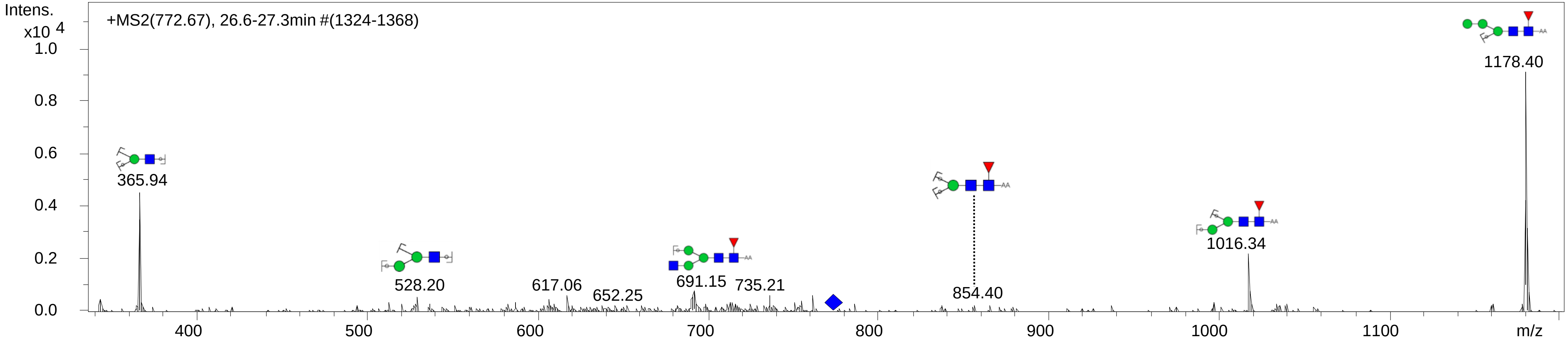


**Spectrum 9** 792.88 (H3N4F1-AA++)

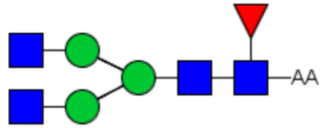


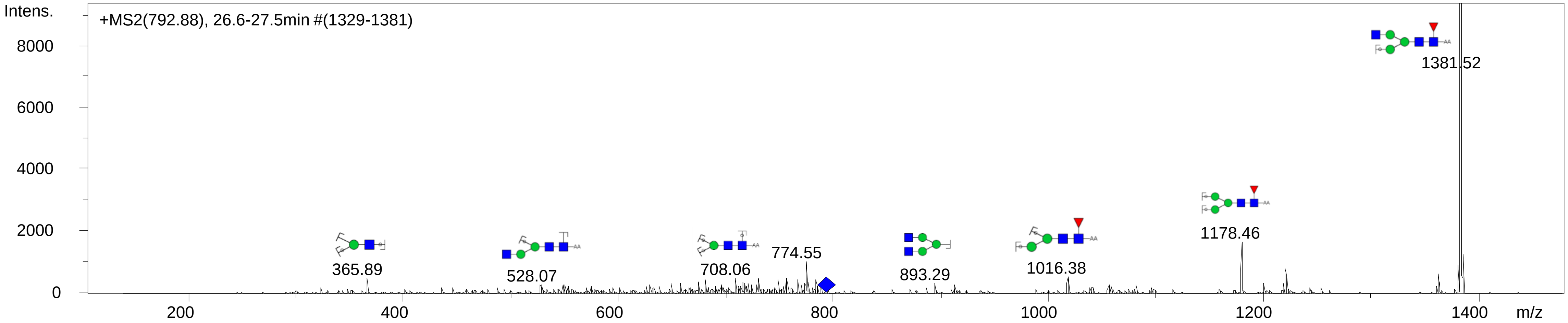

**Spectrum 10** 801.09 (H4N4-AA++)

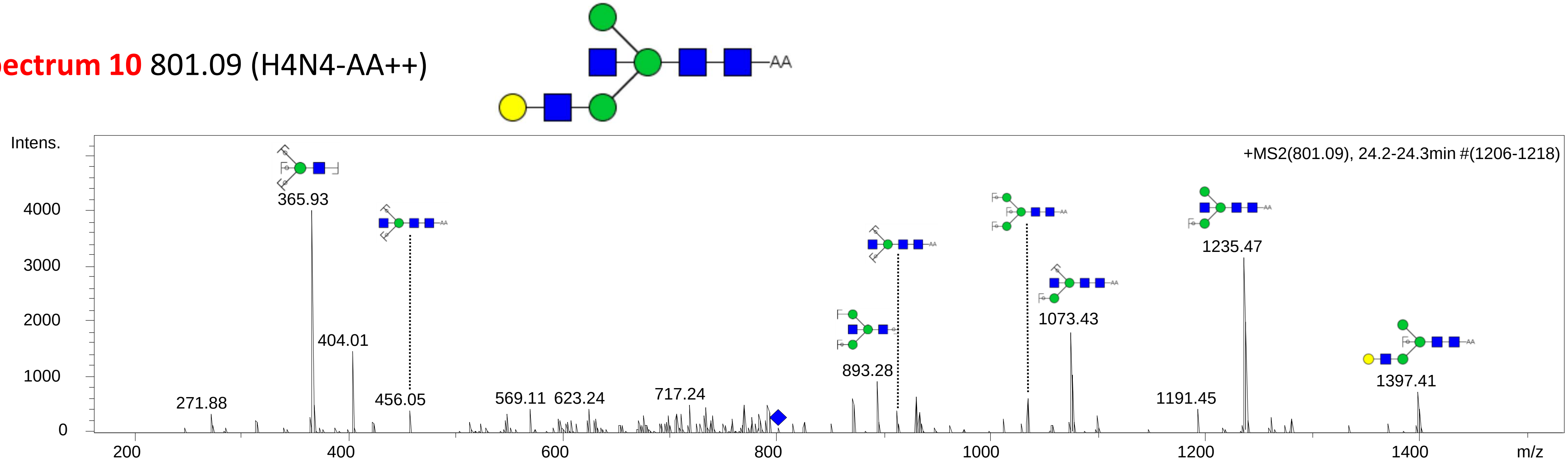


**Spectrum 11** 821.67 (H3N5-AA++)

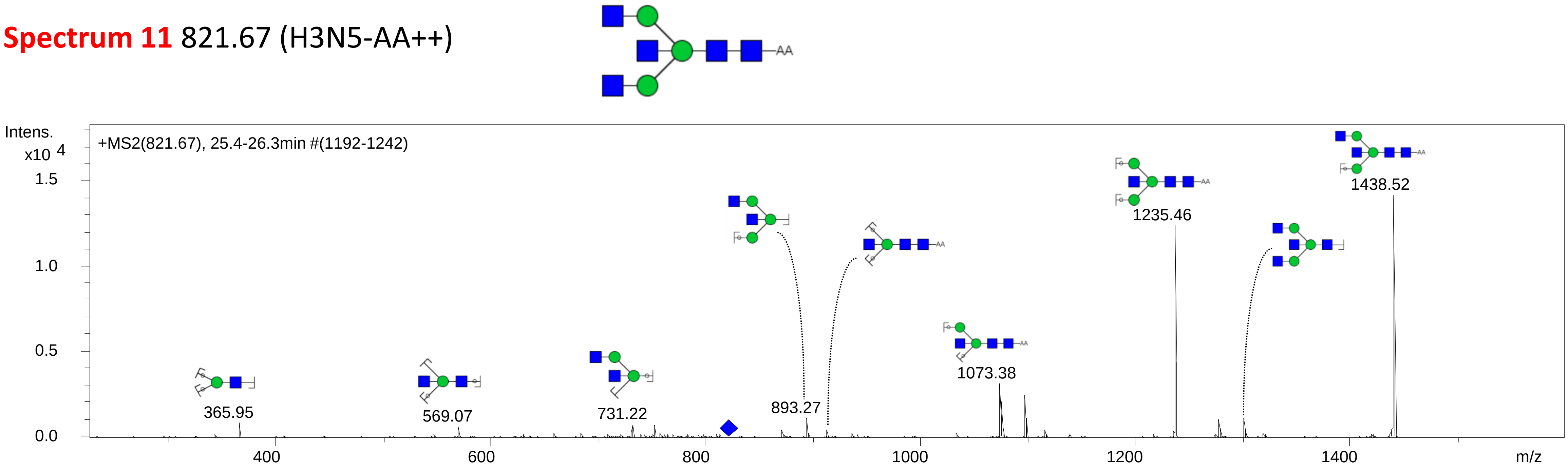

**Spectrum 12** 841.29 (H7N2-AA++)

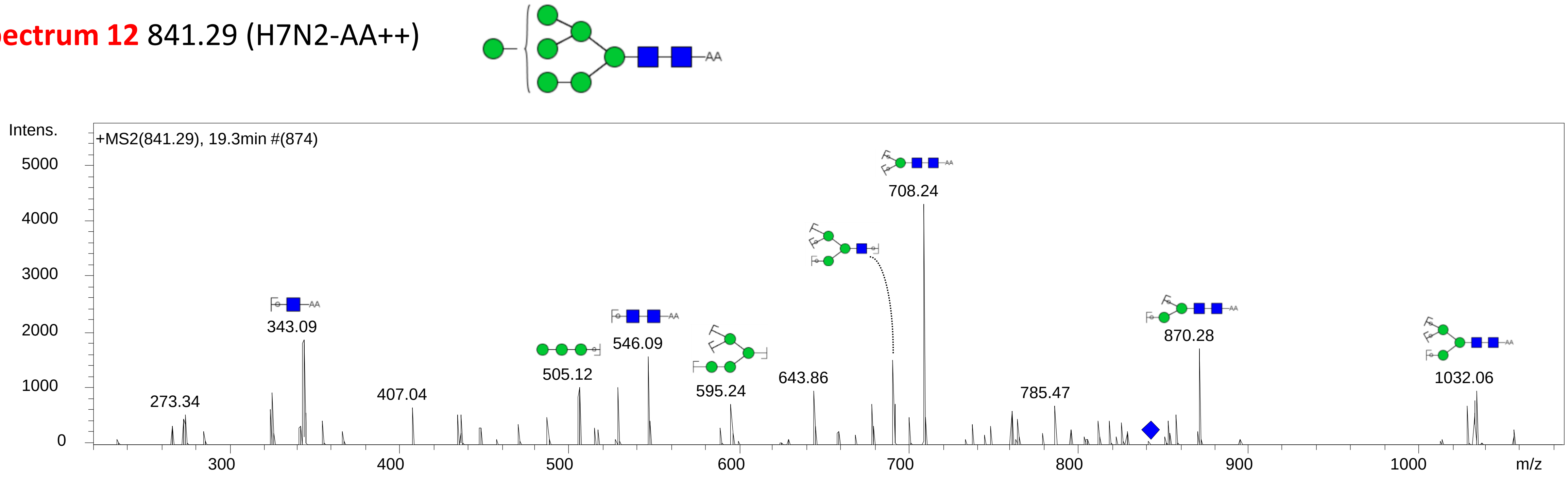


**Spectrum 13** 845.16 (H4N3SA1-AA++)

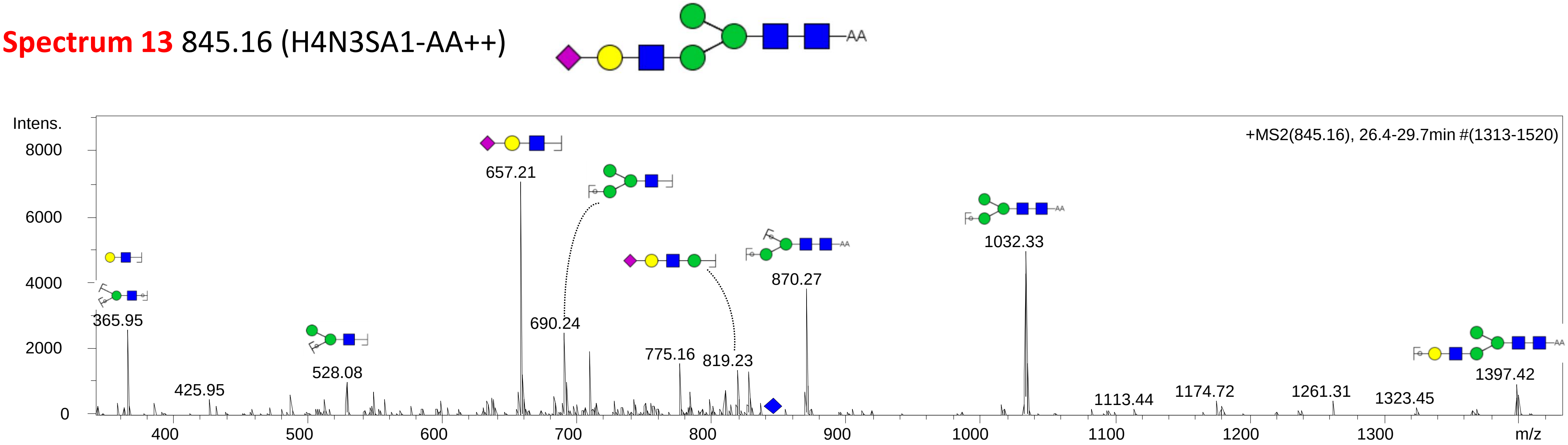

**Spectrum 14** 874.58 (H4N4F1-AA++) 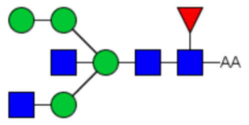 and 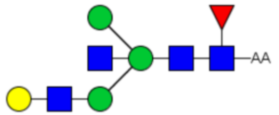

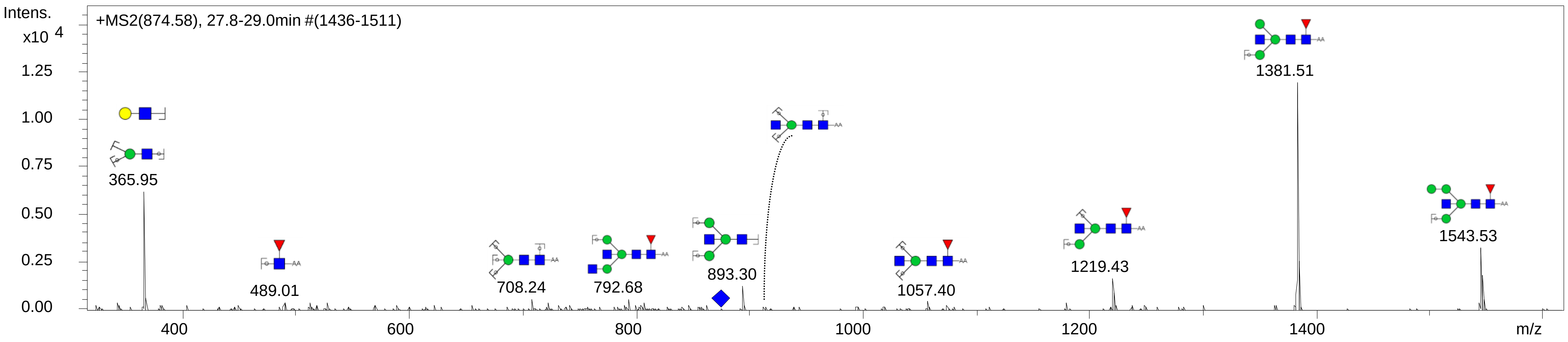


**Spectrum 15** 882.47 (H5N4-AA++) 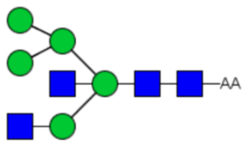

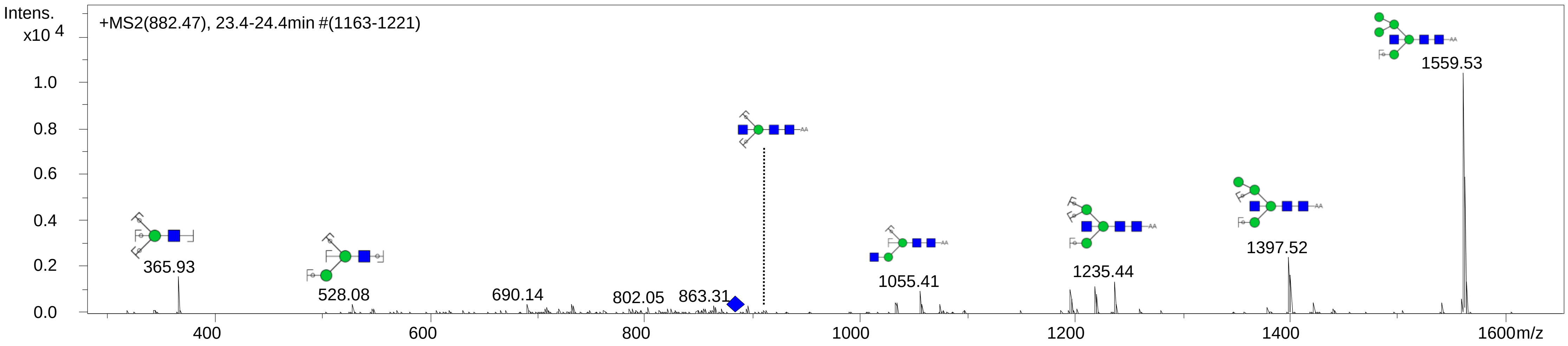

**Spectrum 16** 894.84 (H3N5F1-AA++)

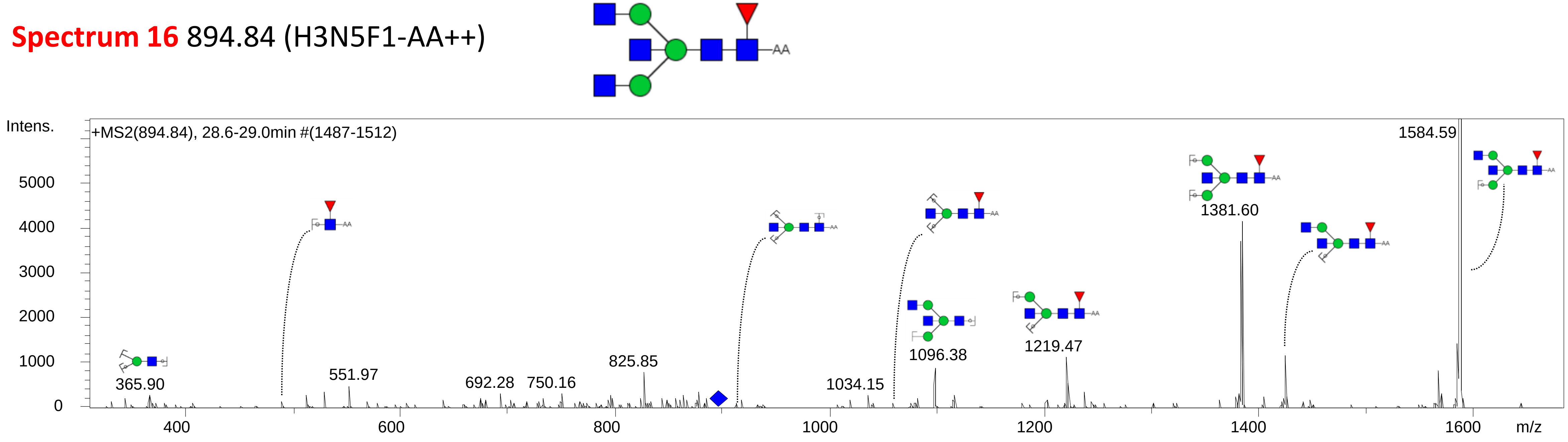


**Spectrum 17** 902.54 (H4N5-AA++)

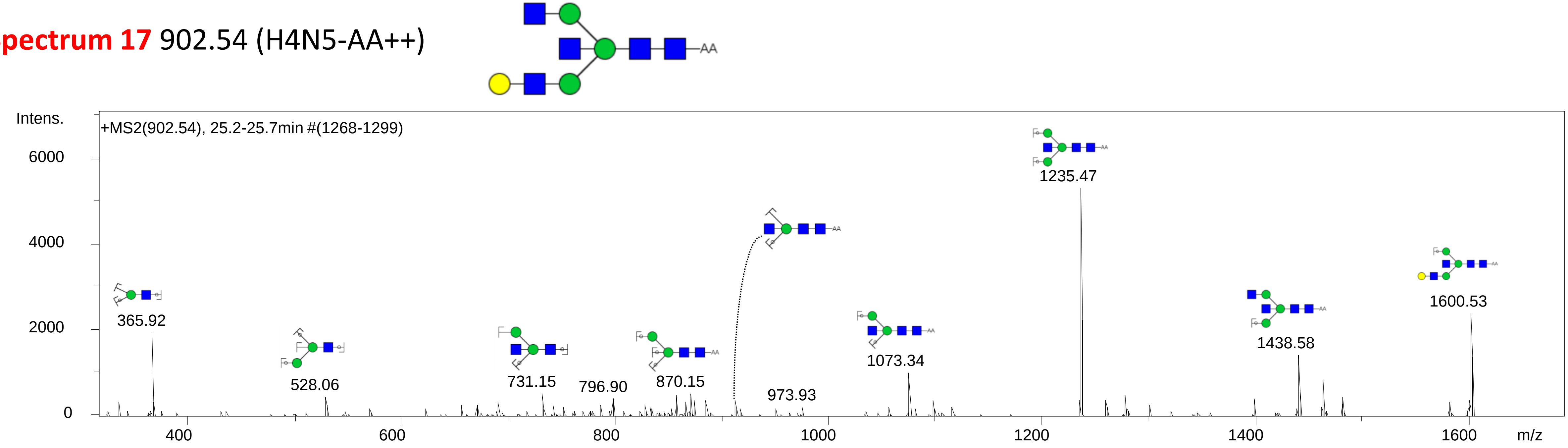

**Spectrum 18** 917.88 (H4N3SA1F1-AA++)

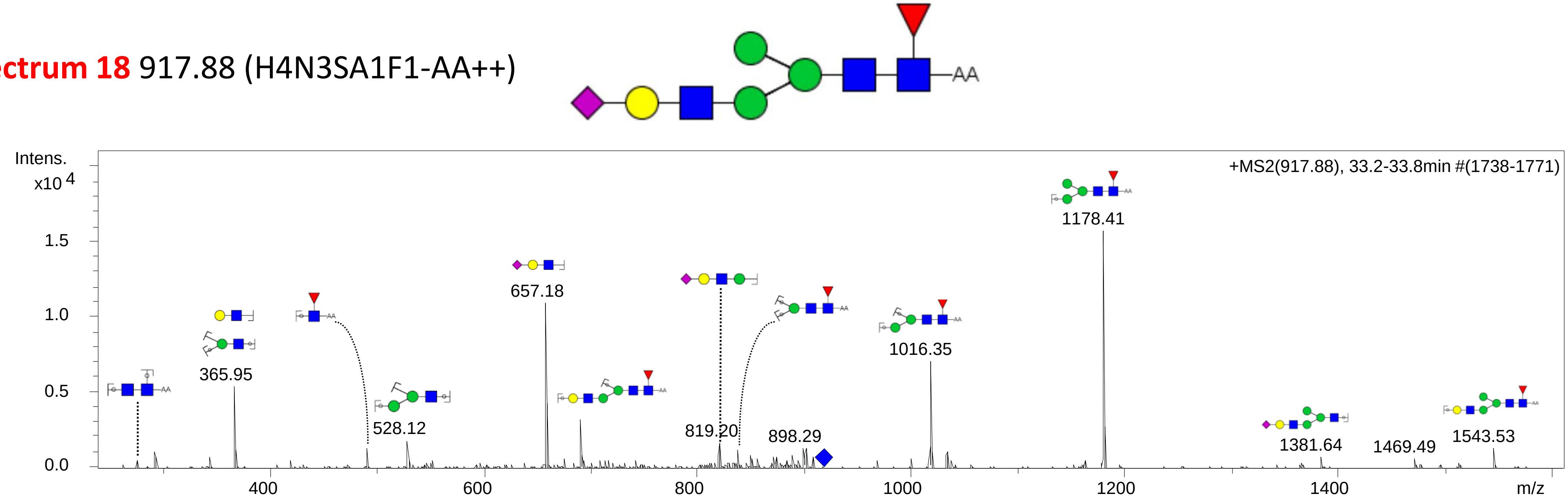

**Spectrum 19** 926.28 (H5N3SA1-AA++)

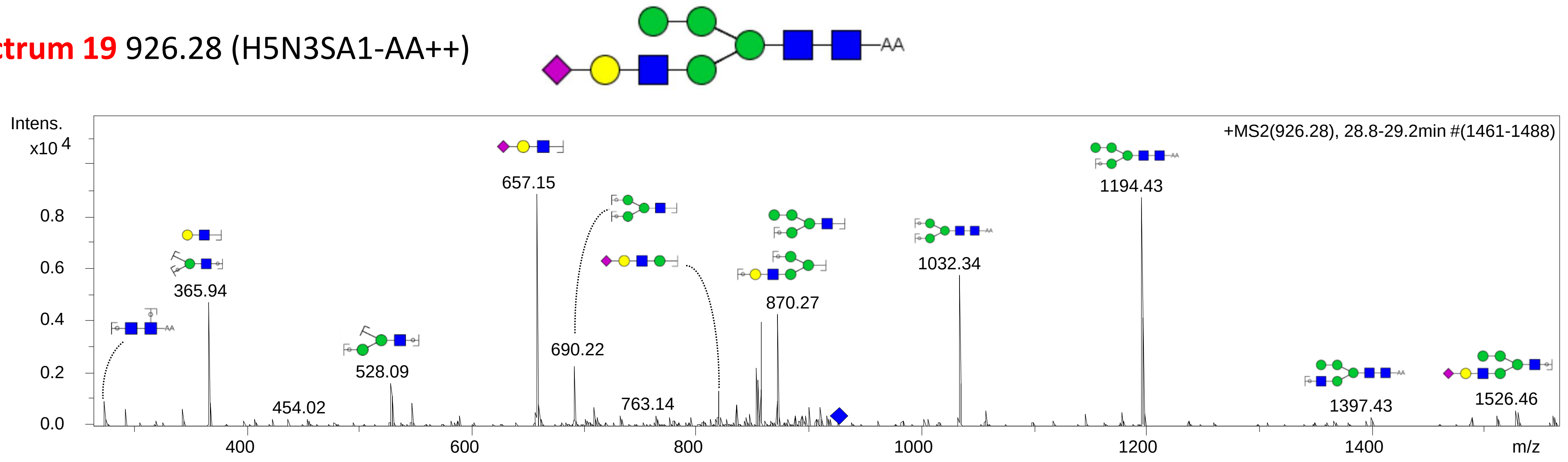


**Spectrum 20** 954.88 (H5N4F1-AA++)

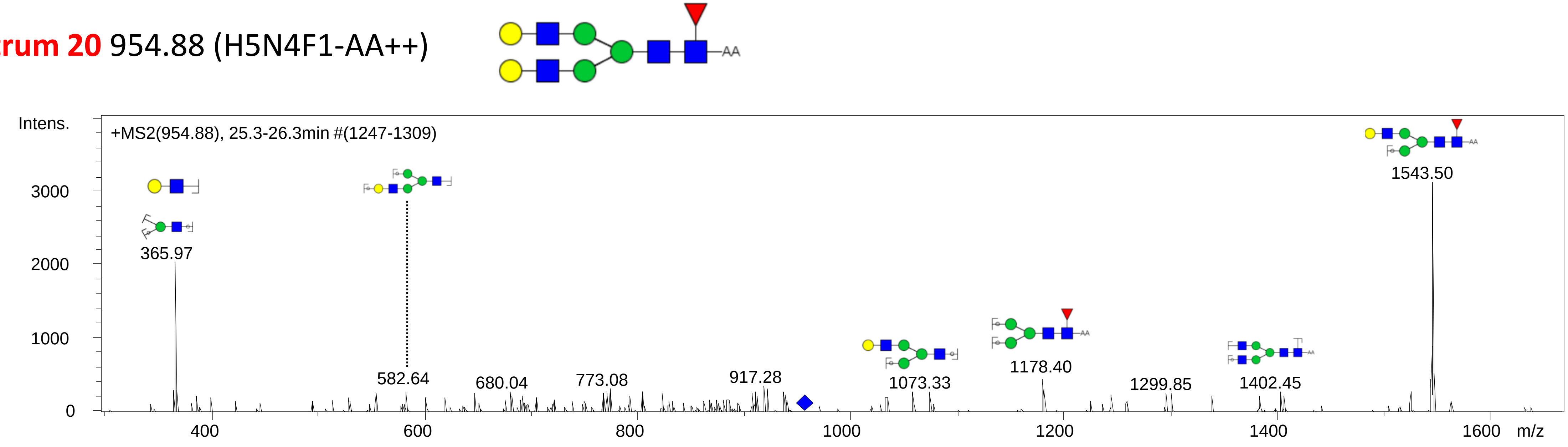

**Spectrum 21** 963.32 (H6N4-AA++)

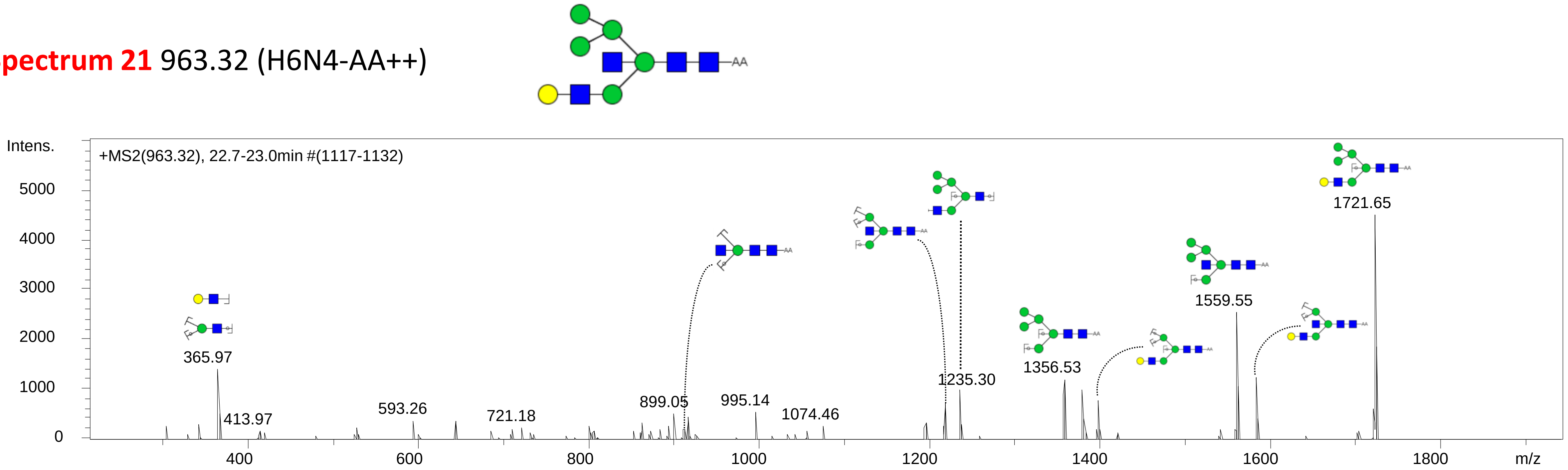


**Spectrum 22** 975.63 (H4N5F1-AA++)

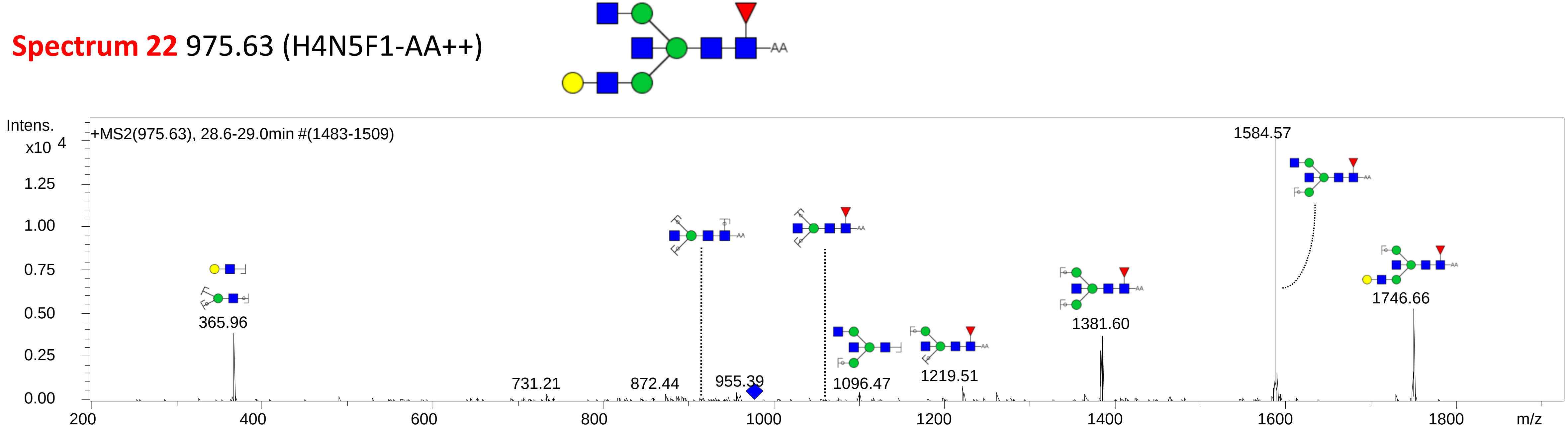

**Spectrum 23** 983.42 (H5N5-AA++)

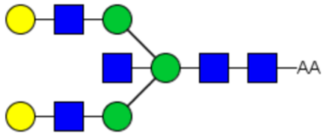


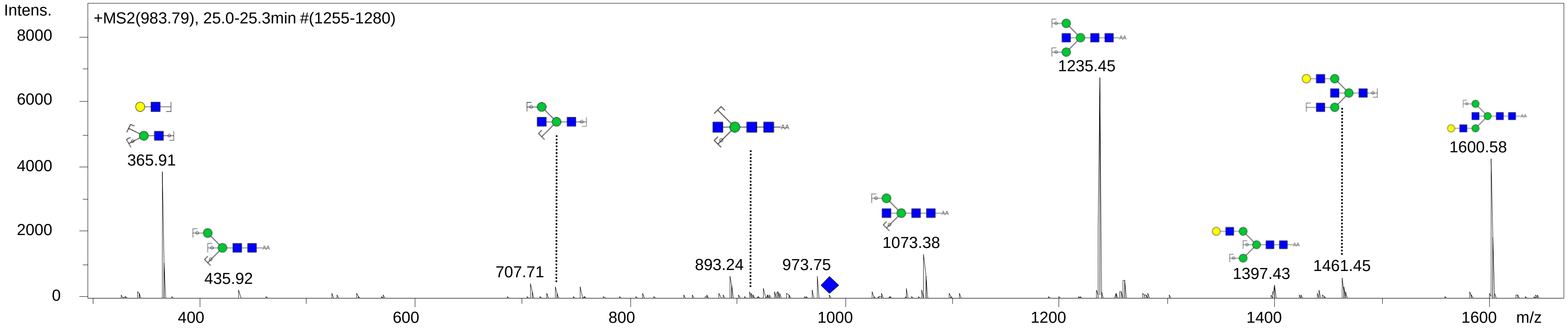


**Spectrum 24** 999.20 (H5N3SA1F1-AA++)

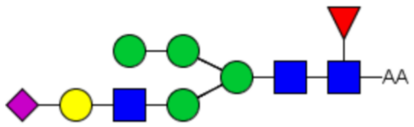


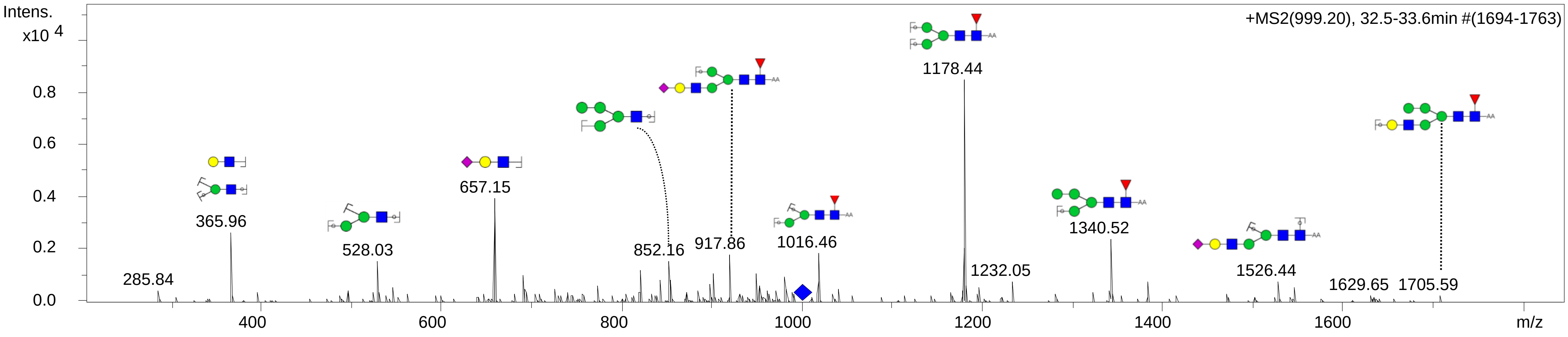

**Spectrum 25** 1007.52 (H6N3SA1-AA++)

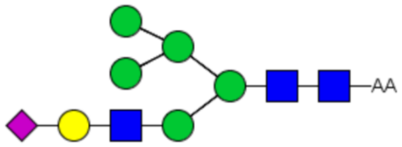


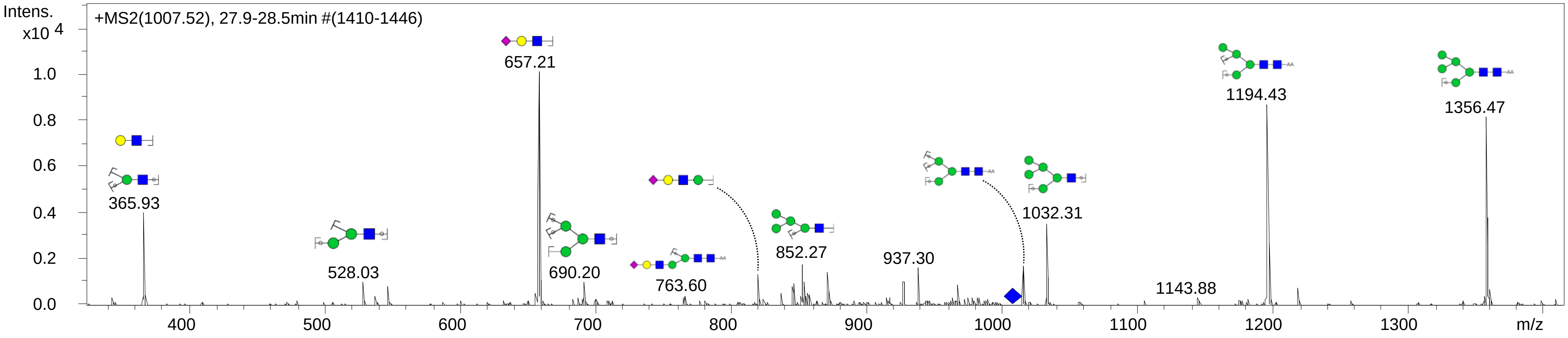

**Spectrum 26** 1019.60 (H4N4SA1F1-AA++)

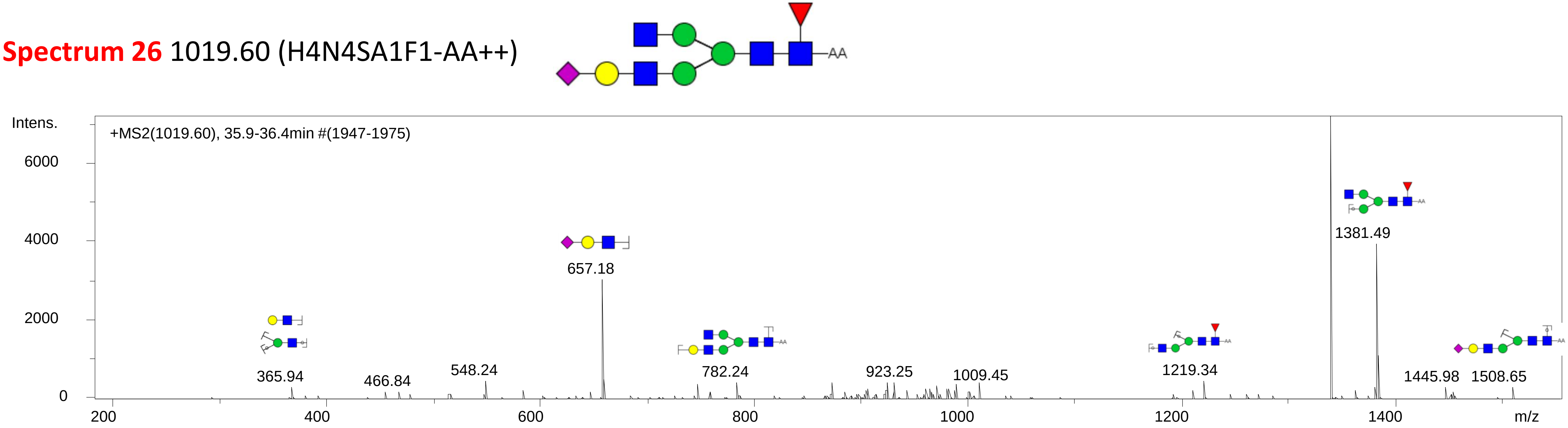


**Spectrum 27** 1027.65 (H5N4SA1-AA++)

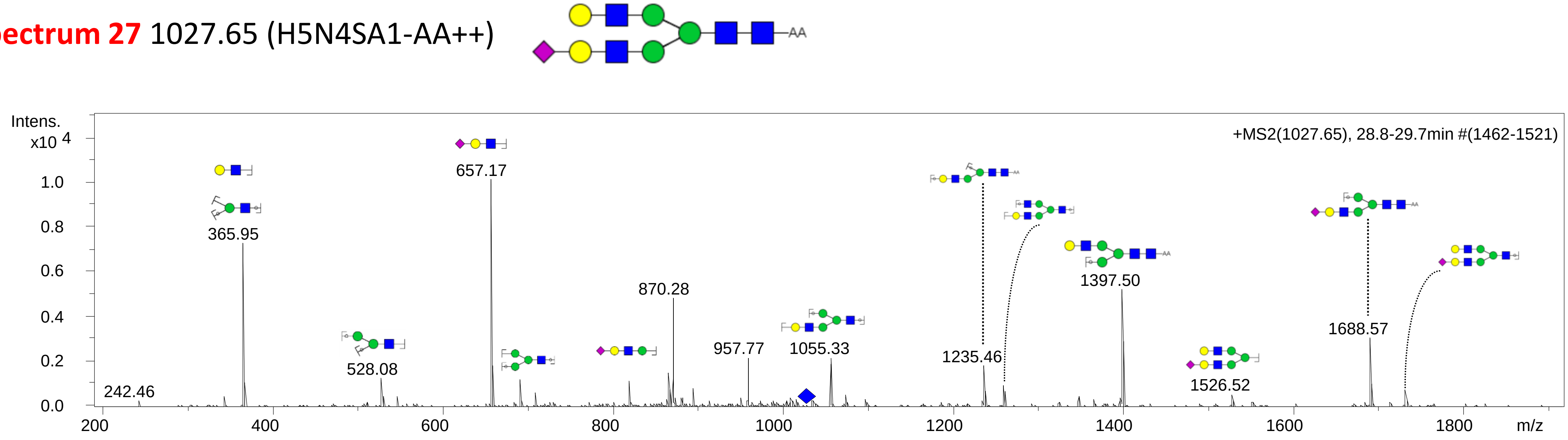

**Spectrum 28** 1036.21 (H6N4F1-AA++)

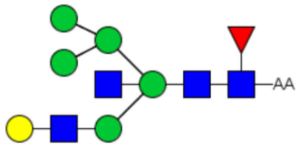


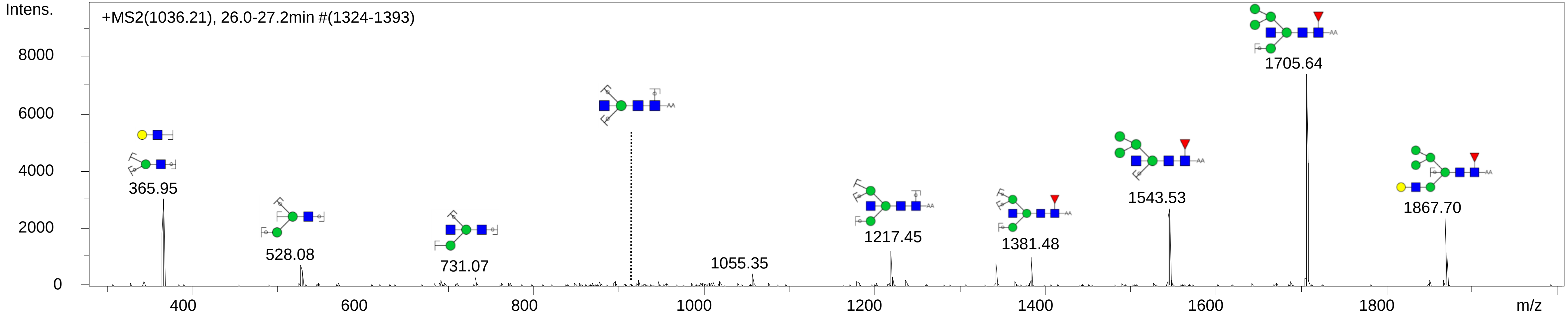

**Spectrum 29** 1056.92 (H5N5F1-AA++)

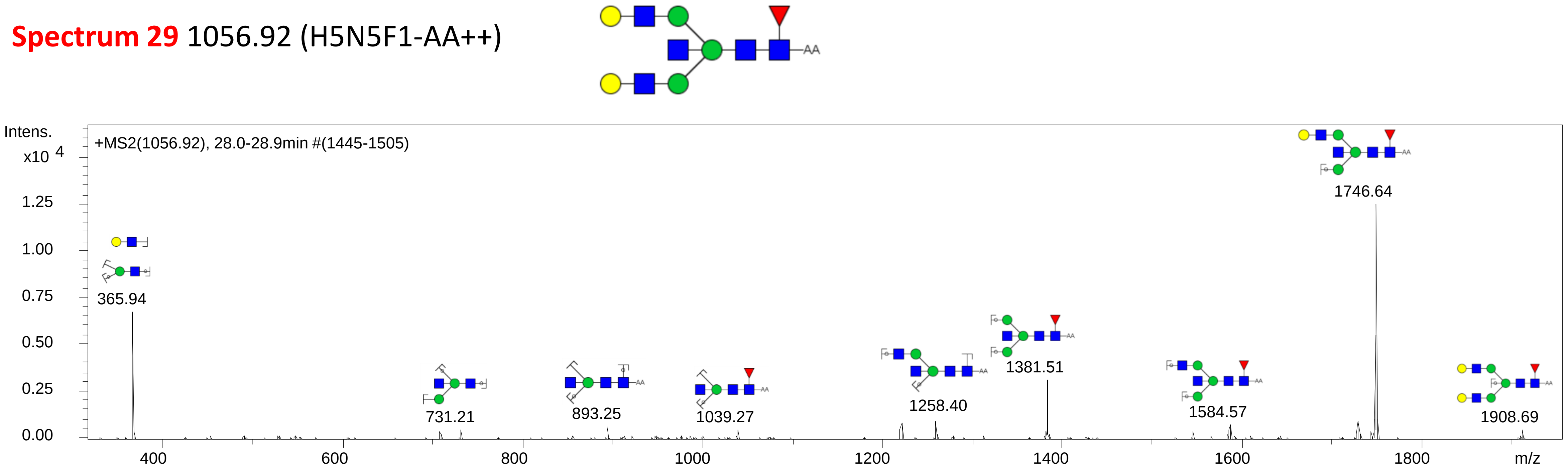


**Spectrum 30** 1080.26 (H6N3SA1F1-AA++)

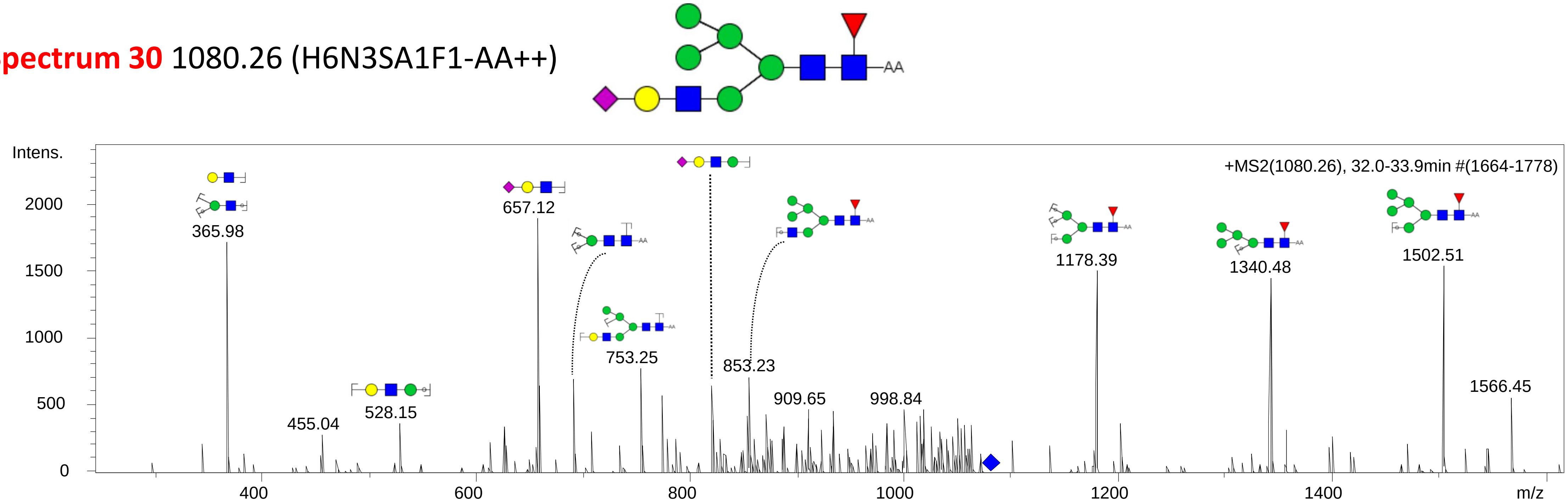

**Spectrum 31** 1101.00 (H5N4SA1F1-AA++)

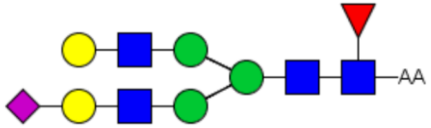


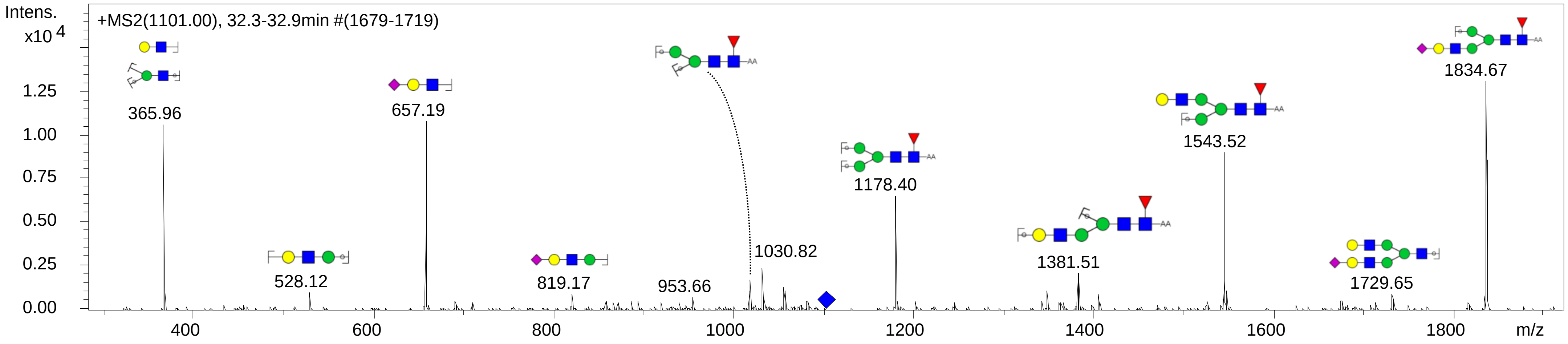


**Spectrum 32** 1108.96 (H6N4SA1-AA++)

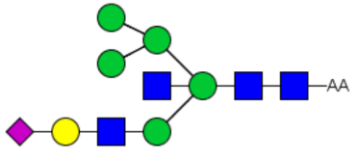


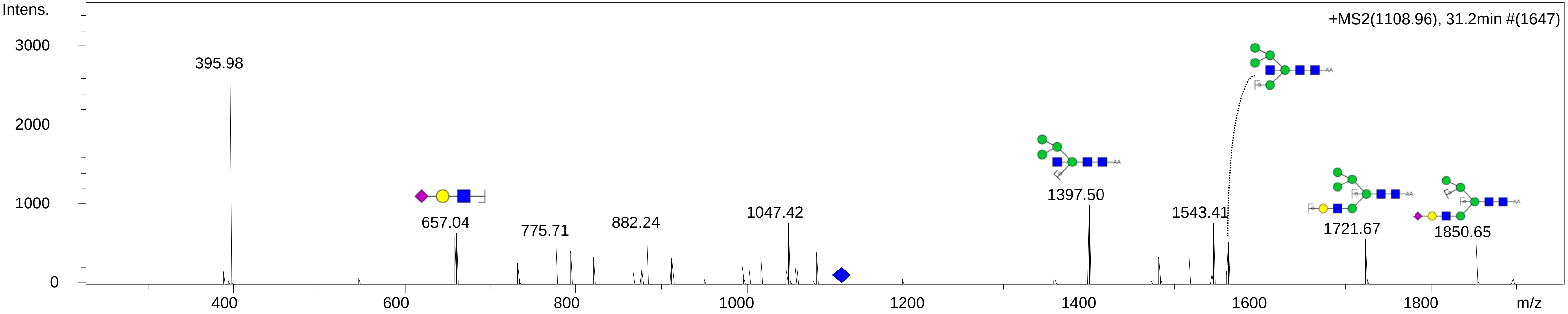

**Spectrum 33** 1121.33 (H4N5SA1F1-AA++)

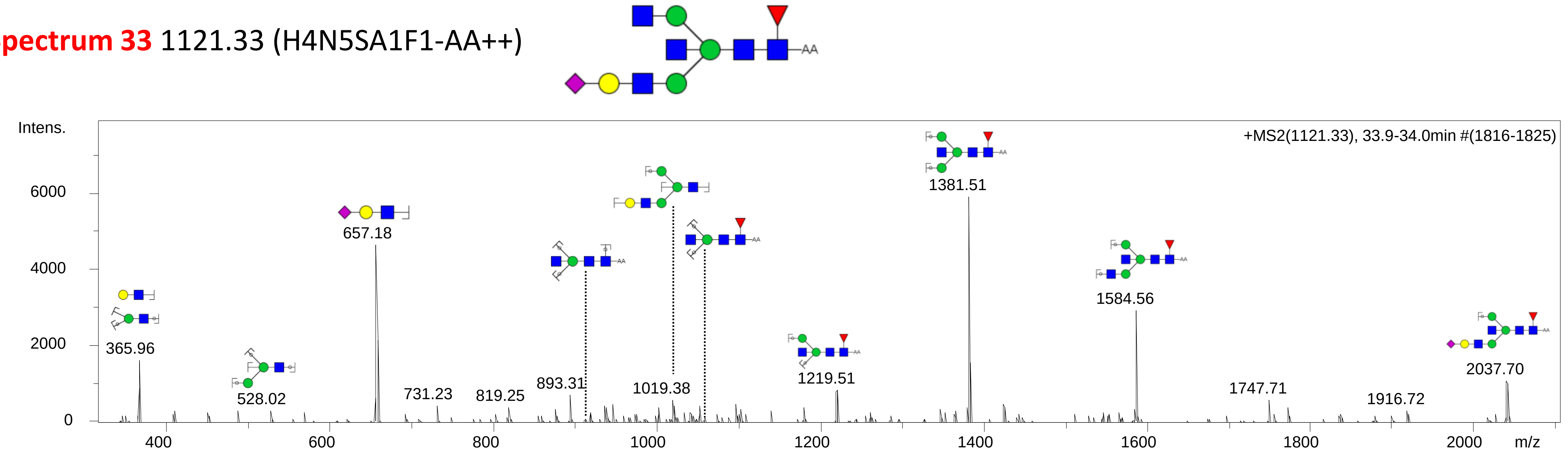


**Spectrum 34** 1129.37 (H5N5SA1-AA++)

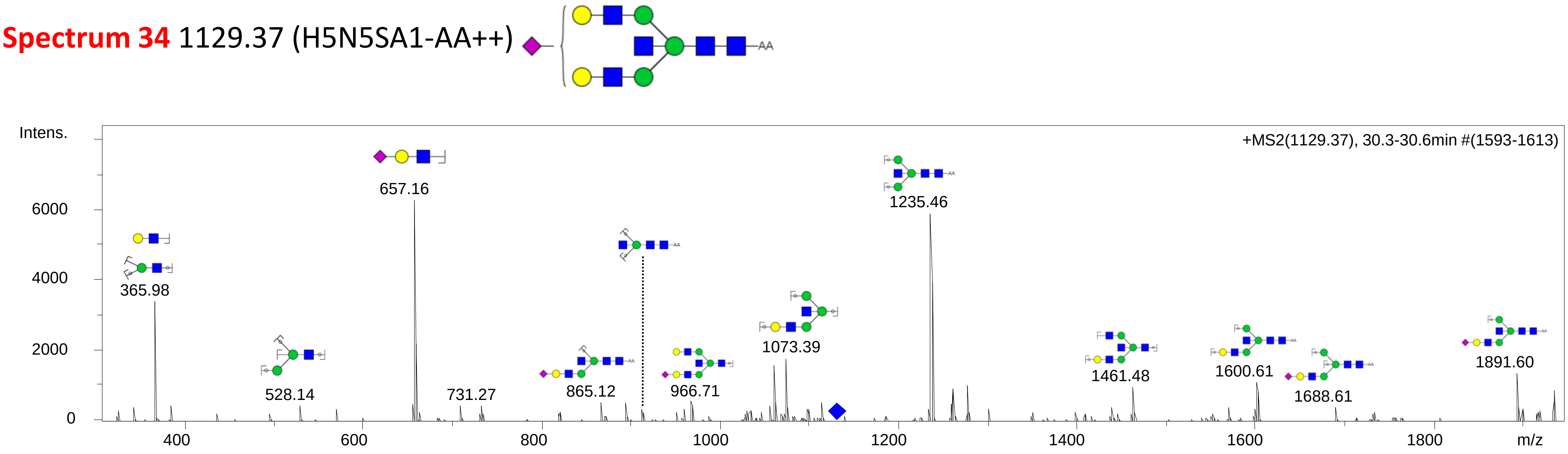

**Spectrum 35** 1172.16 (H5N4SA2-AA++)

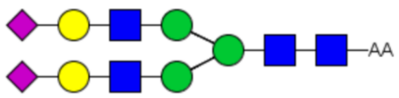


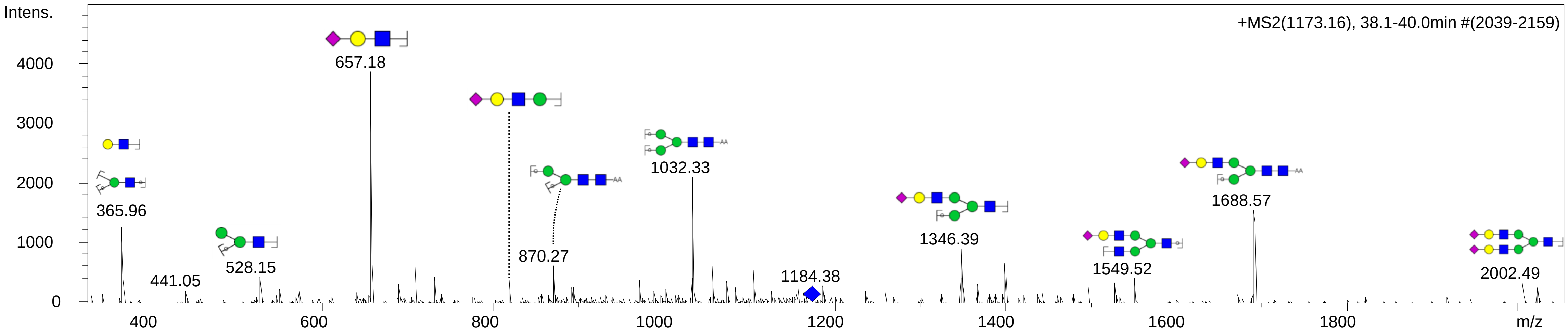

**Spectrum 36** 1181.68 (H6N4SA1F1-AA++)

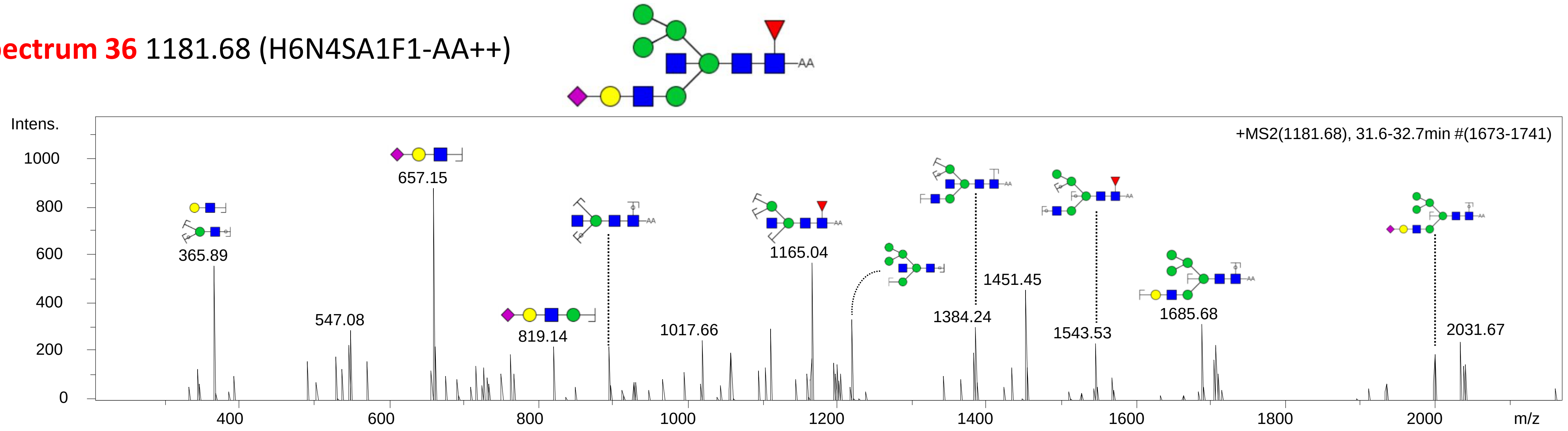


**Spectrum 37** 1202.33 (H5N5SA1F1-AA++)

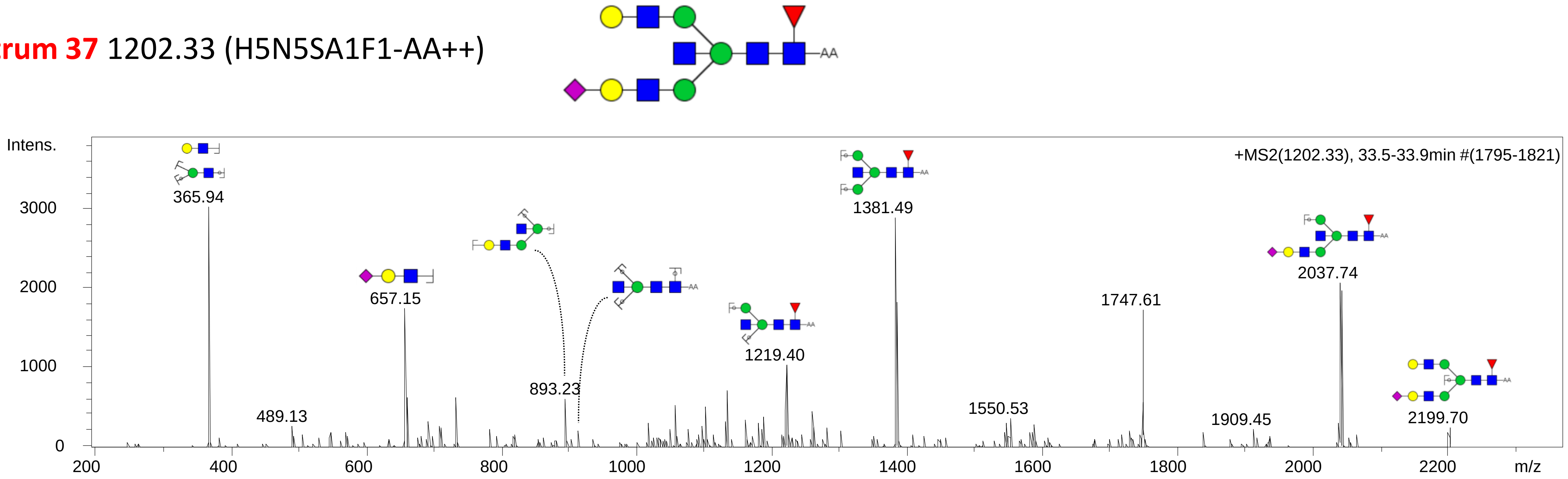

## Spectrum 38 1246.55 (H5N4SA2F1-AA++)

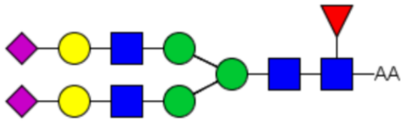


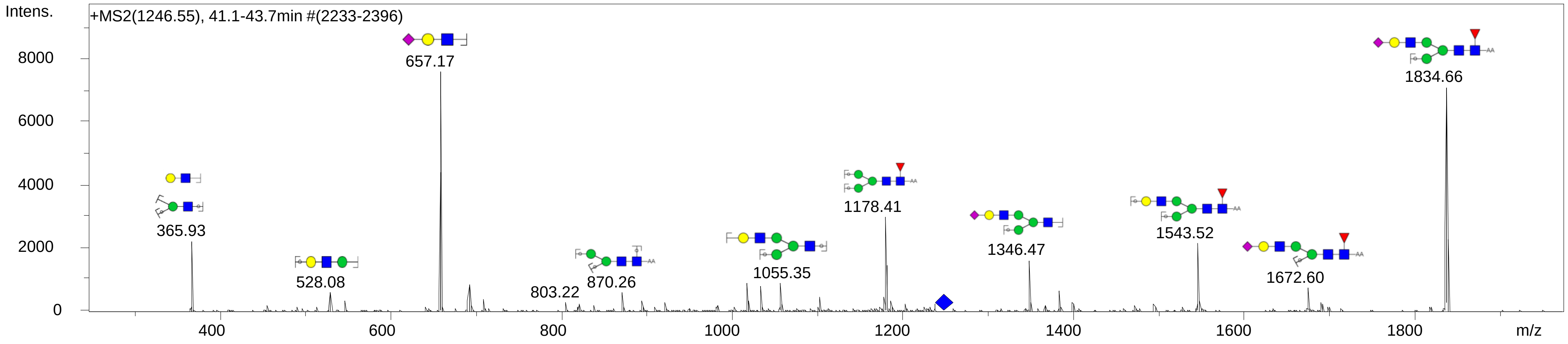

**Spectrum 39** 1274.47 (H5N5SA2-AA++)

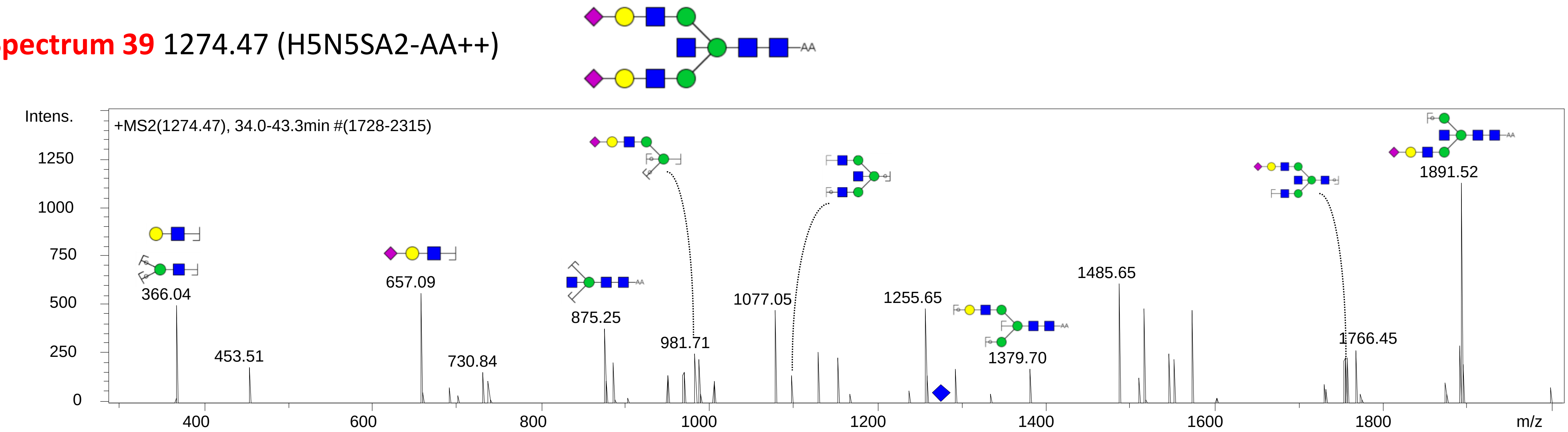

# **Spectrum 40** 898.82 (H5N5SA2F1-AA+++)

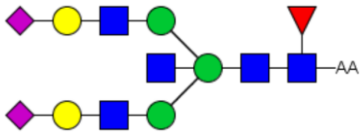

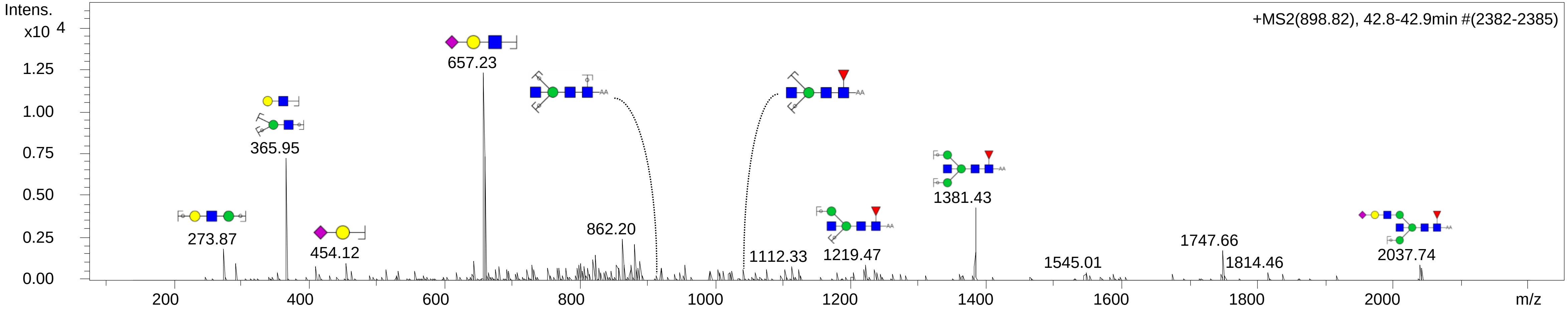

**Spectrum 41** 923.75 (H6N6SA1F1-AA+++)

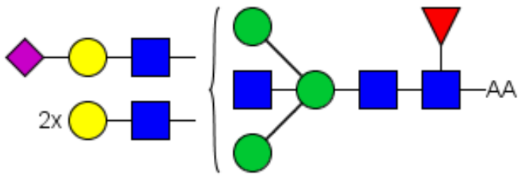


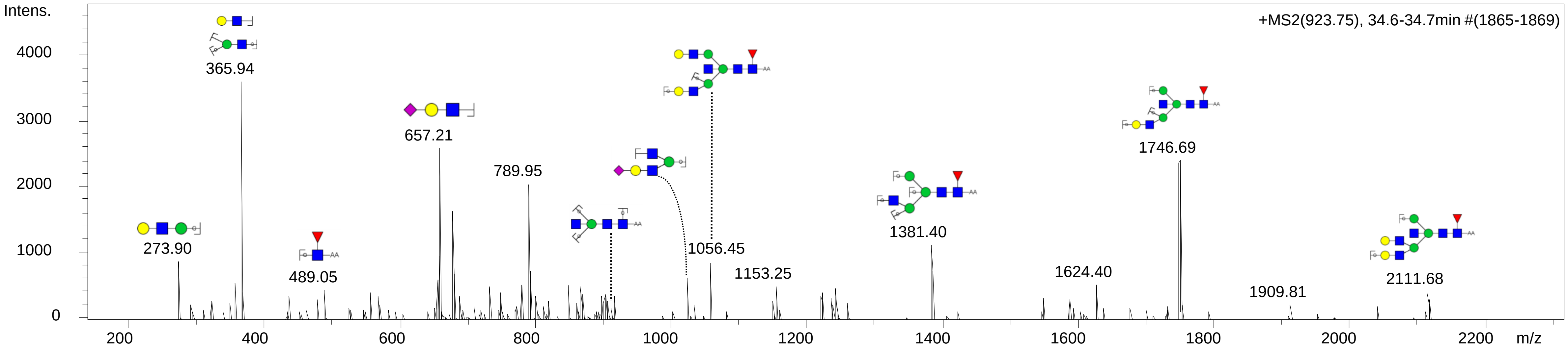


**Spectrum 42** 953.17 (H6N5SA2F1-AA++)

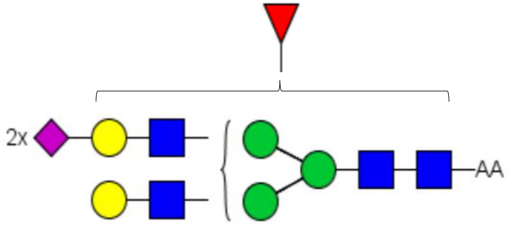


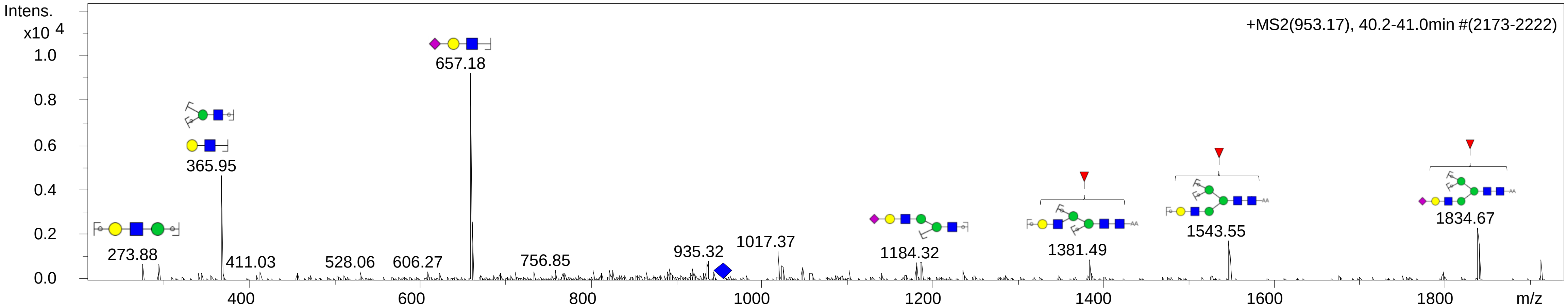

**Spectrum 43** 977.56 (H7N6SA1F1-AA+++)

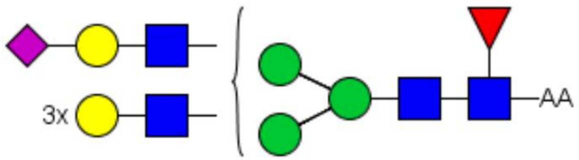


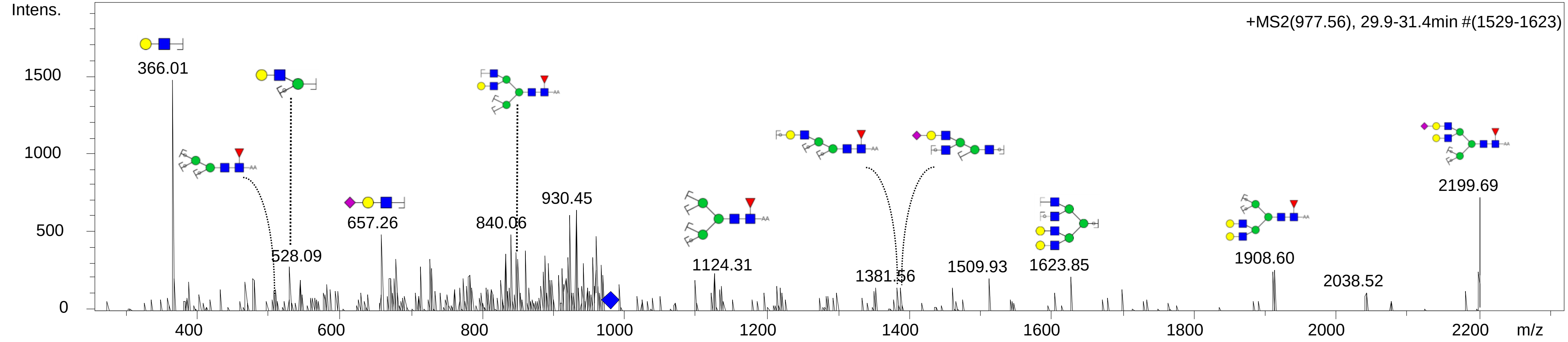


**Spectrum 44** 1021.02 (H6N6SA2F1-AA+++)

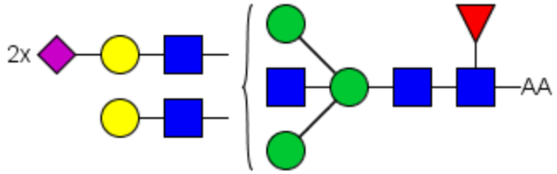


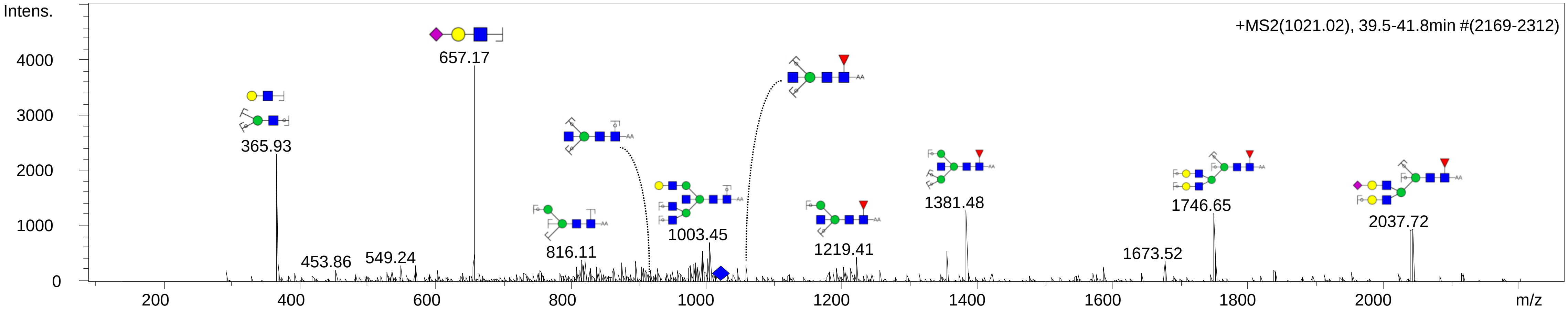

**Spectrum 45** 1046.07 (H7N7SA1F1-AA+++)

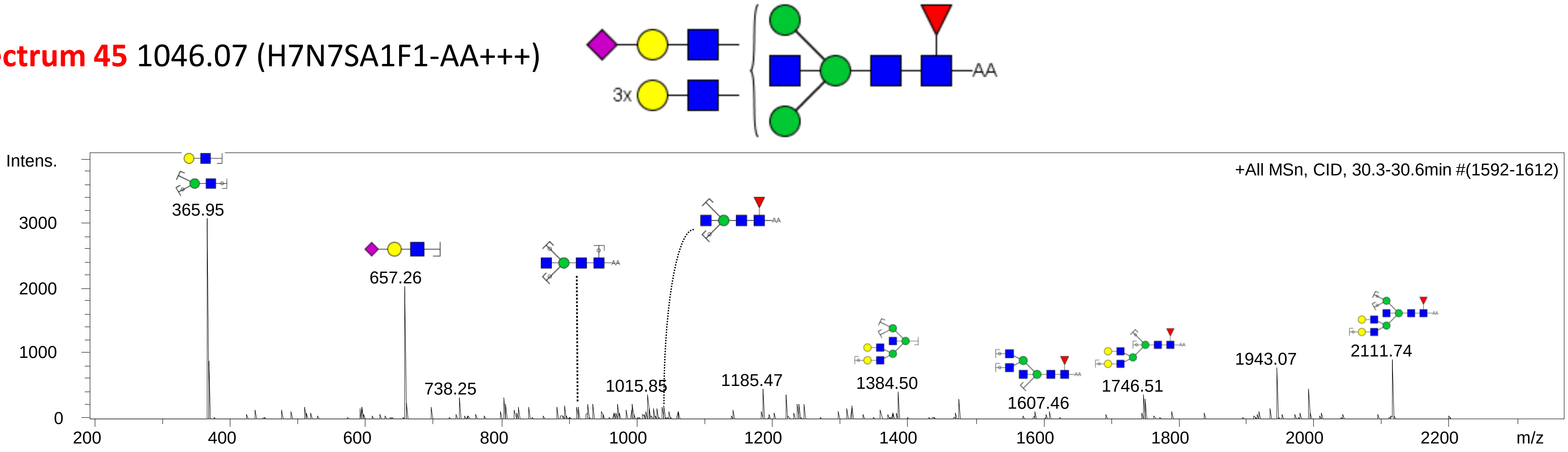


**Spectrum 46** 1050.10 (H6N5SA3F1-AA +++)

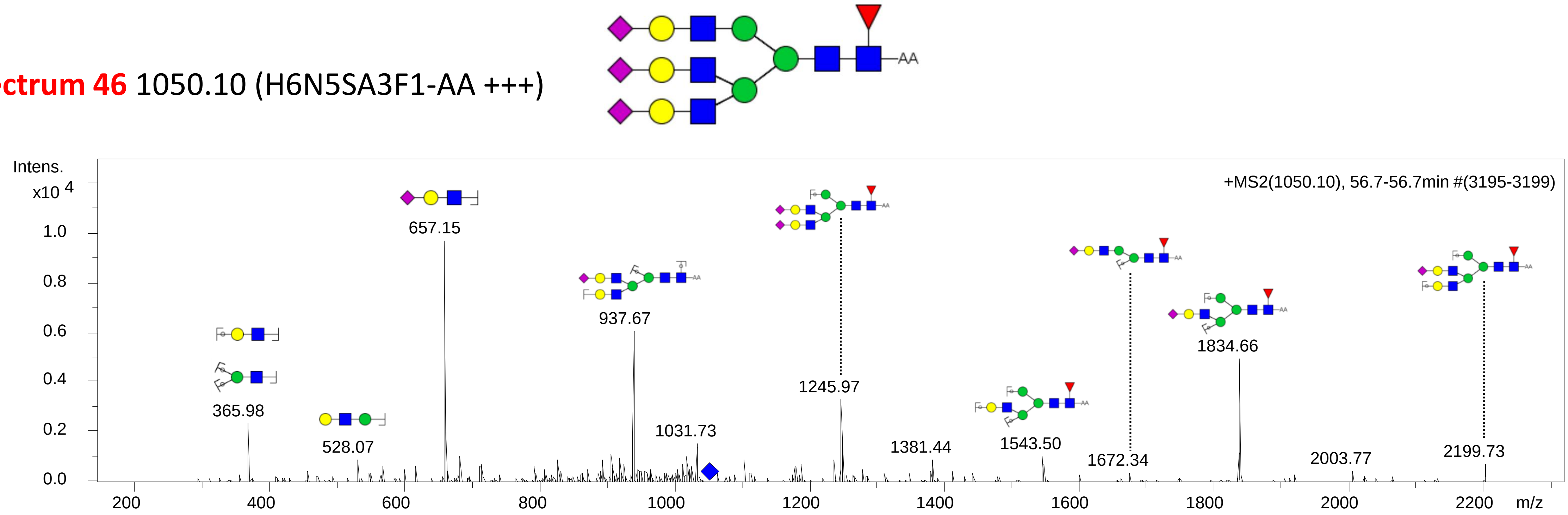

**Spectrum 47** 1074.90 (H7N6SA2F1-AA+++)

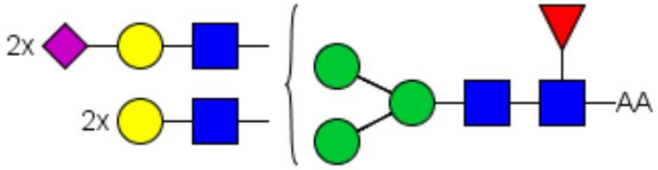


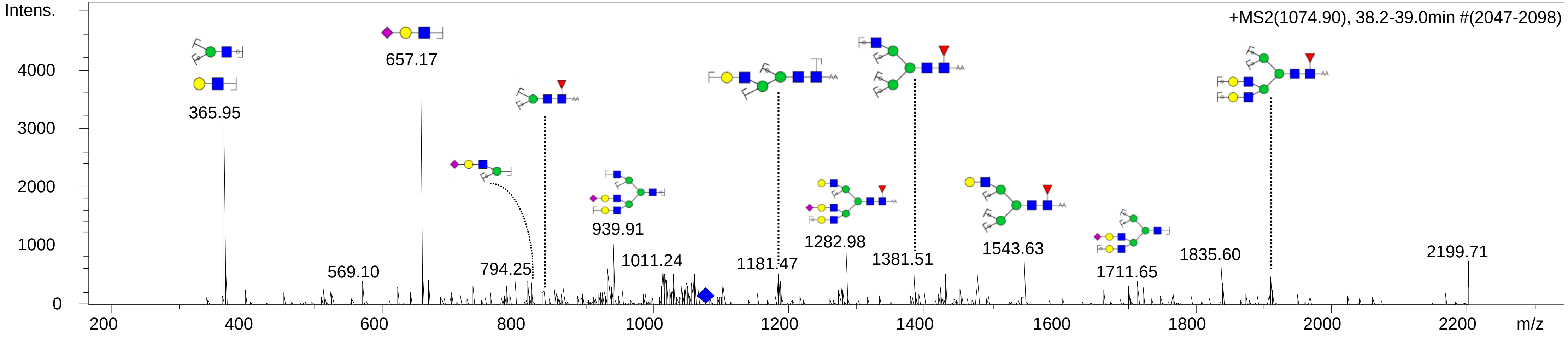

**Spectrum 48** 1172.03 (H7N6SA3F1-AA+++)

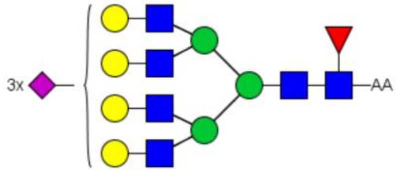


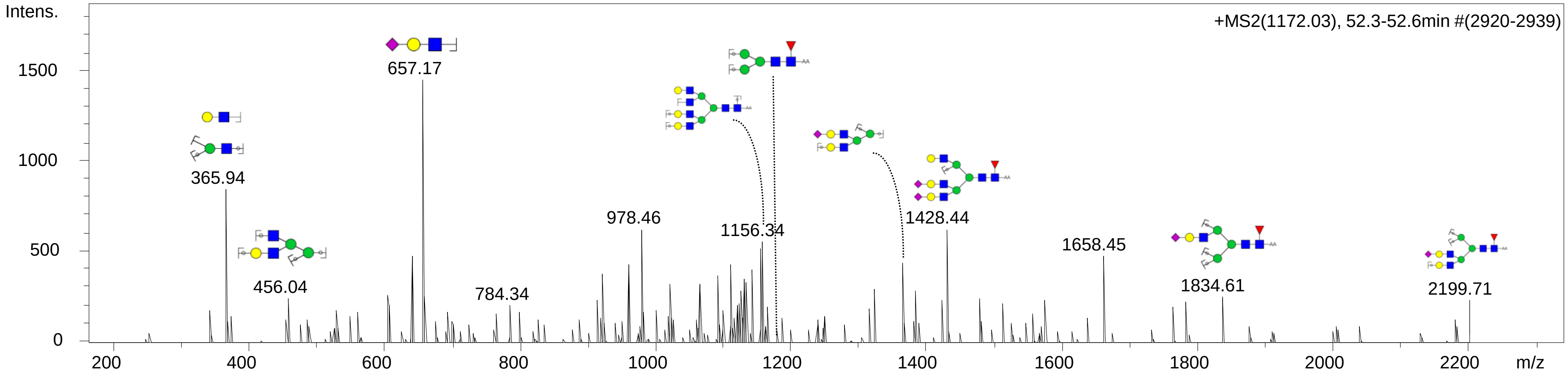

Supplement: Supplementary file 3 — (PDF 1991 kb) [file 10719_2018_9814_MOESM3_ESM.pdf]
