# Supplementary material for: The glycomic effect of N-acetylglucosaminyltransferase III overexpression in metastatic melanoma cells. GnT-III modifies highly branched N-glycans
Source: Glycoconj J. 2018 Mar 3;35(2):217–31. doi: 10.1007/s10719-018-9814-y (PMC5916991; doi:10.1007/s10719-018-9814-y)

Supplementary Figure 2. N-glycans expression on membrane proteins of WM266-4 melanoma cells, before and after transfection with the empty plasmid. Comparison of negative ion mode MALDI-TOF-MS spectra of AA-labeled *N*-glycans released from membrane proteins of parent (WM266-4, black line) and mock-transfected (WM266-4-pIRESneo, red line) melanoma cells.

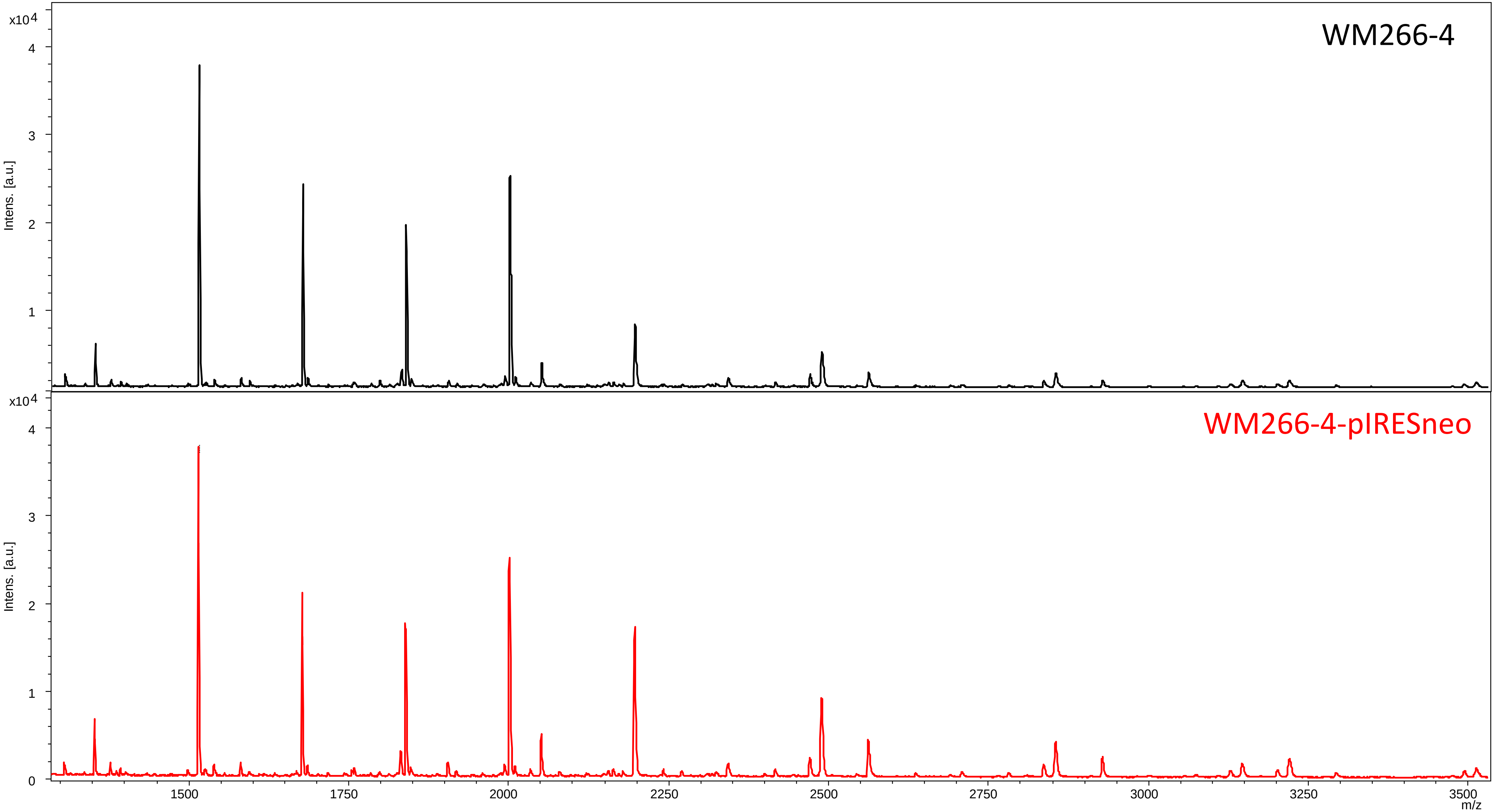

Supplement: Supplementary file 4 — (PDF 178 kb) [file 10719_2018_9814_MOESM4_ESM.pdf]
